# Coronary artery calcium score and pre-test probabilities as gatekeepers to predict and rule out perfusion defects in positron emission tomography

**DOI:** 10.1007/s12350-023-03322-3

**Published:** 2023-07-06

**Authors:** Olivier F. Clerc, Simon M. Frey, Ursina Honegger, Melissa L. F. Amrein, Federico Caobelli, Philip Haaf, Michael J. Zellweger

**Affiliations:** 1 Department of Cardiology, University Hospital Basel, University of Basel, Petersgraben 4, 4031 Basel, Switzerland; 2 Cardiovascular Research Institute Basel (CRIB), University Hospital Basel, University of Basel, Basel, Switzerland; 3 Department of Nuclear Medicine, Clinic of Radiology and Nuclear Medicine, University Hospital Basel, University of Basel, Basel, Switzerland

## Abstract

**Background:**

Little is known about the gatekeeper performance of coronary artery calcium score (CACS) before myocardial perfusion positron emission tomography (PET), compared with updated pre-test probabilities from American and European guidelines (pre-test-AHA/ACC, pre-test-ESC).

**Methods:**

We enrolled participants without known coronary artery disease undergoing CACS and Rubidium-82 PET. Abnormal perfusion was defined as summed stress score ≥ 4. Using Bayes’ formula, pre-test probabilities and CACS were combined into post-test probabilities.

**Results:**

We included 2050 participants (54% male, mean age 64.6 years) with median CACS 62 (IQR 0-380), pre-test-ESC 17% (11-26), pre-test-AHA/ACC 27% (16-44), and abnormal perfusion in 437 participants (21%). To predict abnormal perfusion, area under the curve of CACS was 0.81, pre-test-AHA/ACC 0.68, pre-test-ESC 0.69, post-test-AHA/ACC 0.80, and post-test-ESC 0.81 (*P* < 0.001 for CACS vs. each pre-test, and each post-test vs. pre-test). CACS = 0 had 97% negative predictive value (NPV), pre-test-AHA/ACC ≤ 5% 100%, pre-test-ESC ≤ 5% 98%, post-test-AHA/ACC ≤ 5% 98%, and post-test-ESC ≤ 5% 96%. Among participants, 26% had CACS = 0, 2% pre-test-AHA/ACC ≤ 5%, 7% pre-test-ESC ≤ 5%, 23% post-test-AHA/ACC ≤ 5%, and 33% post-test-ESC ≤ 5% (all *P* < 0.001).

**Conclusions:**

CACS and post-test probabilities are excellent predictors of abnormal perfusion and can rule it out with very high NPV in a substantial proportion of participants. CACS and post-test probabilities may be used as gatekeepers before advanced imaging.

**Graphical Summary: Prediction of Abnormal Perfusion in Position Emission Tomography Using Coronary Artery Calcium Score and Pre-Test Probabilities:**

Coronary artery calcium score (CACS) predicted abnormal perfusion (SSS ≥ 4) in myocardial positron emission tomography (PET) better than pre-test probabilities of coronary artery disease (CAD), while pre-test-AHA/ACC and pre-test-ESC performed similarly (left). Using Bayes’ formula, pre-test-AHA/ACC or pre-test-ESC were combined with CACS into post-test probabilities (middle). This calculation reclassified a substantial proportion of participants to low probability of CAD (0-5%), not needing further imaging, as shown for AHA/ACC probabilities (2% with pre-test-AHA/ACC to 23% with post-test-AHA/ACC, *P *< 0.001, right). Very few participants with abnormal perfusion were classified under pre-test or post-test probabilities 0-5%, or under CACS 0. AUC: area under the curve. Pre-test-AHA/ACC: Pre-test probability of the American Heart Association/American College of Cardiology. Post-test-AHA/ACC: Post-test probability combining pre-test-AHA/ACC and CACS. Pre-test-ESC: Pre-test probability of the European Society of Cardiology. SSS: Summed stress score.

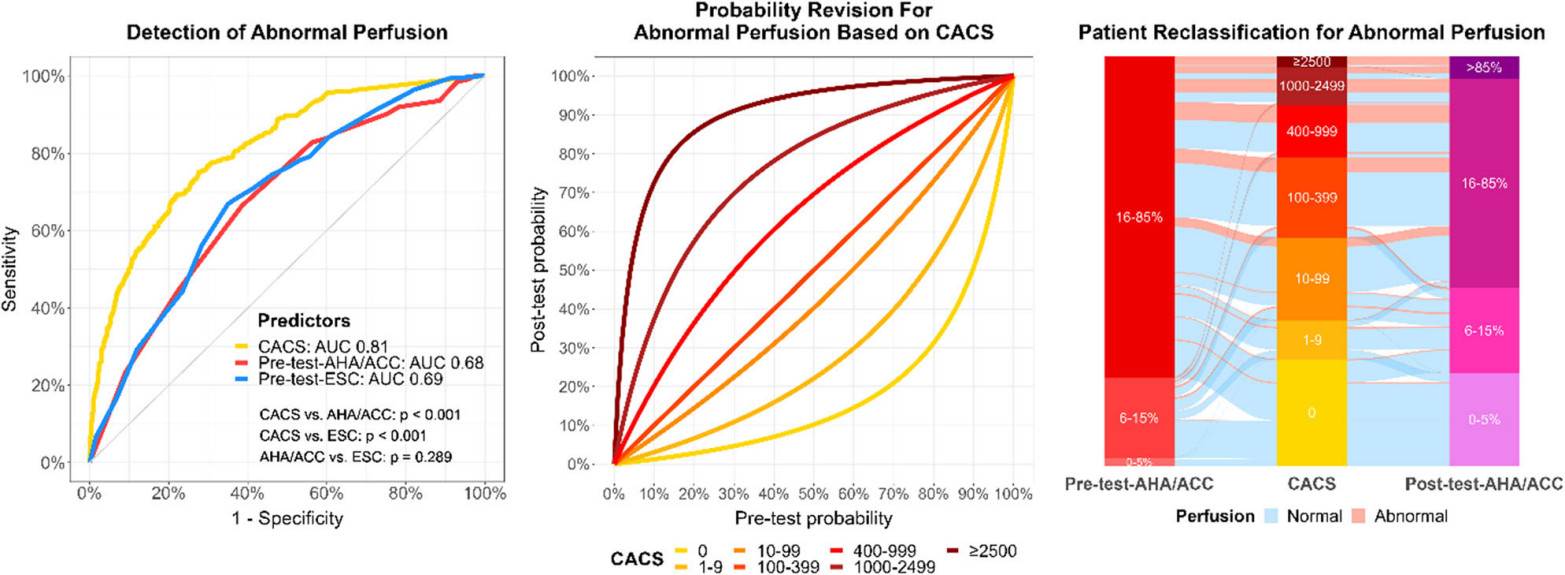

**Supplementary Information:**

The online version contains supplementary material available at 10.1007/s12350-023-03322-3.

## Introduction

Coronary artery disease (CAD) is the leading cause of mortality and the second cause of disability worldwide, with 9 million deaths and 182 million disability-adjusted life-years annually.^1^ In patients with suspected CAD, clinical prediction rules are recommended to assess the probability of obstructive CAD, such as pre-test probabilities from the American Heart Association and the American College of Cardiology (pre-test-AHA/ACC) or from the European Society of Cardiology (pre-test-ESC).^2,3^ Both pre-test probabilities are based on the Diamond-Forrester model with age, gender and symptoms, and were recently updated using current data form large cohorts, mainly using coronary computed tomography (CT).^4^ Depending on pre-test probability, non-invasive testing for CAD may be recommended.^2,3^

Myocardial perfusion positron emission tomography (PET) is a relatively recent non-invasive test for CAD with superior diagnostic and prognostic accuracy.^5–9^ PET can assess both relative and absolute myocardial perfusion, including myocardial flow reserve (MFR), which provides additional diagnostic and prognostic value.^6,10–14^ PET was shown to be cost-effective for CAD, but it is an expensive test.^15,16^

The coronary artery calcium score (CACS) is a simple and comparatively inexpensive measurement of coronary calcifications on CT.^17^ CACS is an excellent predictor of short-term and long-term adverse cardiac events and mortality.^5,9,11,12,14,18–23^ CACS can also predict myocardial ischemia on single-photon emission computed tomography (SPECT),^18,24^ but studies on prediction of PET findings are conflicting.^5,12,14,25–27^ Thus, CACS might be an effective gatekeeper to rule out obstructive CAD before advanced and expensive imaging tests. This may prove particularly advantageous, given the currently low frequency of abnormal myocardial perfusion test results.^4,28^

However, using CACS as a gatekeeper is controversial and more evidence is needed.^29,30^ Moreover, the comparative prediction and rule out abilities of CACS vs. the recently updated pre-test-AHA/ACC and pre-test-ESC for PET perfusion defects are unknown. Therefore, the aims of this study were to compare CACS, pre-test-probabilities, and combinations of them (post-test probabilities) to predict perfusion defects in patients with suspected CAD undergoing PET, and to compare the gatekeeper performance of CACS 0, pre-test probabilities ≤ 5% and post-test probabilities ≤ 5%, according to guidelines.^2,3^

## Methods

### Study design

This was an observational, cross-sectional study of prospectively acquired data, performed at the University Hospital of Basel, Switzerland. It was approved by the institutional review board (Req-2020-00283) and conducted in accordance with the declaration of Helsinki. The manuscript was written following the Standards for Reporting of Diagnostic Accuracy (STARD) guidelines.^31^

All consecutive patients undergoing Rubidium-82 myocardial perfusion PET with CACS from 2016 to 2021 at our center were enrolled. We excluded patients with known CAD (e.g., prior myocardial infarction or revascularization) and with missing PET or CACS data. Demographic variables, cardiovascular risk factors, cardiac symptoms, and imaging data were recorded. Pre-test-AHA/ACC and pre-test-ESC were calculated based on age, gender, and symptoms, then categorized using pre-specified cut-offs recommended to consider imaging (> 5%), to perform imaging (> 15%), and to rule in CAD (> 85%).^2,3,7^ Both give pre-test probabilities 0-52%, without rule-in for CAD (no value > 85%). But they differ regarding chest pain: while pre-test-ESC attributes different pre-test probabilities to typical, atypical, and non-anginal chest pain, pre-test AHA/ACC uses probabilities of typical chest pain for any chest pain, resulting in higher predicted values.

### Imaging

All participants underwent Rubidium-82 myocardial perfusion PET with rest/stress protocol and low-dose CT for CACS on a PET/CT system (Biograph mCT, Siemens, Erlangen, Germany). Details of our imaging protocol have been previously published.^13^ Stress was induced using intravenous adenosine, or regadenoson in case of obstructive respiratory disease.

Images were interpreted in consensus by a nuclear medicine physician and a cardiologist, both board certified and with numerous years of experience. CACS was measured in Agatston units, based on areas ≥ 1 mm^2^ with density ≥ 130 Hounsfield units.^17^ CACS was categorized using traditional cut-offs at 0, 10, 100, 400, 1000, and a high cut-off at 2500 Agatston units.^24^ PET perfusion images at rest and stress were visually assessed and scored on a 17-segment model (0: no defect; 1: mildly reduced; 2: moderately reduced; 3: severely reduced; and 4: absent tracer uptake), as recommended.^32^ We calculated the summed rest score (SRS), summed stress score (SSS), and summed difference score (SDS = SSS – SRS).^32^ Global myocardial blood flow and MFR were computed using proprietary software (Syngo MBF, Siemens, Erlangen, Germany).

### Endpoint definition

Our primary endpoint was abnormal perfusion on PET, defined as SSS ≥ 4, based on imaging guidelines.^32^ As our secondary endpoint, severe ischemia was defined as SDS ≥ 8, corresponding to ischemia > 10% of the left ventricular myocardium, predicting worse outcomes and potential benefit from revascularization.^33^ Moreover, we defined low MFR as < 2.0, which predicts high-risk CAD or microvascular disease, and adverse outcomes.^6,10^

### Statistical analysis

Continuous variables were presented as mean with standard deviation (SD) and compared using *t*-test if normally distributed, or as median with interquartile range (IQR) and Wilcoxon rank-sum test if non-normally distributed. Categorical variables were presented as frequencies with percentages, and compared using Fisher’s exact test. Paired comparisons of diagnostic categories were performed with McNemar’s test. Trends were assessed with the Cochran-Armitage test. Receiver operating characteristic (ROC) analysis was used to measure discrimination capacity as area under the curve (AUC), with 95% confidence interval (CI), compared using DeLong’s method. Independent associations between CACS, pre-test probabilities and PET findings were evaluated using multivariable logistic regression, adjusted for demographics, cardiovascular risk factors, and symptoms. We calculated sensitivity, specificity, positive and negative predictive values (PPV, NPV), positive and negative likelihood ratios at pre-specified diagnostic thresholds with 95% CI using Wilson’s method. Calibration was examined on calibration plots. Discrimination capacity was further evaluated using the integrated discrimination improvement (IDI). Participant reclassification across probability cut-offs was analyzed with the net reclassification improvement (NRI). To combine pre-test-ESC or pre-test-AHA/ACC with CACS into post-test probabilities, we calculated interval likelihood ratios from CACS thresholds and used Bayes’ likelihood ratio formula: pre-test probability/(1 − pre-test probability) = pre-test odds; pre-test odds x interval likelihood ratio = post-test odds; post-test odds/(1 + post-test odds) = post-test probability. Analyses were conducted using R version 4.2.2 (R Core Team, R Foundation for Statistical Computing, Vienna, Austria), using the packages *tidyverse*, *DescTools*, *tableone*, *rstatix*, *pROC*, *ROCit*, *PredictABEL*, and *ggalluvial*. *P* values were two-sided and considered as statistically significant if < 0.05.

## Results

### Descriptive analysis

**Figure 1:**
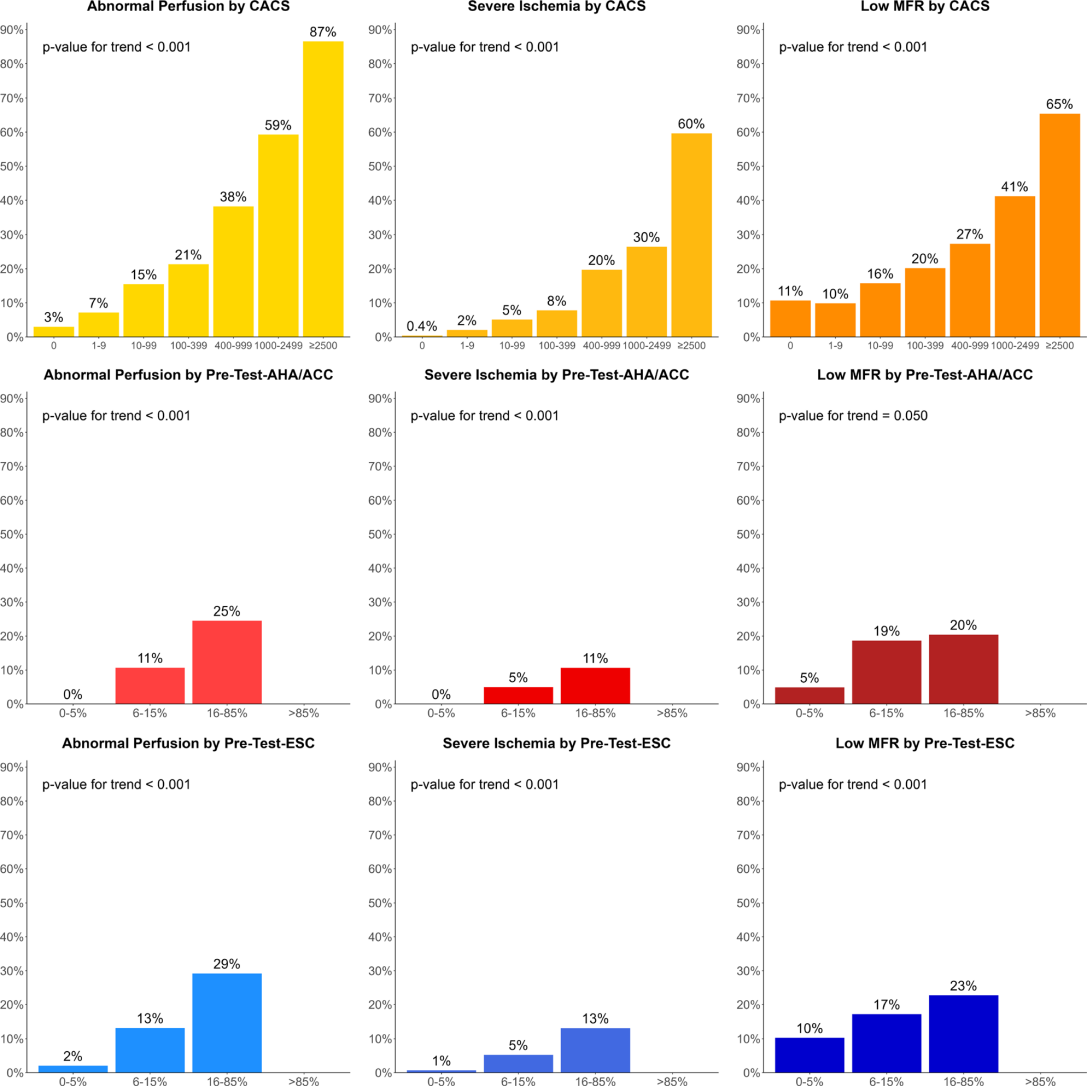
Prevalence of endpoints by CACS and pre-test probability categories. The prevalence of all endpoints increased across categories of predictors. Endpoints were rare in the lowest category of each predictor, except low MFR. Pre-test probabilities cannot be > 52% and therefore do not reach rule-in levels for coronary artery disease (> 85%). Only high CACS identifies patients with high prevalence of endpoints. *CACS*, coronary artery calcium score; *MFR*, myocardial flow reserve; *pre-test-AHA/ACC*, pre-test probability from American Heart Association/American College of Cardiology guidelines; *pre-test-ESC*, pre-test probability from European Society of Cardiology guidelines

From 4049 patients in our prospective PET database, 1984 (49%) were excluded for known CAD and 15 for missing imaging data (0.4%, Supplementary Figure A1). Thus, we included 2050 participants, with 1112 men (54%) and mean age 64.6 years (SD 11.2). Median CACS was 62 (IQR 0-380), median pre-test-AHA/ACC 27% (16-44), and median pre-test-ESC 17% (11-26). On PET, 437 participants had abnormal perfusion (21%), 190 severe ischemia (9%), and 403 low MFR (20%). Participants with abnormal perfusion were significantly older, more often males, had more cardiovascular risk factors and typical angina, higher CACS, pre-test-AHA/ACC, and pre-test-ESC (Table 1). The prevalence of abnormal perfusion, severe ischemia and low MFR increased across categories of CACS, pre-test-AHA/ACC, and pre-test-ESC (Figure 1). In the lowest category of each of these predictors, abnormal perfusion was rare (≤ 3%) and severe ischemia very rare (≤ 1%). However, low MFR was observed in a substantial proportion of participants within the lowest categories (5-11%). Regarding high probabilities, only CACS ≥ 2500 identified participants with high prevalence of PET findings (≥ 60%).

**Table 1:** Patient characteristics

Characteristics	Overall	Normal perfusion (SSS < 4)	Abnormal perfusion (SSS ≥ 4)	*P* value
Number	2050	1613	437	
Demographics and risk factors				
Age (years)	64.6 (11.2)	63.5 (11.3)	68.7 (9.8)	< 0.001
Body mass index (kg/m^2^)	28.7 (6.2)	28.8 (6.5)	28.1 (4.8)	0.024
Male gender	1112 (54%)	783 (49%)	329 (75%)	< 0.001
Insulin-dependent DM	139 (7%)	97 (6%)	42 (10%)	0.010
Non-insulin dependent DM	353 (17%)	263 (16%)	90 (21%)	0.038
Hypercholesterolemia	1026 (50%)	780 (48%)	246 (56%)	0.004
Arterial hypertension	1401 (68%)	1065 (66%)	336 (77%)	< 0.001
Active smoking	463 (23%)	355 (22%)	108 (25%)	0.246
Previous smoking	556 (27%)	416 (26%)	140 (32%)	0.011
Family history of CAD	510 (25%)	412 (26%)	98 (22%)	0.191
Main symptom				
Typical angina pectoris	391 (19%)	295 (18%)	96 (22%)	0.086
Atypical angina pectoris	565 (28%)	482 (30%)	83 (19%)	< 0.001
Non-anginal pain	701 (34%)	549 (34%)	152 (35%)	0.690
Dyspnea	393 (19%)	287 (18%)	106 (24%)	0.005
Predictors				
CACS*	62 (0–380)	27 (0–211)	601 (168–1453)	< 0.001
Pre-test-AHA/ACC (%)*	27 (16–44)	27 (16–32)	32 (27–44)	< 0.001
Pre-test-ESC (%)*	17 (11–26)	16 (10–24)	24 (16–32)	< 0.001
PET myocardial perfusion				
SSS*	0 (0–1)	0 (0–0)	8 (5–13)	< 0.001
SRS*	0 (0–0)	0 (0–0)	0 (0–3)	< 0.001
SDS*	0 (0–0)	0 (0–0)	6 (4–10)	< 0.001
PET myocardial flow (ml/g/min)				
Rest	1.3 (0.5)	1.4 (0.5)	1.2 (0.4)	< 0.001
Stress	3.3 (0.9)	3.5 (0.8)	2.4 (0.8)	< 0.001
MFR	2.7 (0.9)	2.8 (0.9)	2.2 (0.8)	< 0.001
Low MFR (< 2.0)	403 (20%)	219 (14%)	184 (43%)	< 0.001

Continuous variables are presented as mean (standard deviation) with *P* value from *t*-test, and categorical variables as frequency (percentage) with *P* value from Fisher’s exact test.

*Continuous variables with non-normal distribution are presented as median (interquartile range) with *P* value from Wilcoxon rank-sum test.

*CAD*, Coronary artery disease; *DM*, diabetes mellitus; *MFR*, myocardial flow reserve; *CACS*, coronary artery calcium score; *PET*, Positron emission tomography; *pre-test-AHA/ACC*, pre-test probability from American Heart Association/American College of Cardiology guidelines; *pre-test-ESC*, pre-test probability from European Society of Cardiology guidelines; *SDS*, summed difference score; *SRS*, summed rest score; *SSS*, summed stress score

### Discrimination capacity

**Figure 2:**
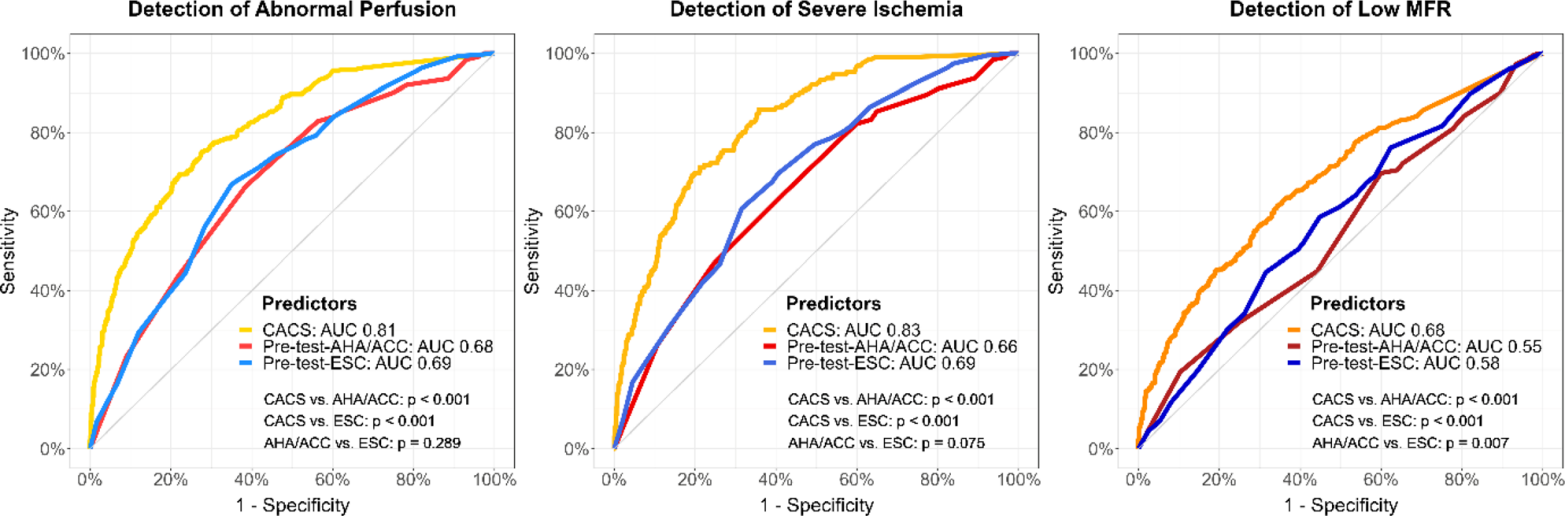
Receiver operating characteristic analysis. To predict all endpoints, CACS had a significantly higher AUC than pre-test probabilities. To predict low MFR, pre-test-ESC had a significantly higher AUC than pre-test-AHA/ACC, but pre-test probabilities did not differ from each other for abnormal perfusion and severe ischemia. *AUC*, area under the curve; *CACS*, coronary artery calcium score; *MFR*, myocardial flow reserve; *pre-test-AHA/ACC*, pre-test probability from American Heart Association/American College of Cardiology guidelines; *pre-test-ESC*, pre-test probability from European Society of Cardiology guidelines

In ROC analysis, CACS had an AUC of 0.81 (95% CI 0.79-0.83) for abnormal perfusion, 0.83 (0.80-0.85) for severe ischemia, and 0.68 (0.65-0.71) for low MFR, significantly higher than corresponding AUC from pre-test probabilities (all *P* < 0.001, Figure 2). Pre-test-AHA/ACC and pre-test-ESC had similar AUC, except a higher AUC with pre-test-ESC for low MFR (*P* = 0.007). In multivariable logistic regressions, CACS was independently associated with all endpoints (all *P* < 0.001), while pre-test-AHA/ACC and pre-test-ESC were only independently associated with abnormal perfusion and severe ischemia (both *P* ≤ 0.003), not with low MFR (Supplementary Tables A1a-A1c). Abnormal perfusion and severe ischemia were most significantly associated with male gender, typical angina pectoris and CACS. However, low MFR was most significantly associated with age, female gender, diabetes mellitus, and CACS. This difference is probably due to the ability of low MFR to detect microvascular disease in addition to macrovascular CAD. Therefore, and considering the limited discrimination capacity of our predictors for low MFR, we conducted the next analyses for abnormal perfusion and severe ischemia only.

### Diagnostic accuracy

In threshold analysis, we found a high predictive performance of the lowest category of each predictor (Table 2). CACS 0 had 96% sensitivity for abnormal perfusion (95% CI 94-98%) with 97% NPV (95-98%), and 99% sensitivity for severe ischemia (96-100%) with > 99% NPV (99-100%). Pre-test-AHA/ACC ≤ 5% had 100% sensitivity and NPV for abnormal perfusion (99-100%; 91-100%), and 100% sensitivity and NPV for severe ischemia (98-100%; 91-100%). Pre-test-ESC ≤ 5% had 99% sensitivity for abnormal perfusion (98-100%) with 98% NPV (94-99%), and 99% sensitivity for severe ischemia (97-100%) with 99% NPV (96-100%). However, only 2% of participants had pre-test-AHA/ACC ≤ 5% and 7% had pre-test-ESC ≤ 5%, while 26% had CACS 0 (all *P* < 0.001). Moreover, CACS ≥ 2500 had > 99% specificity for abnormal perfusion (99-100%) with 87% PPV (rule-in level, 75-93%), and 99% specificity for severe ischemia (98-99%) with 60% PPV (46-72%).

**Table 2:** Threshold table for pre-test probabilities and coronary artery calcium score

Threshold	Cumulative proportion	Sensitivity	Specificity	PPV	NPV	PLR	NLR
Abnormal perfusion (SSS ≥ 4)							
Coronary artery calcium score							
0	26.1%	96.3%	32.2%	27.8%	97.0%	1.42	0.11
10	35.7%	93.1%	43.5%	30.9%	95.9%	1.65	0.16
100	55.9%	78.5%	65.2%	37.9%	91.8%	2.26	0.33
400	75.4%	59.0%	84.7%	51.1%	88.4%	3.86	0.48
1000	88.2%	35.9%	94.8%	65.1%	84.5%	6.90	0.68
2500	97.5%	10.3%	99.6%	86.5%	80.4%	23.73	0.90
Pre-test probability AHA/ACC							
5%	2.0%	100%	2.5%	21.8%	100%	1.03	0.00
15%	21.7%	90.2%	24.9%	24.5%	90.3%	1.20	0.40
85%	100%	–	–	–	–	–	–
Pre-test probability ESC							
5%	7.1%	99.3%	8.9%	22.8%	97.9%	1.09	0.08
15%	44.2%	76.4%	49.8%	29.2%	88.6%	1.52	0.47
85%	100%	–	–	–	–	–	–
Severe ischemia (SDS ≥ 8)							
Coronary artery calcium score							
0	26.1%	99.0%	28.7%	12.5%	99.6%	1.39	0.04
10	35.7%	96.9%	39.1%	14.0%	99.2%	1.59	0.08
100	55.9%	85.9%	60.2%	18.1%	97.6%	2.16	0.23
400	75.4%	69.6%	80.0%	26.3%	96.2%	3.48	0.38
1000	88.2%	42.4%	91.4%	33.6%	93.9%	4.93	0.63
2500	97.5%	16.2%	98.9%	59.6%	92.0%	14.37	0.85
Pre-test probability AHA/ACC							
5%	2.0%	100%	2.2%	9.5%	100%	1.02	0.00
15%	21.7%	89.5%	22.8%	10.6%	95.5%	1.16	0.46
85%	100%	–	–	–	–	–	–
Pre-test probability ESC							
5%	7.1%	99.5%	7.8%	10.0%	99.3%	1.08	0.07
15%	44.2%	78.5%	46.5%	13.1%	95.5%	1.47	0.46
85%	100%	–	–	–	–	–	–

*AHA/ACC*, American Heart Association/American College of Cardiology; *ESC*, European Society of Cardiology; *NLR*, negative likelihood ratio; *NPV*, negative predictive value; *PLR*, positive likelihood ratio; *PPV*, positive predictive value; *SDS*, summed difference score; *SSS*, summed stress score

### Bayesian analysis

**Figure 3:**
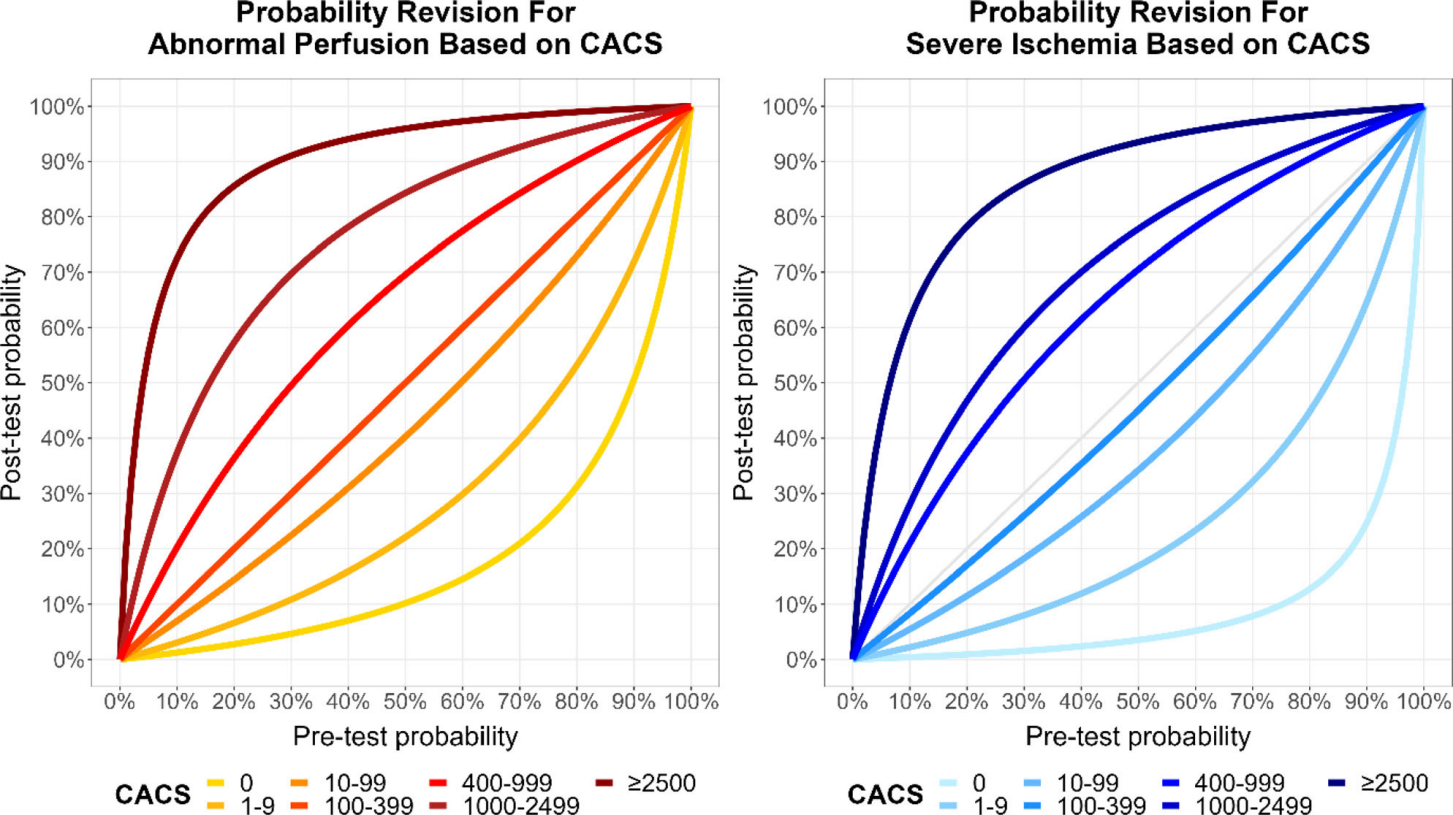
Bayesian probability revision using coronary artery calcium score. Probability revision curves were calculated with Bayes’ formula and interval likelihood ratios for CACS. To use these graphs, start on the x-axis at the pre-test probability (by any method), move up to the line for measured CACS, then move left to the post-test probability on the y-axis. CACS 0 and ≥ 2500 have a strong impact on post-test probabilities, particularly CACS 0 for severe ischemia (post-test probability < 5% for pre-test probabilities up to 52%, the maximal value). *CACS*, coronary artery calcium score

**Figure 4:**
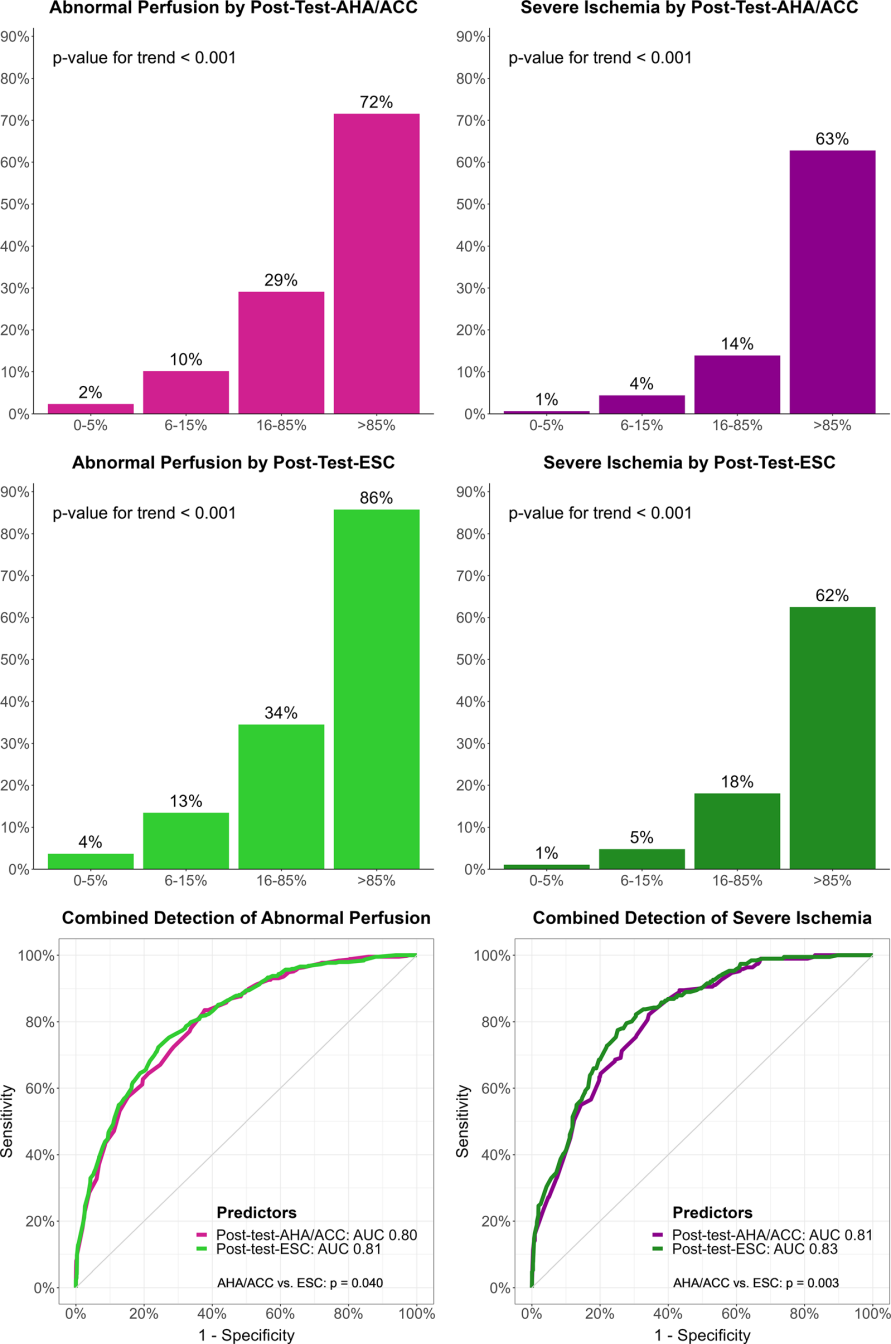
Bayesian combined post-test probabilities. Pre-test probabilities from AHA/ACC or ESC were combined with CACS using Bayes’ formula into post-test probabilities. Endpoint prevalence increased steeply across categories, up to rule-in levels for coronary artery disease. AUC were significantly higher with post-test-ESC than with post-test-AHA/ACC. *AUC*, area under the curve; *CACS*, coronary artery calcium score; *post-test-AHA/ACC*, post-test probability from American Heart Association/American College of Cardiology guidelines; *post-test-ESC*, pre-test probability from European Society of Cardiology guidelines

**Figure 5:**
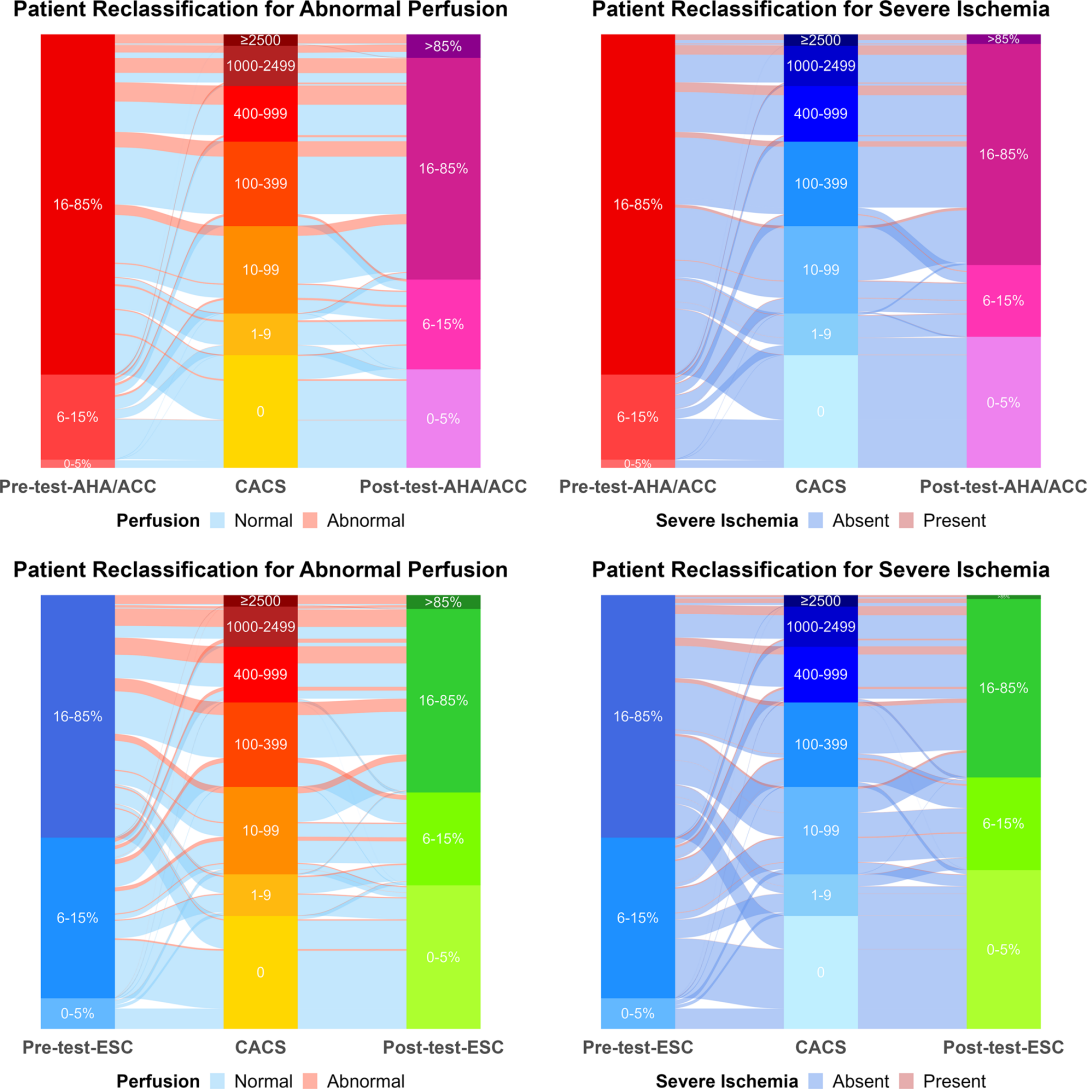
Patient reclassification by predictors. Patient flows through categories of pre-test probability, CACS, and post-test probability are shown by endpoint. More patients were classified as low probability of CAD with ESC vs. AHA/ACC probabilities, and with post-test vs. pre-test probabilities (all *P* < 0.001 using McNemar’s test). Very few participants with abnormal perfusion or severe ischemia were classified under pre-test or post-test probabilities 0-5%, or under CACS 0. AHA/ACC: American Heart Association/American College of Cardiology. *CACS*, coronary artery calcium score; *ESC*, European Society of Cardiology; *post-test*, post-test probability; *pre-test*, pre-test probability

Interval likelihood ratios were calculated for each CACS category (Supplementary Table A2). CACS 0 had very low values of 0.11 for abnormal perfusion and 0.04 for severe ischemia, while CACS ≥ 2500 had very high values of 23.7 for abnormal perfusion and 14.4 for severe ischemia. Using Bayes’ formula, we calculated post-test probabilities of abnormal perfusion and severe ischemia for any pre-test probability (Figure 3). The lowest and highest CACS categories had a strong impact on post-test probabilities. For example, CACS 0 reduced the post-test probability of severe ischemia to ≤ 5% for the complete range of pre-test probabilities from guidelines (0-52%). Then, we calculated post-test probabilities combining pre-test-AHA/ACC or pre-test-ESC with CACS in our participants (post-test-AHA/ACC, post-test-ESC, Figure 4). The prevalence of abnormal perfusion and severe ischemia increased steeply across post-test probability categories, up to 86% abnormal perfusion with post-test-ESC > 85% (rule-in level). In ROC analysis, AUC were slightly, but significantly higher with post-test-ESC than post-test-AHA/ACC (both *P* ≤ 0.04, Figure 4). In threshold analysis, post-test-AHA/ACC ≤ 5% had a higher sensitivity, but post-test-ESC ≤ 5% categorized more participants as low probability of CAD (Table 3, Supplementary Figure A2). For abnormal perfusion, 23% of participants had post-test-AHA/ACC ≤ 5%, with sensitivity 97% (96-99%) and NPV 98% (96-99%), while 33% had post-test-ESC ≤ 5%, with sensitivity 94% (92-96%) and NPV 96% (95-97%). For severe ischemia, 30% of participants had post-test-AHA/ACC ≤ 5%, with sensitivity 98% (95-99%) and NPV 99% (98-100%), while 37% had post-test-ESC ≤ 5%, with sensitivity 96% (92-98%) and NPV 99% (98-99%). The analysis of participant flows through categories of probabilities and CACS highlighted the higher proportion of participants classified as low probability of CAD with ESC probabilities compared with AHA/ACC probabilities (all *P* < 0.001), and the very low proportion participants with PET findings classified as low probability (Figure 5).

**Table 3:** Threshold table for post-test probabilities

Threshold	Cumulative proportion	Sensitivity	Specificity	PPV	NPV	PLR	NLR
Abnormal perfusion (SSS ≥ 4)							
Post-test probability AHA/ACC							
5%	22.9%	97.5%	28.4%	26.9%	97.7%	1.36	0.09
15%	43.6%	87.6%	52.0%	33.1%	94.0%	1.83	0.24
85%	94.7%	17.8%	98.1%	71.6%	81.5%	9.29	0.84
Post-test probability ESC							
5%	33.2%	94.3%	40.6%	30.1%	96.3%	1.59	0.14
15%	54.6%	80.8%	64.2%	38.0%	92.5%	2.26	0.30
85%	96.9%	12.4%	99.4%	85.7%	80.7%	22.15	0.88
Severe ischemia (SDS ≥ 8)							
Post-test probability AHA/ACC							
5%	30.3%	97.9%	33.2%	13.1%	99.4%	1.47	0.06
15%	46.9%	90.1%	50.7%	15.8%	98.0%	1.83	0.20
85%	97.9%	14.1%	99.1%	62.8%	91.8%	16.42	0.87
Post-test probability ESC							
5%	36.7%	95.8%	40.0%	14.1%	98.9%	1.60	0.10
15%	58.1%	84.8%	62.5%	18.9%	97.6%	2.26	0.24
85%	99.2%	5.2%	99.7%	62.5%	91.1%	16.22	0.95

*AHA/ACC*, American Heart Association/American College of Cardiology; *ESC*, European Society of Cardiology; *NLR*, negative likelihood ratio; *NPV*, negative predictive value; *PLR*, positive likelihood ratio; *PPV*, positive predictive value; *SDS*, summed difference score; *SSS*, summed stress score

### Additional analyses

For abnormal perfusion, ESC probabilities were adequately calibrated, but AHA/ACC probabilities somewhat overestimated observed probabilities (Supplementary Figure A3). Because severe ischemia was present in a subgroup of participants with abnormal perfusion, it was overestimated by all probabilities, particularly by AHA/ACC (Supplementary Figure A3). These differences in calibration limited comparisons of AHA/ACC vs. ESC probabilities using the IDI and NRI. Nevertheless, the IDI confirmed the higher discrimination capacity of post-test vs. corresponding pre-test probabilities (all *P* < 0.001, Supplementary Figure A4). Furthermore, the NRI highlighted the appropriate reclassification of numerous participants without endpoints to low-probability categories using post-test-probabilities vs. corresponding pre-test probabilities (all *P* < 0.001, Supplementary Tables A3a-A3h).

Similar analyses using the older pre-test-AHA/ACC from previous guidelines are presented in the Electronic Supplementary Materials (Part B).

## Discussion

In 2050 participants with suspected CAD undergoing PET myocardial perfusion imaging, we found a superior discrimination capacity with CACS for abnormal perfusion and severe ischemia, compared with pre-test-AHA/ACC and pre-test-ESC. CACS, pre-test-AHA/ACC and pre-test-ESC were independently associated with abnormal perfusion and severe ischemia in multivariable analyses, but only CACS was also independently associated with low MFR. CACS 0, pre-test-AHA/ACC ≤ 5% and pre-test-ESC ≤ 5% all exhibited an excellent performance to rule out abnormal perfusion (sensitivity ≥ 96% and NPV ≥ 97%) and severe ischemia (sensitivity and NPV ≥ 99%), with overlapping confidence intervals indicating no significant difference. Despite similar performance metrics, many more participants had CACS 0 than pre-test-AHA/ACC ≤ 5% or pre-test-ESC ≤ 5% (26% vs. 2% and 7%). Thus, CACS 0 identified substantially more participants at low probability of perfusion defects who may not need further imaging, with sensitivity and NPV as excellent as pre-test probabilities ≤ 5%. Moreover, CACS ≥ 2500 indicated a high probability of abnormal perfusion and severe ischemia with ≥ 99% specificity. Furthermore, combined post-test probabilities from pre-test probabilities and CACS exhibited higher discrimination capacity than pre-test probabilities and better participant classification as low probability of perfusion defects. These post-test probabilities ≤ 5% also categorized many more participants as low probability of perfusion defects than pre-test probabilities, again with excellent sensitivity and NPV. Given these similarly high diagnostic accuracy metrics, the large differences in proportions of participants categorized as low probability drive the comparative usefulness of these predictors as gatekeepers, as they determine how many participants may be reasonably discharged without further imaging. Using CACS or post-test-probabilities as gatekeepers would therefore be much more impactful than using pre-test probabilities, and serious errors would be rare (≤ 4% false negative results for abnormal perfusion and ≤ 1% for severe ischemia). Actually, severe ischemia is the most important finding, as it corresponds to an ischemia of > 10% of the left ventricular myocardium, which may trigger referral to invasive testing, and for which revascularization may be beneficial.^33^

The recommended pre-test probabilities were recently recalibrated and updated, which led to changes in AHA/ACC guidelines and ESC guidelines.^2,3^ These new pre-test probabilities are therefore more appropriate for current data than older versions used in previous works. Current guidelines are not identical: ESC guidelines attribute different pre-test probabilities for typical, atypical and non-anginal chest pain, but AHA/ACC guidelines use pre-test probabilities of typical chest pain for any chest pain. This most likely explains why AHA/ACC pre-test and post-test probabilities overestimated real probabilities in calibration analysis, and why they tend towards higher sensitivity and NPV. By contrast, ESC probabilities were better calibrated, tended towards higher discriminative capacity and classified more participants at low probability. Thus, AHA/ACC pre- and post-test probabilities might be slightly safer for gatekeeping, but ESC pre- and post-test probabilities rule out perfusion defects in more participants.

Previous works on the ability of CACS to predict PET perfusion defects, mostly in smaller cohorts, showed conflicting results.^5,12,14,25–27^ While several publications reported ≥ 99% NPV from CACS 0 (for SSS ≥ 4 or moderate-to-severe ischemia > 10%),^12,14,25,26^ other observed lower NPV (84%, 91%), but used lower definitions of perfusion defects (SDS ≥ 2, total deficit ≥ 5%).^5,27^ About the combination of CACS with pre-test-probabilities, our favorable results differ from a recent work showing a limited discriminative benefit of adding CACS to pre-test-ESC or pre-test-AHA/ACC.^34^ However, findings from this study cannot be directly compared with ours because an older version of pre-test-AHA/ACC from previous guidelines was used and because patients with CACS 0 were excluded, making the study sample different and limiting the ability of low CACS to rule out CAD. In addition, CAD definition was unusual, as stenosis on ICA if available or cardiac event within 12 months. Previous studies reported that adding CACS to clinical parameters improved CAD prediction, but they also used older versions of pre-test probabilities from previous guidelines with different calibrations, and they did not analyze gatekeeping performance.^34,35^ Another recent work presented a comprehensive model integrating pre-test-ESC, risk factors and CACS to predict CAD, but this model was more complex to use than ours and its sensitivity was only 91.5% for CAD at the 5% cut-off.^36^ About low MFR, the substantial prevalence observed with CACS 0 was also found in previous studies, and is most likely explained by microvascular disease.^11,12,14,37^ Further studies highlighted the value of combining CACS with total ischemic perfusion deficit and MFR to predict CAD.^27,38^ Compared with other imaging modalities, the excellent NPV of CACS 0 for perfusion defects on PET observed in our study was somewhat higher than in older meta-analyses of CACS 0 for SPECT (NPV 93% and 94% for ischemia),^18,24^ and in line with current data on CACS 0 for coronary CT in patients with chest pain (NPV 97% for stenosis ≥ 50%).^20^ Furthermore, in a large cohort of participants with myocardial PET/CT, 0.5% of those with CACS 0 had high-grade CAD needing early revascularization, similar to our prevalence of severe ischemia.^23^ Regarding outcomes, meta-analyses have reported low annual cardiac event rates < 1% with CACS 0, with or without chest pain, either acute or chronic.^18–20^ However, our study adds new insights on the predictive ability of CACS for PET perfusion defects, provides a comparison with the recently updated pre-test-AHA/ACC and pre-test-ESC, evaluates gatekeeping performance, and demonstrates the added value of a simple Bayesian approach to combine pre-test probabilities and CACS into post-test probabilities to improve probability stratification and extend the identification of patients unlikely to have perfusion defects from CAD. These post-test probabilities can be seen as updated pre-test probabilities before further testing. They may be easily estimated from pre-test probabilities and CACS using Figure 3. Moreover, our group recently demonstrated that the excellent NPV of CACS 0 for perfusion defects on PET is maintained across sex and age groups, despite different prevalence of CACS 0 across these groups.^39^ Our results provide a basis to use CACS and post-test probabilities to support decision-making in clinical practice for all sex and age groups.

An improved patient selection through gatekeeping before advanced cardiac imaging may yield multiple benefits. Indeed, cardiac imaging tests generate substantial health costs (estimated from $360 for stress echocardiography to $1850 for PET) and may entail a relevant radiation burden (up to 10-15 mSv with SPECT and 4 mSv with Rubidium-82 PET).^15,16,40^ By comparison, at our center, a low-dose CT for CACS costs about $300, requires 0.2-0.4 mSv of radiation, and is performed faster than any other imaging test. Thus, using CACS or post-test probabilities to identify and discharge patients at low probability of perfusion defects from CAD without further testing may reduce healthcare costs, radiation burden, and waiting times for cardiac imaging. This may prove particularly advantageous given the current downwards trend for positive test findings.^4,28^ Based on our results and previous studies, patients with CACS 0, pre-test-probability ≤ 5%, or post-test probability ≤ 5% may be reassured without further testing. Moreover, our 96% sensitivity with 97% NPV for abnormal perfusion, and 99% sensitivity with > 99% NPV for severe ischemia provide strong support for reassurance. These metrics compare favorably with those of imaging tests vs. CAD by invasive functional flow reserve: PET and magnetic resonance have about 89% sensitivity with 88% NPV, while SPECT has about 73% sensitivity with 70% NPV.^7,8^ With its higher metrics, CACS 0 can be used alone as a gatekeeper. However, combining it with pre-test probabilities into post-test probabilities allows to better quantify the individual probability of perfusion defects due to CAD, integrating age, gender, symptoms and CACS. As shown on Figure 3, even the maximal pre-test-AHA/ACC or pre-test-ESC of 52% would be converted to low post-test probabilities when combined with CACS 0: 11% abnormal perfusion and 3.8% severe ischemia. By contrast, patients with CACS ≥ 2500 or post-test probability ≥ 85% may be ruled in for CAD, given the high specificity and PPV for perfusion defects. However, these probabilities should support, not replace medical judgment, particularly for high-risk groups such as diabetic patients, who may benefit from CAD testing even without symptoms.^41^

This study has some limitations. First, endpoints were perfusion defects in PET, not obstructive CAD in ICA. But systematically performing ICA in patients suspected of CAD would yield an unfavorable risk/benefit ratio and disregard current guidelines.^2,3^ With very high diagnostic performance for CAD, PET is the best proxy for hemodynamically significant CAD on ICA,^7,8^ and an excellent predictor of adverse outcomes.^5,6,10–12^ Thus, predicting PET findings effectively predicts CAD and outcomes. CACS is also known to be an outstanding outcome predictor, alone or combined with PET findings.^5,9,11,12,14,18–23^ Moreover, the good calibration of pre-test-ESC for abnormal perfusion highlighted a good correspondence of our abnormal perfusion with the endpoint used to calibrate pre-test-probabilities: obstructive CAD on coronary CT.^4^ Second, our definitions of abnormal findings in PET differed from other studies. But we used current guidelines to define our primary endpoint,^32^ and chose secondary endpoints associated with worse prognosis and affecting treatment decisions.^10,33^ Third, image assessment was performed on a routine basis with access to patient data. However, CACS is an objective, computer-based measurement, and PET was assessed by the same experts throughout the study. Fourth, the usefulness of ischemia testing and of revascularization for stable CAD have been questioned by the ISCHEMIA trial through a lack of benefit on hard outcomes.^42^ However, high-risk participants with main stem lesions were excluded from this trial, and revascularization improved quality of life, angina, and nonprocedural infarction, as confirmed by a later meta-analysis.^42–44^ Thus, ischemia testing and revascularization remained recommended after ISCHEMIA. Our work also has several strengths: the large prospective database, the detailed patient data including symptoms, allowing for calculation of pre-test probabilities, the comparison with the new pre-test-AHA/ACC and pre-test-ESC, the use of PET as the most accurate non-invasive method to assess myocardial perfusion, and the extensive analysis including a Bayesian approach with a focus on gatekeeping.

In conclusion, CACS is an excellent predictor of myocardial PET findings, with higher discriminative capacity than pre-test-AHA/ACC and pre-test-ESC. CACS 0, pre-test-AHA/ACC ≤ 5% and pre-test-ESC ≤ 5% have very high sensitivity and NPV to rule out abnormal perfusion and severe ischemia, but CACS 0 identifies many more patients with low probability of perfusion defects due to CAD. Pre-test probabilities and CACS can be combined into post-test probabilities to refine the prediction and extend the ability to rule out perfusion defects in more patients, using our Figure 3. Therefore, CACS should be considered as a low-cost, low-radiation, but high-performance gatekeeper test before advanced cardiac imaging. Based on current guidelines and our results, we propose to assess patients with suspected CAD first using a pre-test probability and not to proceed to further testing if it is ≤ 5%. If the pre-test probability is > 5%, CACS should be measured to refine the prediction and perfusion defects may be ruled out if CACS is 0 or post-test probability is ≤ 5%. Only patients with higher values would need advanced cardiac imaging.

## New knowledge gained

CACS alone or combined with pre-test probabilities as post-test probabilities show a better discrimination capacity than pre-test probabilities from AHA/ACC or ESC guidelines for perfusion defects in PET. All of them can rule out such defects with very high negative predictive value: ≥ 96% for abnormal perfusion and ≥ 99% for severe ischemia. But CACS and post-test probabilities rule out perfusion defects in many more participants than pre-test-probabilities (23-37% vs. 2-7%), thus can identify substantially more people for whom PET may not be necessary. Therefore, CACS and post-test probabilities should be considered as low-cost, low-radiation, but high-performance gatekeepers to rule out perfusion defects before advanced imaging tests. Our Figure 3 can support decision-making in clinical routine. This gatekeeping approach using CACS and post-test probabilities may save costs, radiation, and time by identifying patients very unlikely to show perfusion defects due to significant CAD.

### Supplementary Information

Below is the link to the electronic supplementary material.

Supplementary file1 (DOCX 2380 KB)

Supplementary file2 (PPTX 581 KB)

## Abbreviations

AHA/ACC: American Heart Association/American College of Cardiology
CAD: Coronary artery disease
CACS: Coronary artery calcium score
CT: Computed tomography
ESC: European Society of Cardiology
IDI: Integrated discrimination index
MFR: Myocardial flow reserve
NRI: Net reclassification index
PET: Positron emission tomography
SPECT: Single-photon emission computed tomography
