# Supplementary material for: Coronary artery calcium score and pre-test probabilities as gatekeepers to predict and rule out perfusion defects in positron emission tomography
Source: J Nucl Cardiol. 2023 Jul 6;30(6):2559–73. doi: 10.1007/s12350-023-03322-3 (PMC10682222; doi:10.1007/s12350-023-03322-3)
Supplement: Supplementary file 1 — Supplementary file1 (DOCX 2380 KB) [file 12350_2023_3322_MOESM1_ESM.docx]

**ELECTRONIC SUPPLEMENTARY MATERIAL**

**Coronary Artery Calcium Score and Pre-Test Probabilities as Gatekeepers**

**to Predict and Rule Out Perfusion Defects in Positron Emission Tomography**

*Journal of Nuclear Cardiology*

Olivier F Clerc MD^a,b^, Simon M Frey MD^a,b^, Ursina Honegger^b^, Melissa LF Amrein^b^, Federico Caobelli MD^c^, Philip Haaf MD^a,b^, Michael J Zellweger MD^a,b^

^a^ Department of Cardiology, University Hospital Basel, University of Basel, Switzerland

^b^ Cardiovascular Research Institute Basel (CRIB), University Hospital Basel, University of Basel, Switzerland

^c^ Department of Nuclear Medicine, Clinic of Radiology and Nuclear Medicine, University Hospital Basel, University of Basel, Switzerland

**Address for correspondence:**

Michael J. Zellweger, MD

Professor of Cardiology

Department of Cardiology

University Hospital Basel

Petersgraben 4, CH-4031 Basel, Switzerland

michael.zellweger@usb.ch

Phone: +41 61 265 54 73

Fax: +41 61 265 45 98

**ELECTRONIC SUPPLEMENTARY MATERIAL**

**Part A**

**Additional Analyses with the New Pre-Test Probabilities AHA/ACC and ESC**

**Abbreviation List** Page 3

**Figure A1 Flowchart for Participant Inclusion** Page 4

**Figure A1 Distribution of Probabilities** Page 5

**Figure A2 Calibration Plots** Page 6

**Figure A3 Discrimination Capacity** Page 7

**Table A1a Multivariable Analysis with Detailed Symptoms** Page 10

**Table A1b Multivariable Analysis with Pre-Test Probability AHA/ACC** Page 11

**Table A1c Multivariable Analysis with Pre-Test Probability ESC** Page 12

**Table A2 Interval Likelihood Ratios by CACS Thresholds** Page 13

**Table Group A3 Reclassification Analyses** Page 14

**Abbreviation List**

AHA/ACC American Heart Association/American College of Cardiology.

CAD Coronary artery disease.

ESC European Society of Cardiology.

IDI Integrated discrimination improvement

ILR Interval likelihood ratio

MFR Myocardial flow reserve

NRI Net reclassification improvement

PET Position emission tomography.

**Figure A1 Flowchart for Participant Inclusion**


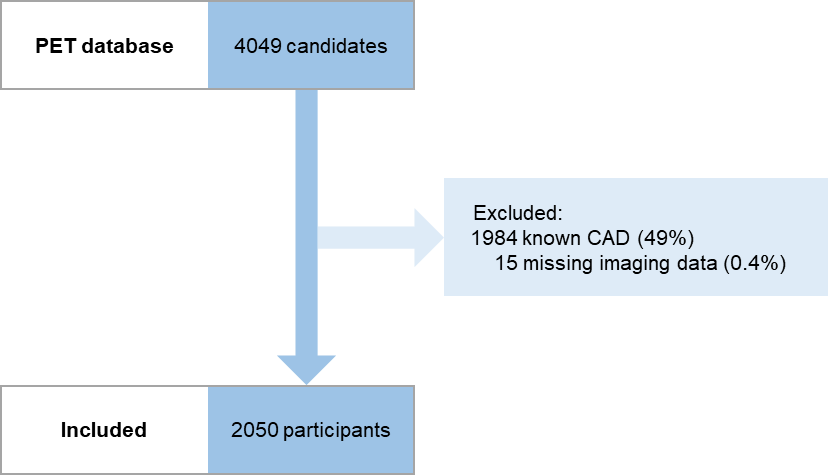


This flowchart presents the total amount of study candidates in our prospective PET database, the candidates excluded with the reasons, and the participants included in the present study.

**Figure A2 Distribution of Probabilities**

**
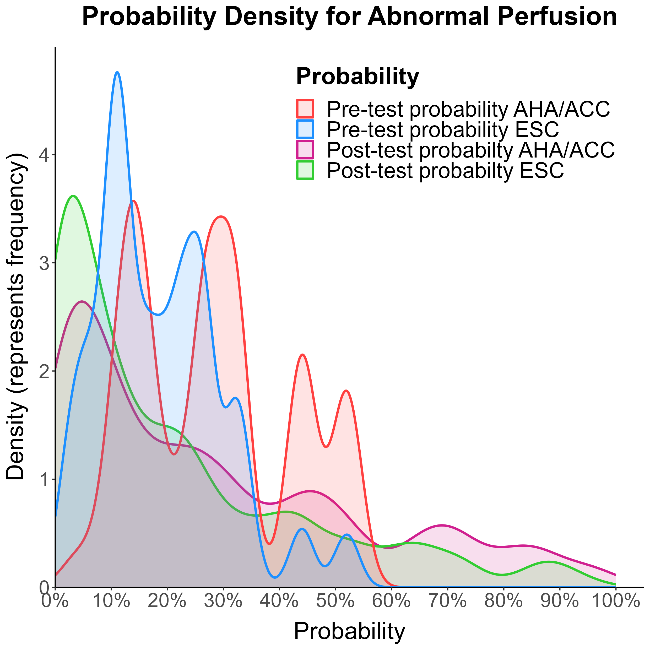
** **
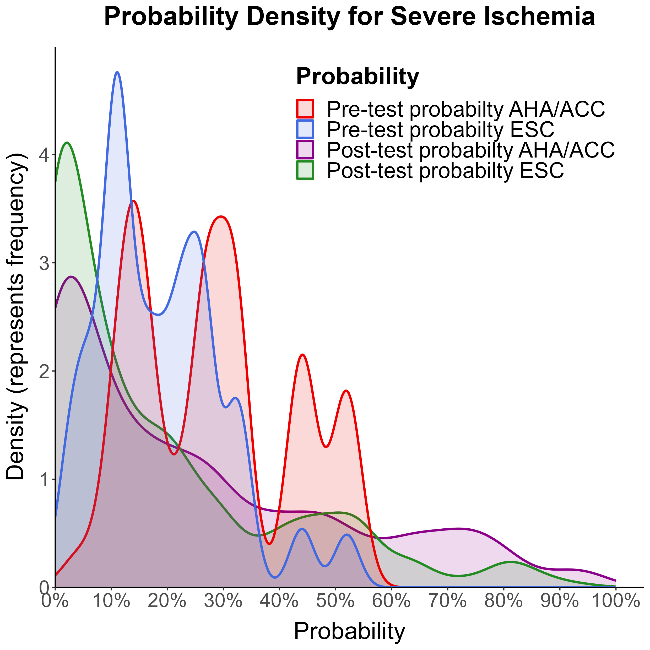
**

These graphs present frequencies of each probability for abnormal perfusion and severe ischemia in density form (smooth equivalent to histogram). They show a clear shift towards lower predicted probabilities with post-test probabilities, more with ESC (high green spikes at low probabilities on each graph) than with AHA/ACC (purple spikes).

**Figure A3 Calibration Plots**


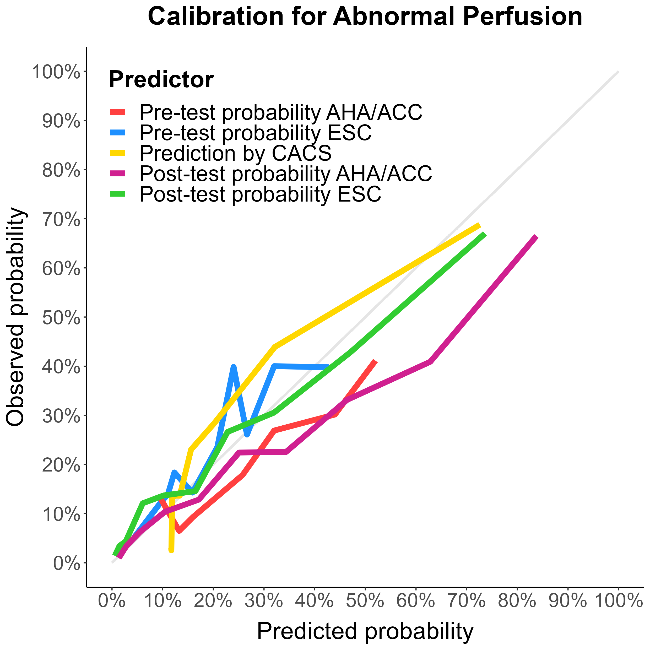

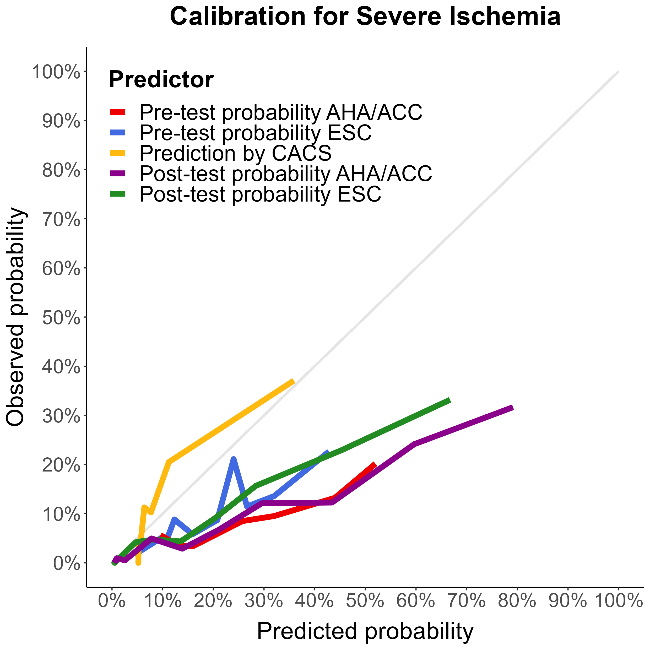


Calibration plots compare predicted versus observed probabilities of abnormal perfusion or severe ischemia, with the diagonal line as a reference for equal predicted and observed probabilities. For abnormal perfusion, ESC probabilities were quite well calibrated, but AHA/ACC predicted probabilities somewhat overestimated the observed probabilities. Because severe ischemia is a subgroup of patients with abnormal perfusion, it was generally overestimated, more by AHA/ACC than ESC probabilities. CACS needed transformation to probabilities using logistic regression.

**Figure A4 Discrimination Capacity**


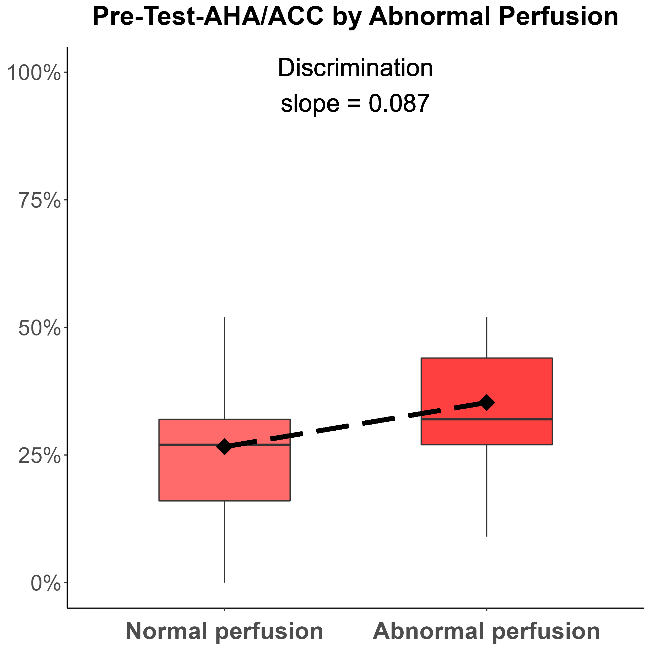

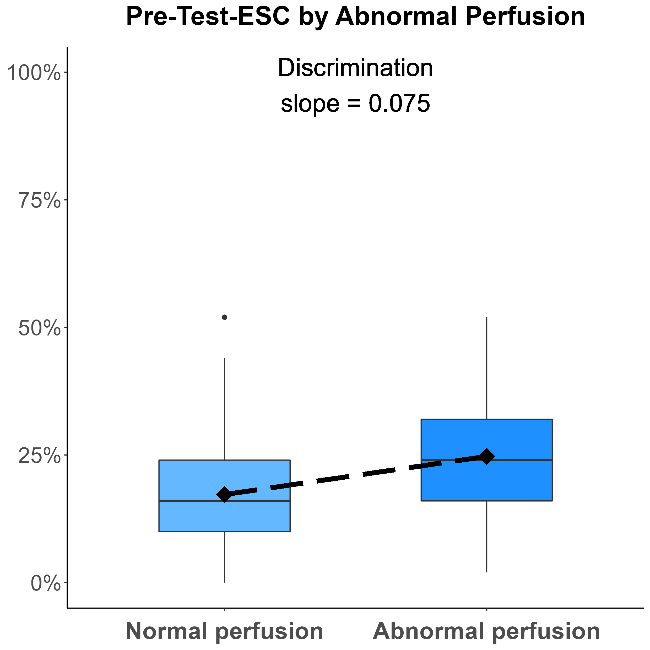


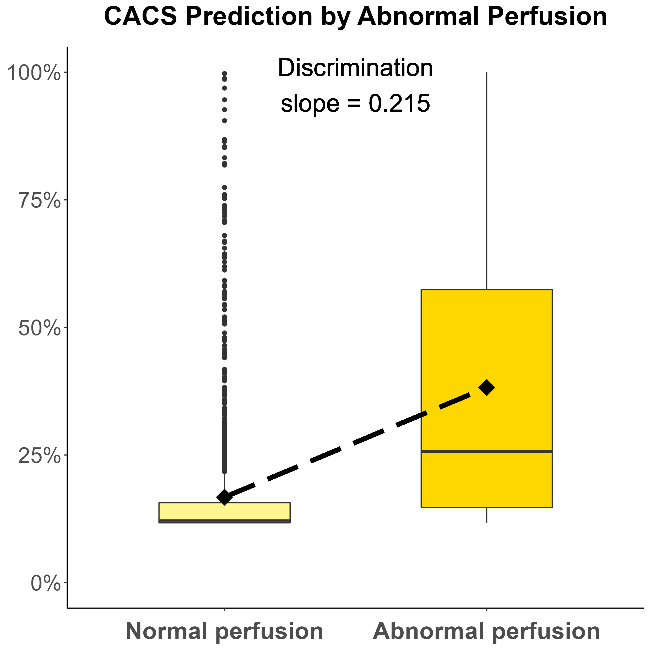


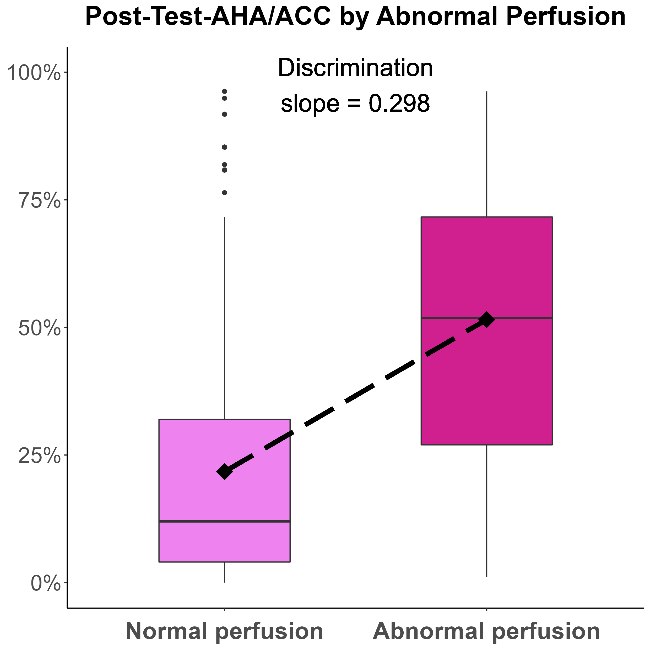

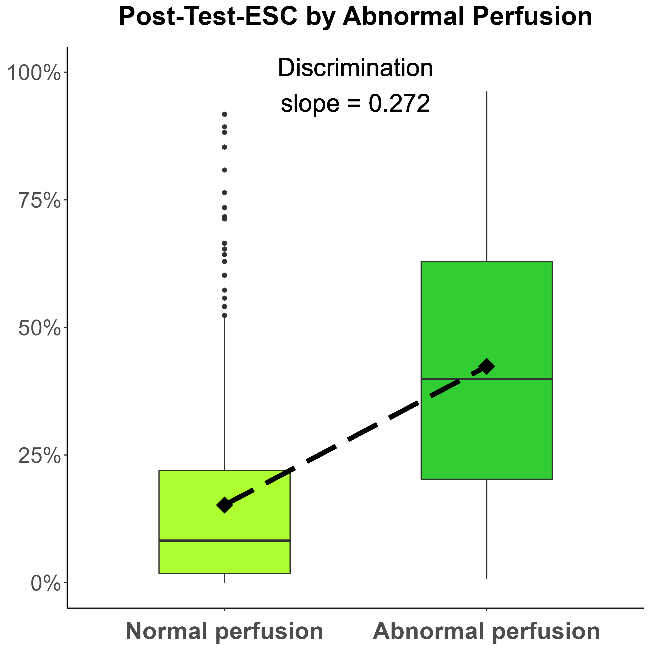


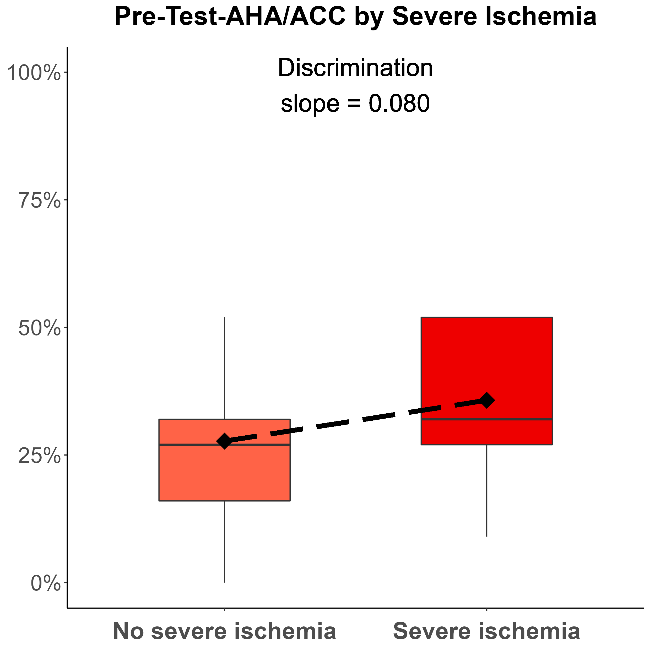

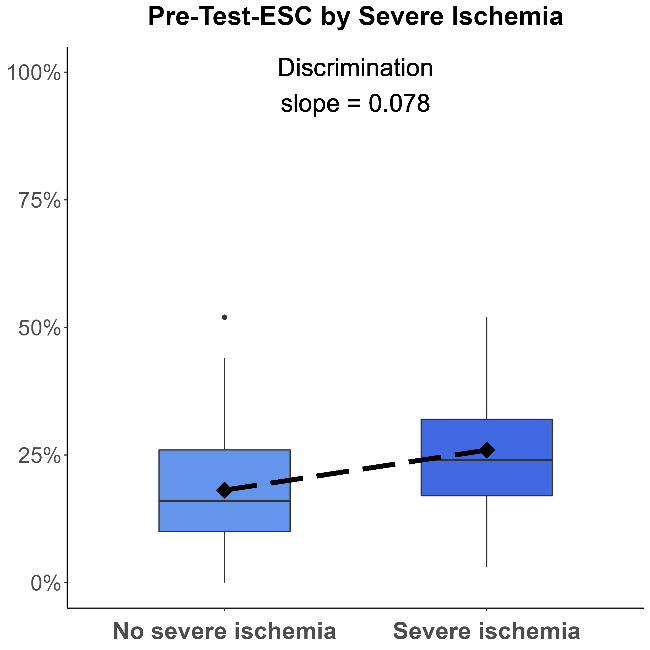

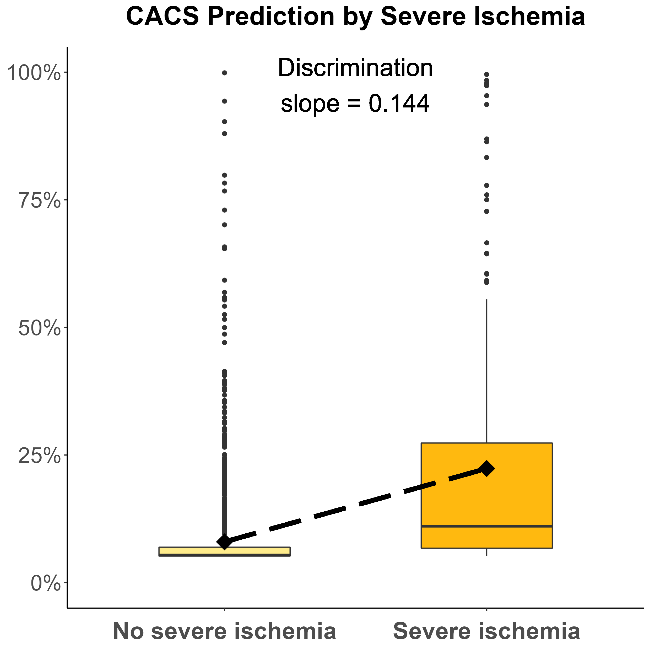


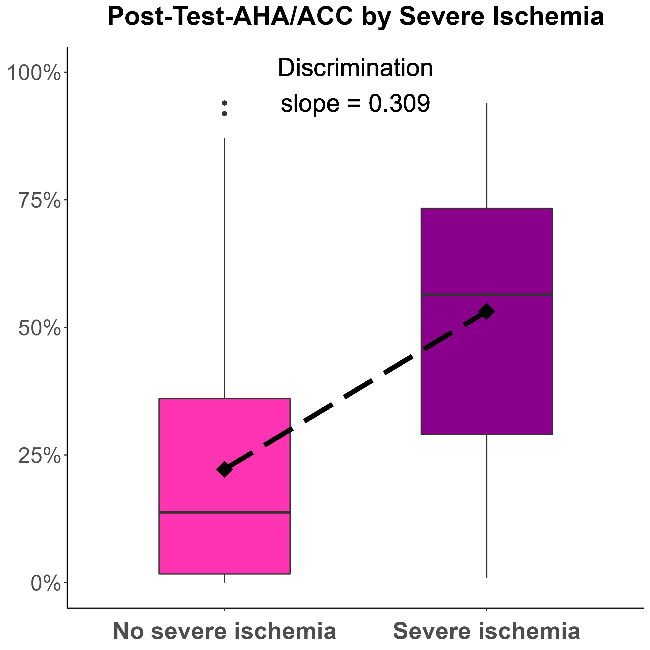

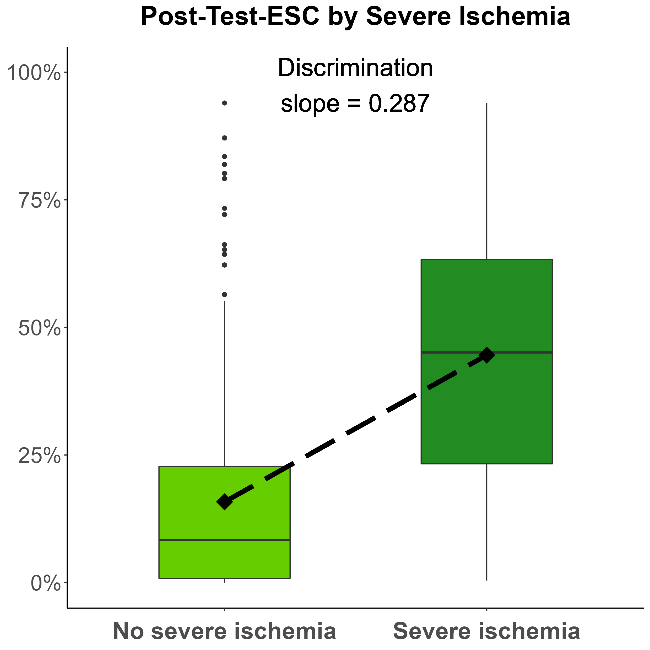


Each box plot presents the values of a predictor for patients without versus with the endpoint. CACS needed transformation to probabilities using logistic regression. Means are shown as black diamonds, and their differences are shown as dashed lines (discrimination slopes). Steeper slopes indicate better endpoint discrimination. The integrated discrimination improvement (IDI) is the difference between discrimination slopes.

For abnormal perfusion, we found the following IDI: pre-test-AHA/ACC versus pre-test-ESC -0.012 (-0.023–-0.001, p=0.036), pre-test-AHA/ACC versus CACS 0.128 (0.101–0.155, p<0.001), pre-test-ESC versus CACS 0.140 (0.112–0.168, p<0.001), CACS versus post-test-AHA/ACC 0.083 (0.066–0.101, p<0.001), CACS versus post-test-ESC 0.057 (0.041–0.073, p<0.001), pre-test-AHA/ACC versus post-test-AHA/ACC 0.211 (0.190–0.233, p<0.001), pre-test-ESC versus post-test-ESC 0.197 (0.175–0.219, p<0.001), and post-test-AHA/ACC versus post-test-ESC -0.026 (-0.037–-0.016, p<0.001).

For severe ischemia, we found the following IDI: pre-test- AHA/ACC versus pre-test-ESC -0.002 (-0.018–0.015, p=0.854), pre-test-AHA/ACC versus CACS 0.064 (0.031–0.097, p<0.001), pre-test-ESC versus CACS 0.065 (0.029–0.102, p<0.001), CACS versus post-test-AHA/ACC 0.166 (0.137–0.195, p<0.001), CACS versus post-test-ESC 0.144 (0.118–0.170, p<0.001), pre-test-AHA/ACC versus post-test-AHA/ACC 0.229 (0.201–0.259, p<0.001), pre-test-ESC versus post-test-ESC 0.209 (0.178–0.240, p<0.001), and post-test-AHA/ACC versus post-test-ACC -0.022 (-0.037–-0.007, p=0.004).

Overall, this analysis highlights the increased discrimination capacity from post-test probabilities versus pre-test probabilities. Comparisons of AHA/ACC versus ESC probabilities are affected by the different calibration. AHA/ACC probabilities tend to overestimate endpoint prevalence, which increases AHA/ACC discrimination slopes.

**Table A1a Multivariable Analysis with Detailed Symptoms**

| Predictors | Abnormal perfusion | Severe ischemia | Low MFR |
| --- | --- | --- | --- |
| Age (by 10 years) | 1.26 (1.10–1.44) p=0.003 | 1.15 (0.96–1.39) p=0.203 | 1.38 (1.21–1.57) p<0.001 |
| Body mass index | 0.99 (0.97–1.01) p=0.533 | 0.97 (0.94–1.00) p=0.128 | 0.98 (0.96–1.00) p=0.087 |
| Male gender | 2.51 (1.91–3.32) p<0.001 | 1.84 (1.26–2.71) p=0.006 | 0.64 (0.50–0.83) p=0.002 |
| Insulin-dependent diabetes mellitus | 1.10 (0.68–1.74) p=0.689 | 1.64 (0.91–2.84) p=0.145 | 2.51 (1.65–3.79) p<0.001 |
| Non-insulin-dependent diabetes mellitus | 1.10 (0.79–1.52) p=0.602 | 1.46 (0.95–2.21) p=0.145 | 1.42 (1.03–1.93) p=0.057 |
| Hypercholesterolemia | 1.19 (0.92–1.53) p=0.394 | 1.89 (1.33–2.70) p=0.001 | 0.98 (0.77–1.25) p=0.919 |
| Arterial hypertension | 1.17 (0.88–1.57) p=0.525 | 1.13 (0.75–1.71) p=0.652 | 1.37 (1.04–1.83) p=0.057 |
| Active smoking | 1.16 (0.84–1.61) p=0.533 | 1.84 (1.21–2.79) p=0.010 | 0.98 (0.72–1.34) p=0.919 |
| Previous smoking | 1.10 (0.82–1.46) p=0.602 | 1.02 (0.67–1.53) p=0.930 | 0.74 (0.55–0.99) p=0.068 |
| Family history of CAD | 1.13 (0.84–1.51) p=0.549 | 1.06 (0.70–1.57) p=0.840 | 0.57 (0.41–0.77) p=0.001 |
| Typical angina pectoris | 1.83 (1.30–2.57) p=0.002 | 3.91 (2.50–6.17) p<0.001 | 1.20 (0.85–1.68) p=0.362 |
| Atypical angina pectoris | 0.88 (0.63–1.24) p=0.594 | 1.33 (0.82–2.14) p=0.341 | 1.09 (0.79–1.49) p=0.701 |
| Dyspnea | 1.44 (1.03–2.02) p=0.086 | 1.19 (0.71–1.96) p=0.637 | 1.31 (0.94–1.81) p=0.148 |
| CACS (by 100 units) | 1.14 (1.11–1.16) p<0.001 | 1.10 (1.08–1.12) p<0.001 | 1.07 (1.06–1.09) p<0.001 |

Results are presented as odds ratios with 95% confidence intervals from multivariable logistic regressions. P-values are adjusted for multiple comparisons using the Benjamini-Hochberg sequential procedure, which shows the false discovery rate.

**Table A1b Multivariable Analysis with Pre-Test Probability AHA/ACC**

| Predictors | Abnormal perfusion | Severe ischemia | Low MFR |
| --- | --- | --- | --- |
| Pre-test-AHA/ACC (%) | 1.03 (1.02–1.04) p<0.001 | 1.02 (1.01–1.03) p=0.003 | 0.99 (0.98–1.00) p=0.208 |
| Body mass index | 0.99 (0.97–1.01) p=0.453 | 0.97 (0.94–1.00) p=0.132 | 0.97 (0.94–0.99) p=0.003 |
| Insulin-dependent diabetes mellitus | 1.09 (0.68–1.71) p=0.729 | 1.60 (0.90–2.73) p=0.137 | 2.29 (1.51–3.43) p<0.001 |
| Non-insulin-dependent diabetes mellitus | 1.15 (0.83–1.59) p=0.519 | 1.42 (0.93–2.14) p=0.137 | 1.40 (1.02–1.90) p=0.048 |
| Hypercholesterolemia | 1.17 (0.91–1.50) p=0.453 | 1.91 (1.36–2.71) p<0.001 | 0.99 (0.78–1.26) p=0.941 |
| Arterial hypertension | 1.21 (0.91–1.61) p=0.453 | 1.15 (0.77–1.72) p=0.612 | 1.57 (1.19–2.08) p=0.003 |
| Active smoking | 1.13 (0.83–1.54) p=0.519 | 1.65 (1.11–2.44) p=0.028 | 0.75 (0.55–1.01) p=0.070 |
| Previous smoking | 1.17 (0.88–1.55) p=0.453 | 1.04 (0.69–1.55) p=0.851 | 0.69 (0.52–0.92) p=0.021 |
| Family history of CAD | 1.11 (0.83–1.47) p=0.530 | 1.12 (0.75–1.64) p=0.634 | 0.52 (0.38–0.70) p<0.001 |
| CACS (by 100 units) | 1.15 (1.12–1.17) p<0.001 | 1.09 (1.07–1.11) p<0.001 | 1.08 (1.06–1.10) p<0.001 |

Results are presented as odds ratios with 95% confidence intervals from multivariable logistic regressions. P-values are adjusted for multiple comparisons using the Benjamini-Hochberg sequential procedure, which shows the false discovery rate.

**Table A1c Multivariable Analysis with Pre-Test Probability ESC**

| Predictors | Abnormal perfusion | Severe ischemia | Low MFR |
| --- | --- | --- | --- |
| Pre-test-ESC (%) | 1.04 (1.03–1.05) p<0.001 | 1.04 (1.03–1.06) p<0.001 | 1.00 (0.99–1.02) p=0.413 |
| Body mass index | 0.99 (0.96–1.01) p=0.413 | 0.97 (0.94–1.00) p=0.111 | 0.97 (0.95–0.99) p=0.004 |
| Insulin-dependent diabetes mellitus | 1.09 (0.68–1.73) p=0.709 | 1.67 (0.94–2.86) p=0.114 | 2.30 (1.52–3.44) p<0.001 |
| Non-insulin-dependent diabetes mellitus | 1.15 (0.82–1.58) p=0.503 | 1.45 (0.94–2.19) p=0.114 | 1.38 (1.01–1.88) p=0.054 |
| Hypercholesterolemia | 1.15 (0.90–1.48) p=0.413 | 1.91 (1.35–2.72) p<0.001 | 0.99 (0.78–1.26) p=0.956 |
| Arterial hypertension | 1.20 (0.90–1.60) p=0.413 | 1.11 (0.75–1.67) p=0.683 | 1.54 (1.17–2.05) p=0.004 |
| Active smoking | 1.21 (0.89–1.65) p=0.413 | 1.80 (1.21–2.68) p=0.008 | 0.76 (0.56–1.01) p=0.080 |
| Previous smoking | 1.15 (0.87–1.53) p=0.450 | 0.99 (0.66–1.48) p=0.962 | 0.67 (0.50–0.89) p=0.010 |
| Family history of CAD | 1.11 (0.83–1.48) p=0.518 | 1.13 (0.76–1.66) p=0.655 | 0.53 (0.39–0.72) p<0.001 |
| CACS (by 100 units) | 1.15 (1.12–1.17) p<0.001 | 1.09 (1.07–1.11) p<0.001 | 1.07 (1.06–1.09) p<0.001 |

Results are presented as odds ratios with 95% confidence intervals from multivariable logistic regressions. P-values are adjusted for multiple comparisons using the Benjamini-Hochberg sequential procedure, which shows the false discovery rate.

**Table A2 Interval Likelihood Ratios by CACS Thresholds**

| CACS | Prevalence | Abnormal perfusion | Severe Ischemia | Low MFR |
| --- | --- | --- | --- | --- |
| 0 | 26.1% | 0.114 (0.070–0.185) | 0.036 (0.009–0.145) | 0.48 (0.38–0.62) |
| 1–9 | 9.6% | 0.28 (0.17–0.48) | 0.20 (0.08–0.54) | 0.44 (0.28–0.70) |
| 10–99 | 20.2% | 0.67 (0.53–0.86) | 0.52 (0.34–0.79) | 0.76 (0.59–0.96) |
| 100–399 | 19.5% | 1.00 (0.81–1.24) | 0.82 (0.59–1.15) | 1.02 (0.82–1.27) |
| 400–999 | 12.9% | 2.29 (1.83–2.86) | 2.39 (1.83–3.11) | 1.52 (1.19–1.96) |
| 1000–2499 | 9.2% | 5.37 (4.10–7.03) | 3.50 (2.63–4.66) | 2.84 (2.17–3.72) |
| ≥2500 | 2.5% | 23.73 (10.78–52.25) | 14.37 (8.43–24.5) | 7.66 (4.37–13.42) |

Interval likelihood ratios (ILR) quantify the predictive performance of each CACS category. ILR are the ratio of the probability of having the CACS level for patients with the perfusion endpoint divided by the probability of having the CACS level for patients without the perfusion endpoint. ILR ≤0.1 are strong negative predictors, while ILR ≥10 are strong positive predictors. Confidence intervals were calculated with the Katz log method.

**Table Group A3 Reclassification Analyses**

Each table presents the classification of patients from a predictor in rows to another predictor in columns. The upper part of each table shows patients with the perfusion endpoint and the lower part patients without the perfusion endpoint. Green cells indicate “correct” reclassifications by the second predictor in columns (patients with endpoint to higher probability, patients without endpoint to lower probability). Red cells indicate “incorrect” reclassifications by the second predictor in columns (patients with endpoint to lower probability, patients without endpoint to higher probability). Yellow cells indicate patients with the same classification by both predictors. The net reclassification improvement (NRI) sums up the proportions of “correct” minus “incorrect” reclassifications.

**Table A3a Pre-Test Probability AHA/ACC versus Pre-Test Probability ESC for Abnormal Perfusion**

| **Abnormal perfusion:** NRI 0.154 (0.114–0.193), p<0.001 | | | | | | |
| --- | --- | --- | --- | --- | --- | --- |
| **Abnormal**  **perfusion** | | **Pre-test-ESC** | | | | |
|  |  | **0–5%** | **6–15%** | **16–85%** | **>85%** | **Total** |
| **Pre-test-AHA/ACC** | **0–5%** | 0 | 0 | 0 | 0 | 0 |
|  | **6–15%** | 2 | 41 | 0 | 0 | 43 |
|  | **16–85%** | 1 | 59 | 334 | 0 | 394 |
|  | **>85%** | 0 | 0 | 0 | 0 | 0 |
|  | **Total** | 3 | 100 | 334 | 0 | 437 |
| **Normal**  **perfusion** | | **Pre-test-ESC** | | | | |
|  |  | **0–5%** | **6–15%** | **16–85%** | **>85%** | **Total** |
| **Pre-test-AHA/ACC** | **0–5%** | 41 | 0 | 0 | 0 | 41 |
|  | **6–15%** | 75 | 285 | 0 | 0 | 360 |
|  | **16–85%** | 27 | 375 | 810 | 0 | 1212 |
|  | **>85%** | 0 | 0 | 0 | 0 | 0 |
|  | **Total** | 143 | 660 | 810 | 0 | 1613 |

With pre-test AHA/ACC as a reference, using pre-test ESC in patients with abnormal perfusion (upper table) moved 0 patient correctly to the right to higher probabilities and 62 (14.2%) incorrectly to the left to lower probabilities. In patients with normal perfusion (lower table), it moved 477 patients (29.6%) correctly to the left to lower probabilities and 0 incorrectly to the right to higher probabilities. The NRI was 0.154 (0.114–0.193, p<0.001) in favor of pre-test-ESC, due to the numerous correct reclassifications to lower probability categories.

The different calibration of AHA/ACC and ESC probabilities affects these results, as AHA/ACC probabilities tend to overestimate the probability of abnormal perfusion.

**Table A3b Pre-Test Probability AHA/ACC versus Post-Test Probability AHA/ACC for Abnormal Perfusion**

| **Abnormal perfusion:** NRI 0.543 (0.487–0.599), p<0.001 | | | | | | |
| --- | --- | --- | --- | --- | --- | --- |
| **Abnormal**  **perfusion** | | **Post-test-AHA/ACC** (pre-test-AHA/ACC combined with CACS) | | | | |
|  |  | **0–5%** | **6–15%** | **16–85%** | **>85%** | **Total** |
| **Pre-test-AHA/ACC** | **0–5%** | 0 | 0 | 0 | 0 | 0 |
|  | **6–15%** | 3 | 22 | 18 | 0 | 43 |
|  | **16–85%** | 13 | 16 | 287 | 78 | 394 |
|  | **>85%** | 0 | 0 | 0 | 0 | 0 |
|  | **Total** | 16 | 38 | 305 | 78 | 437 |
| **Normal**  **perfusion** | | **Post-test-AHA/ACC** (pre-test-AHA/ACC combined with CACS) | | | | |
|  |  | **0–5%** | **6–15%** | **16–85%** | **>85%** | **Total** |
| **Pre-test-AHA/ACC** | **0–5%** | 41 | 0 | 0 | 0 | 41 |
|  | **6–15%** | 232 | 102 | 26 | 0 | 360 |
|  | **16–85%** | 262 | 202 | 717 | 31 | 1212 |
|  | **>85%** | 0 | 0 | 0 | 0 | 0 |
|  | **Total** | 535 | 304 | 743 | 31 | 1613 |

With pre-test-AHA/ACC as a reference, using post-test-AHA/ACC in patients with abnormal perfusion (upper table) moved 96 patients (22.0%) correctly to the right to higher probabilities and 32 (7.3%) incorrectly to the left to lower probabilities. In patients with normal perfusion (lower table), it moved 696 patients (43.1%) correctly to the left to lower probabilities and 57 (3.5%) incorrectly to the right to higher probabilities. The NRI was 0.543 (0.487–0.599, p<0.001) in favor of post-test-AHA/ACC, due to the numerous correct reclassifications to lower probability categories.

**Table A3c Pre-Test Probability ESC versus Post-Test Probability ESC for Abnormal Perfusion**

| **Abnormal perfusion:** NRI 0.501 (0.443–0.558), p<0.001 | | | | | | |
| --- | --- | --- | --- | --- | --- | --- |
| **Abnormal perfusion** | | **Post-test-ESC (pre-test-ESC with CACS)** | | | | |
|  |  | **0–5%** | **6–15%** | **16–85%** | **>85%** | **Total** |
| **Pre-test-ESC** | **0–5%** | 1 | 1 | 1 | 0 | 3 |
|  | **6–15%** | 18 | 43 | 39 | 0 | 100 |
|  | **16–85%** | 8 | 13 | 259 | 54 | 334 |
|  | **>85%** | 0 | 0 | 0 | 0 | 0 |
|  | **Total** | 27 | 57 | 299 | 54 | 437 |
| **Normal perfusion** | | **Post-test-ESC (pre-test-ESC with CACS)** | | | | |
|  |  | **0–5%** | **6–15%** | **16–85%** | **>85%** | **Total** |
| **Pre-test-ESC** | **0–5%** | 139 | 4 | 0 | 0 | 143 |
|  | **6–15%** | 381 | 222 | 57 | 0 | 660 |
|  | **16–85%** | 140 | 150 | 511 | 9 | 810 |
|  | **>85%** | 0 | 0 | 0 | 0 | 0 |
|  | **Total** | 660 | 376 | 568 | 9 | 1613 |

With pre-test-ESC as a reference, using post-test-ESC in patients with abnormal perfusion (upper table) moved 95 patients (21.7%) correctly to the right to higher probabilities and 39 (8.9%) incorrectly to the left to lower probabilities. In patients with normal perfusion (lower table), it moved 671 patients (41.6%) correctly to the left to lower probabilities and 70 (4.3%) incorrectly to the right to higher probabilities. The NRI was 0.501 (0.443–0.558, p<0.001) in favor of post-test-ESC, due to the numerous correct reclassifications to lower probability categories.

**Table A3d Post-Test Probability AHA/ACC versus Post-Test Probability ESC for Abnormal Perfusion**

| **Abnormal perfusion:** NRI 0.059 (0.020–0.098), p=0.003 | | | | | | |
| --- | --- | --- | --- | --- | --- | --- |
| **Abnormal**  **perfusion** | | **Post-test-ESC** | | | | |
|  |  | **0–5%** | **6–15%** | **16–85%** | **>85%** | **Total** |
| **Post-test -AHA/ACC** | **0–5%** | 16 | 0 | 0 | 0 | 16 |
|  | **6–15%** | 11 | 27 | 0 | 0 | 38 |
|  | **16–85%** | 0 | 30 | 275 | 0 | 305 |
|  | **>85%** | 0 | 0 | 24 | 54 | 78 |
|  | **Total** | 27 | 57 | 299 | 54 | 437 |
| **Normal**  **perfusion** | | **Post-test-ESC** | | | | |
|  |  | **0–5%** | **6–15%** | **16–85%** | **>85%** | **Total** |
| **Post-test-AHA/ACC** | **0–5%** | 535 | 0 | 0 | 0 | 535 |
|  | **6–15%** | 116 | 188 | 0 | 0 | 304 |
|  | **16–85%** | 9 | 188 | 546 | 0 | 743 |
|  | **>85%** | 0 | 0 | 22 | 9 | 31 |
|  | **Total** | 660 | 376 | 568 | 9 | 1613 |

With post-test-AHA/ACC as a reference, using post-test-ESC in patients with abnormal perfusion (upper table) moved 0 patient correctly to the right to higher probabilities and 65 (14.9%) incorrectly to the left to lower probabilities. In patients with normal perfusion (lower table), it moved 335 patients (20.8%) correctly to the left to lower probabilities and 0 incorrectly to the right to higher probabilities. The NRI was 0.059 (0.020–0.098, p=0.003) in favor of post-test-ESC, due to the numerous correct reclassifications to lower probability categories.

The different calibration of AHA/ACC and ESC probabilities affects these results, as AHA/ACC probabilities tend to overestimate the probability of abnormal perfusion.

**Table A3e Pre-Test Probability AHA/ACC versus Pre-Test Probability ESC for Severe Ischemia**

| **Severe ischemia:** NRI 0.163 (0.113–0.213), p<0.001 | | | | | | |
| --- | --- | --- | --- | --- | --- | --- |
| **Severe**  **ischemia** | | **Pre-test-ESC** | | | | |
|  |  | **0–5%** | **6–15%** | **16–85%** | **>85%** | **Total** |
| **Pre-test-AHA/ACC** | **0–5%** | 0 | 0 | 0 | 0 | 0 |
|  | **6–15%** | 1 | 19 | 0 | 0 | 20 |
|  | **16–85%** | 0 | 21 | 150 | 0 | 171 |
|  | **>85%** | 0 | 0 | 0 | 0 | 0 |
|  | **Total** | 1 | 40 | 150 | 0 | 191 |
| **No severe**  **ischemia** | | **Pre-test-ESC** | | | | |
|  |  | **0–5%** | **6–15%** | **16–85%** | **>85%** | **Total** |
| **Pre-test-AHA/ACC** | **0–5%** | 41 | 0 | 0 | 0 | 41 |
|  | **6–15%** | 76 | 307 | 0 | 0 | 383 |
|  | **16–85%** | 28 | 413 | 994 | 0 | 1435 |
|  | **>85%** | 0 | 0 | 0 | 0 | 0 |
|  | **Total** | 145 | 720 | 994 | 0 | 1859 |

With pre-test-AHA/ACC as a reference, using pre-test-ESC in patients with severe ischemia (upper table) moved 0 patient correctly to the right to higher probabilities and 22 (11.5%) incorrectly to the left to lower probabilities. In patients without severe ischemia (lower table), it moved 517 patients (27.8%) correctly to the left to lower probabilities and 0 incorrectly to the right to higher probabilities. The NRI was 0.163 (0.113–0.213, p<0.001) in favor of pre-test-ESC, due to the numerous correct reclassifications to lower probability categories.

The different calibration of AHA/ACC and ESC probabilities affects these results, as AHA/ACC probabilities tend to overestimate the probability of severe ischemia more than ESC probabilities.

**Table A3f Pre-Test Probability AHA/ACC versus Post-Test Probability AHA/ACC for Severe Ischemia**

| **Severe Ischemia:** NRI 0.540 (0.467–0.613), p<0.001 | | | | | | |
| --- | --- | --- | --- | --- | --- | --- |
| **Severe**  **ischemia** | | **Post-test-AHA/ACC (pre-test-AHA/ACC with CACS)** | | | | |
|  |  | **0–5%** | **6–15%** | **16–85%** | **>85%** | **Total** |
| **Pre-test-AHA/ACC** | **0–5%** | 0 | 0 | 0 | 0 | 0 |
|  | **6–15%** | 1 | 8 | 11 | 0 | 20 |
|  | **16–85%** | 3 | 7 | 134 | 27 | 171 |
|  | **>85%** | 0 | 0 | 0 | 0 | 0 |
|  | **Total** | 4 | 15 | 145 | 27 | 191 |
| **No severe**  **ischemia** | | **Post-test-AHA/ACC (pre-test-AHA/ACC with CACS)** | | | | |
|  |  | **0–5%** | **6–15%** | **16–85%** | **>85%** | **Total** |
| **Pre-test-AHA/ACC** | **0–5%** | 41 | 0 | 0 | 0 | 41 |
|  | **6–15%** | 238 | 112 | 33 | 0 | 383 |
|  | **16–85%** | 339 | 213 | 867 | 16 | 1435 |
|  | **>85%** | 0 | 0 | 0 | 0 | 0 |
|  | **Total** | 618 | 325 | 900 | 16 | 1859 |

With pre-test-AHA/ACC as a reference, using post-test-AHA/ACC in patients with severe ischemia (upper table) moved 38 patients (19.9%) correctly to the right to higher probabilities and 11 (5.8%) incorrectly to the left to lower probabilities. In patients without severe ischemia (lower table), it moved 790 patients (42.5%) correctly to the left to lower probabilities and 49 (2.6%) incorrectly to the right to higher probabilities. The NRI was 0.540 (0.467–0.613, p<0.001) in favor of post-test-AHA/ACC, due to the numerous correct reclassifications to lower probability categories.

**Table A3g Pre-Test Probability ESC versus Post-Test Probability ESC for Severe Ischemia**

| **Severe ischemia:** NRI 0.466 (0.395–0.537), p<0.001 | | | | | | |
| --- | --- | --- | --- | --- | --- | --- |
| **Severe**  **ischemia** | | **Post-test-ESC (pre-test-ESC with CACS)** | | | | |
|  |  | **0–5%** | **6–15%** | **16–85%** | **>85%** | **Total** |
| **Pre-test-ESC** | **0–5%** | 0 | 1 | 0 | 0 | 1 |
|  | **6–15%** | 6 | 15 | 19 | 0 | 40 |
|  | **16–85%** | 2 | 5 | 133 | 10 | 150 |
|  | **>85%** | 0 | 0 | 0 | 0 | 0 |
|  | **Total** | 8 | 21 | 152 | 10 | 191 |
| **No severe**  **ischemia** | | **Post-test-ESC (pre-test-ESC with CACS)** | | | | |
|  |  | **0–5%** | **6–15%** | **16–85%** | **>85%** | **Total** |
| **Pre-test-ESC** | **0–5%** | 140 | 4 | 1 | 0 | 145 |
|  | **6–15%** | 414 | 229 | 77 | 0 | 720 |
|  | **16–85%** | 190 | 185 | 613 | 6 | 994 |
|  | **>85%** | 0 | 0 | 0 | 0 | 0 |
|  | **Total** | 744 | 418 | 691 | 6 | 1859 |

With pre-test-ESC as a reference, using post-test-ESC in patients with severe ischemia (upper table) moved 30 patients (15.7%) correctly to the right to higher probabilities and 13 (6.8%) incorrectly to the left to lower probabilities. In patients without severe ischemia (lower table), it moved 789 patients (42.4%) correctly to the left to lower probabilities and 88 (4.7%) incorrectly to the right to higher probabilities. The NRI was 0.466 (0.395–0.537, p<0.001) in favor of post-test-ESC, due to the numerous correct reclassifications to lower probability categories.

**Table A3h Post-Test Probability AHA/ACC versus Post-Test Probability ESC for Severe Ischemia**

| **Severe ischemia:** NRI 0.028 (-0.028–0.083), p=0.327 | | | | | | |
| --- | --- | --- | --- | --- | --- | --- |
| **Severe**  **ischemia** | | **Post-test-ESC** | | | | |
|  |  | **0–5%** | **6–15%** | **16–85%** | **>85%** | **Total** |
| **Post-test-AHA/ACC** | **0–5%** | 4 | 0 | 0 | 0 | 4 |
|  | **6–15%** | 4 | 11 | 0 | 0 | 15 |
|  | **16–85%** | 0 | 10 | 135 | 0 | 145 |
|  | **>85%** | 0 | 0 | 17 | 10 | 27 |
|  | **Total** | 8 | 21 | 152 | 10 | 191 |
| **No severe**  **ischemia** | | **Post-test-ESC** | | | | |
|  |  | **0–5%** | **6–15%** | **16–85%** | **>85%** | **Total** |
| **Post-test-AHA/ACC** | **0–5%** | 618 | 0 | 0 | 0 | 618 |
|  | **6–15%** | 124 | 201 | 0 | 0 | 325 |
|  | **16–85%** | 2 | 217 | 681 | 0 | 900 |
|  | **>85%** | 0 | 0 | 10 | 6 | 16 |
|  | **Total** | 744 | 418 | 691 | 6 | 1859 |

With post-test-AHA/ACC as a reference, using post-test-ESC in patients with severe ischemia (upper table) moved 0 patient correctly to the right to higher probabilities and 31 (16.2%) incorrectly to the left to lower probabilities. In patients without severe ischemia (lower table), it moved 352 patients (19.0%) correctly to the left to lower probabilities and 0 incorrectly to the right to higher probabilities. The NRI was 0.028 (-0.028–0.083, p=0.327), due to the balance between percentages of correct and incorrect reclassifications.

The different calibration of AHA/ACC and ESC probabilities affects these results, as AHA/ACC probabilities tend to overestimate the probability of severe ischemia more than ESC probabilities.

**ELECTRONIC SUPPLEMENTARY MATERIAL**

**Part B**

**Analyses with the Old Version of Pre-Test Probability AHA/ACC**

**Based on Previous USA Guidelines (2012)**

**Reference and Abbreviation List** Page 24

**Figure SB1 Prevalence of Endpoints by Pre-Test USA (Old Version)** Page 25

**Figure SB2 Receiver Operating Characteristic Analysis** Page 26

**Figure SB3 Bayesian Combined Post-Test Probabilities** Page 27

**Figure SB4 Patient Flows for Abnormal Perfusion** Page 28

**Figure SB5 Calibration Plots** Page 29

**Figure SB6 Distribution of Probabilities** Page 30

**Figure SB7 Discrimination Capacity** Page 31

**Table SB1 Threshold Table for Pre-Test and Post-Test Probability USA (Old Version)** Page 34

**Table B2 Multivariable Analysis with Pre-Test Probability USA (Old Version)** Page 35

**Table Group B3 Reclassification Analyses** Page 36

**Reference for Previous USA Guidelines**

Fihn SD, Gardin JM, Abrams J, et al. 2012 ACCF/AHA/ACP/AATS/PCNA/SCAI/STS Guideline for the diagnosis and management of patients with stable ischemic heart disease: a report of the American College of Cardiology Foundation/American Heart Association Task Force on Practice Guidelines, and the American College of Physicians, American Association for Thoracic Surgery, Preventive Cardiovascular Nurses Association, Society for Cardiovascular Angiography and Interventions, and Society of Thoracic Surgeons. *J Am Coll Cardiol*. Dec 18 2012;60(24):e44-e164. doi:10.1016/j.jacc.2012.07.013

**Abbreviation List**

AHA/ACC American Heart Association/American College of Cardiology.

CAD Coronary artery disease.

ESC European Society of Cardiology.

IDI Integrated discrimination improvement

ILR Interval likelihood ratio

MFR Myocardial flow reserve

NRI Net reclassification improvement

PET Position emission tomography.

**Figure B1 Prevalence of Endpoints by Pre-Test-USA (Old Version)**


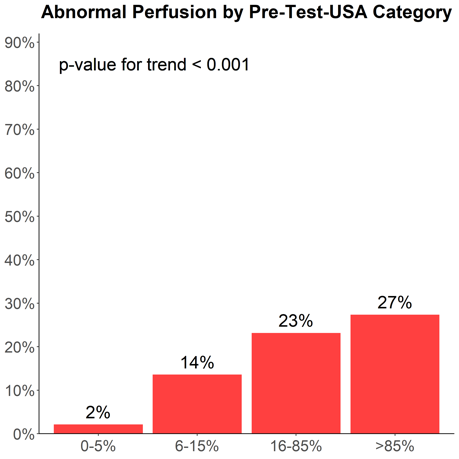

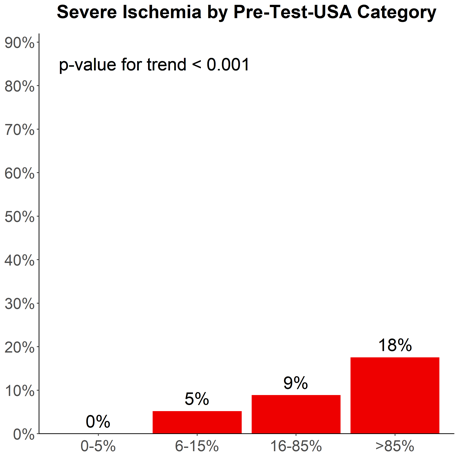

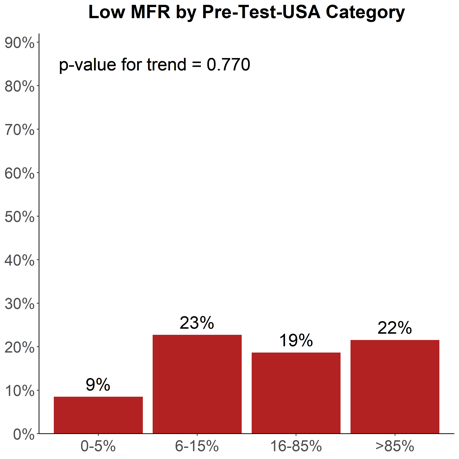


The prevalence of abnormal perfusion, severe ischemia and low MFR are presented by pre-test-USA categories. Increasing trends were found for each association and confirmed with the Cochran-Armitage test, except for low MFR. Abnormal perfusion and severe ischemia were rare in pre-test-USA 0–5%.

**Figure B2 Receiver Operating Characteristic Analysis**

**
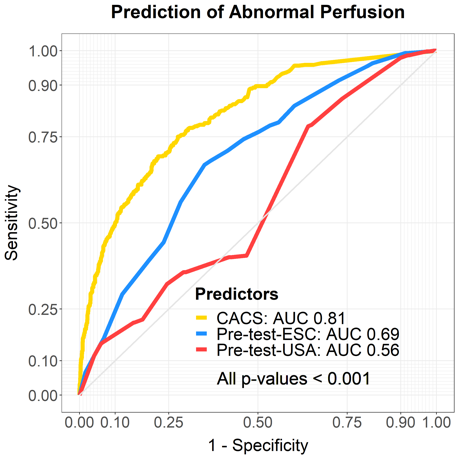

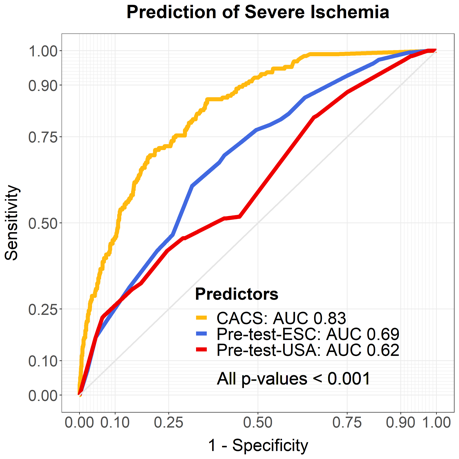

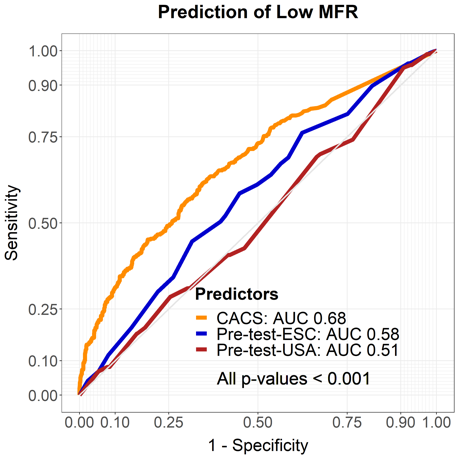
**

Receiver operating characteristic analysis is presented by endpoint and by predictor. CACS exhibited the highest AUC, followed by pre-test-ESC and pre-test-USA to predict abnormal perfusion, severe ischemia and low MFR (all p<0.001 for comparisons with Delong’s method).

**Figure B3 Bayesian Combined Post-Test Probabilities**


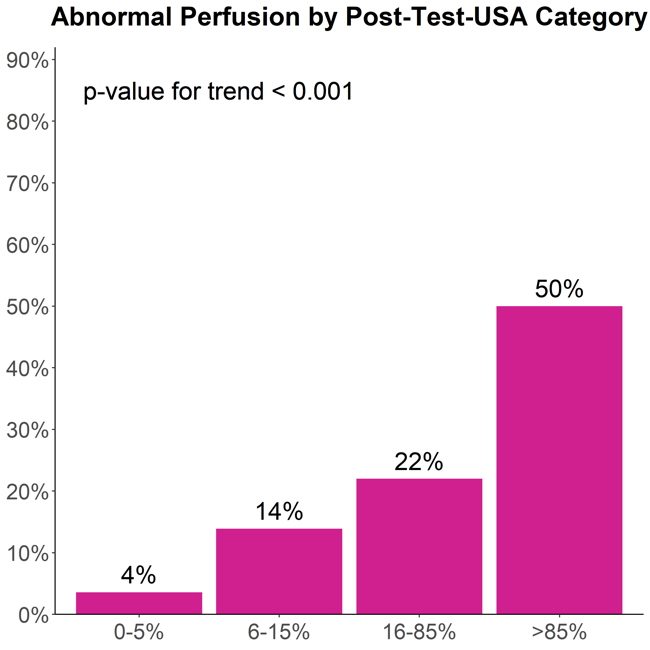

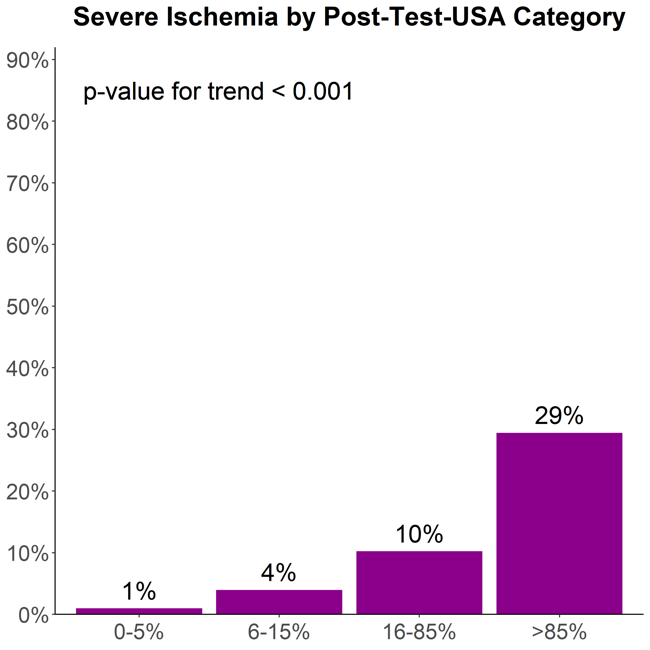

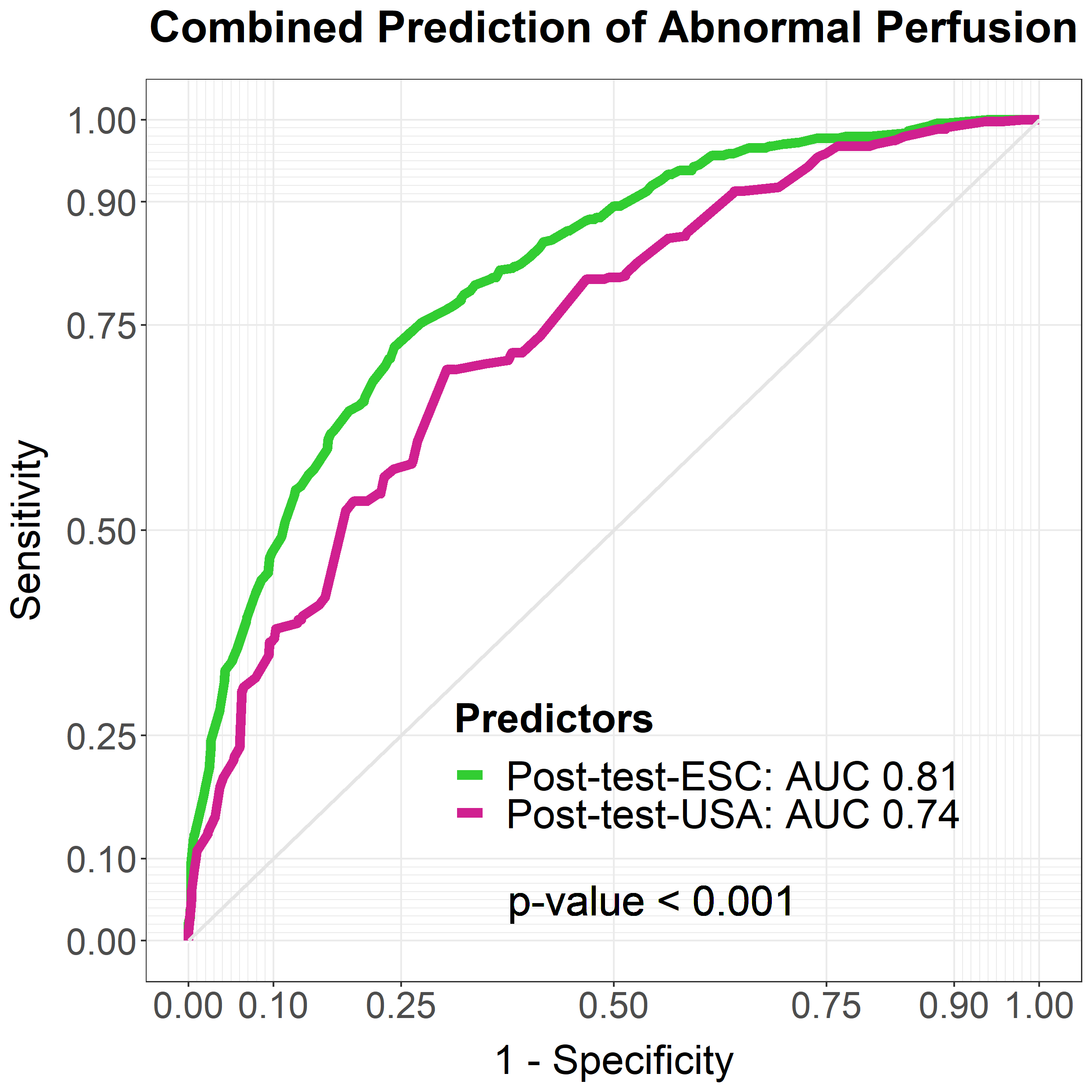

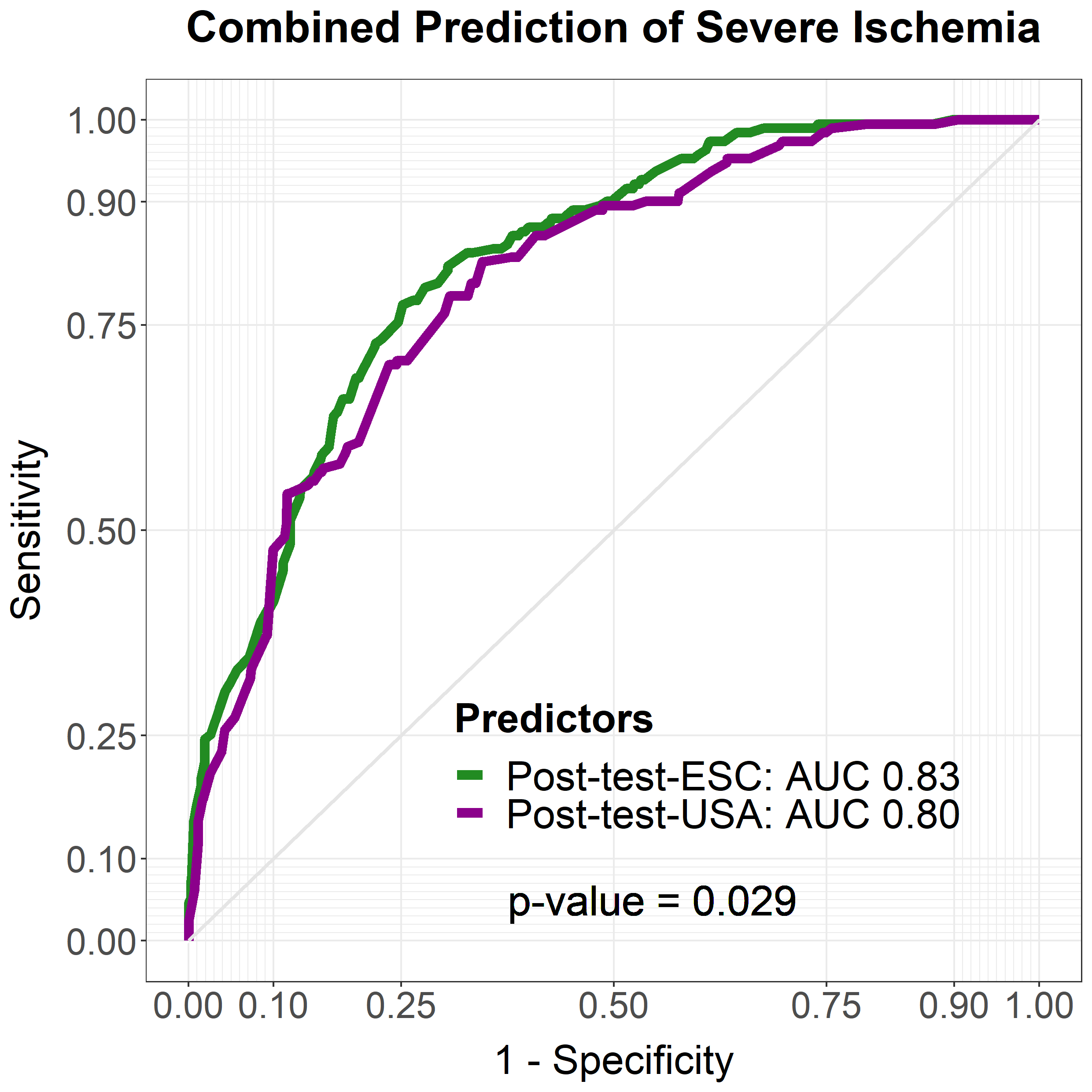


Using Bayes’ formula, pre-test probabilities from USA or ESC were combined with CACS into post-test probabilities (post-test-USA old version and post-test-ESC). Bar charts present the prevalence of abnormal perfusion and severe ischemia by post-test probability categories. Trends were assessed with the Cochran-Armitage test. Receiver operating characteristic curves demonstrated higher AUC with post-test-ESC versus post-test-USA for both endpoints (p<0.001 and p=0.029 for comparisons with DeLong’s method).

**Figure B4 Patient Flows for Abnormal Perfusion**

**
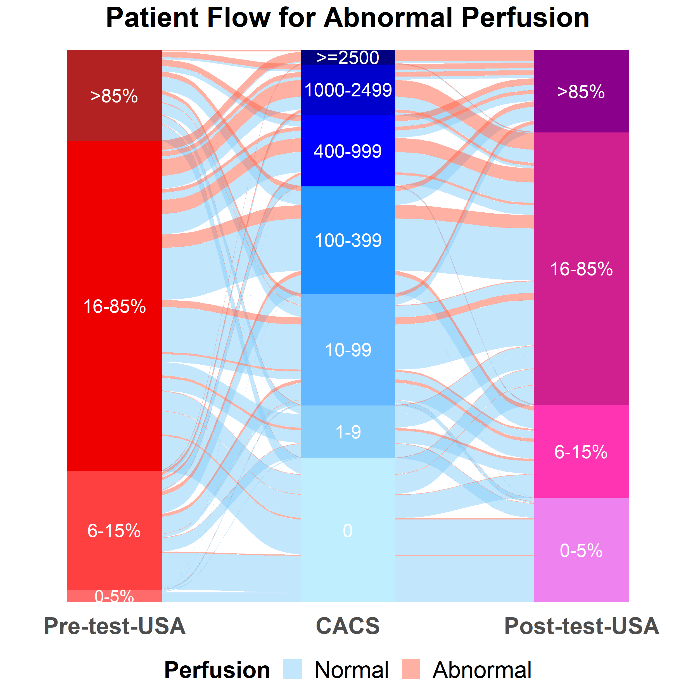

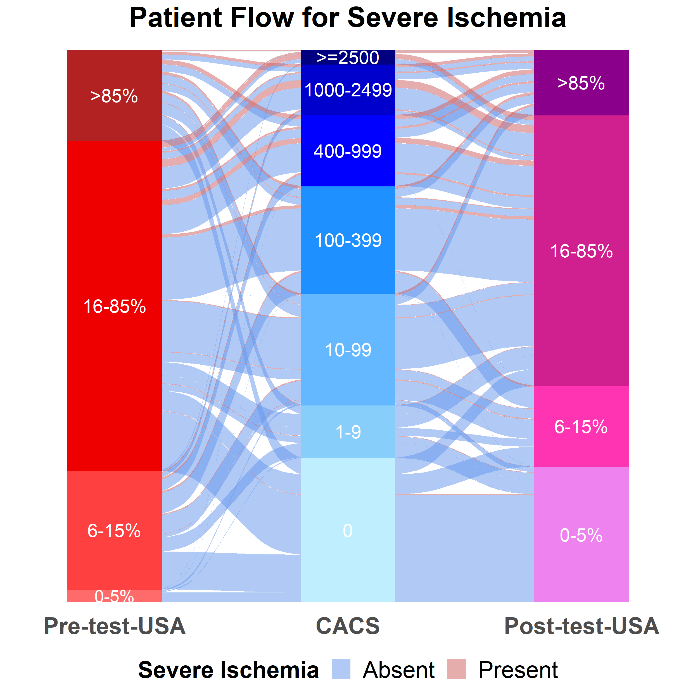
**

Patient flows through categories of pre-test probability, CACS, and post-test probability are shown by endpoint.

**Figure B5 Calibration Plots**

**
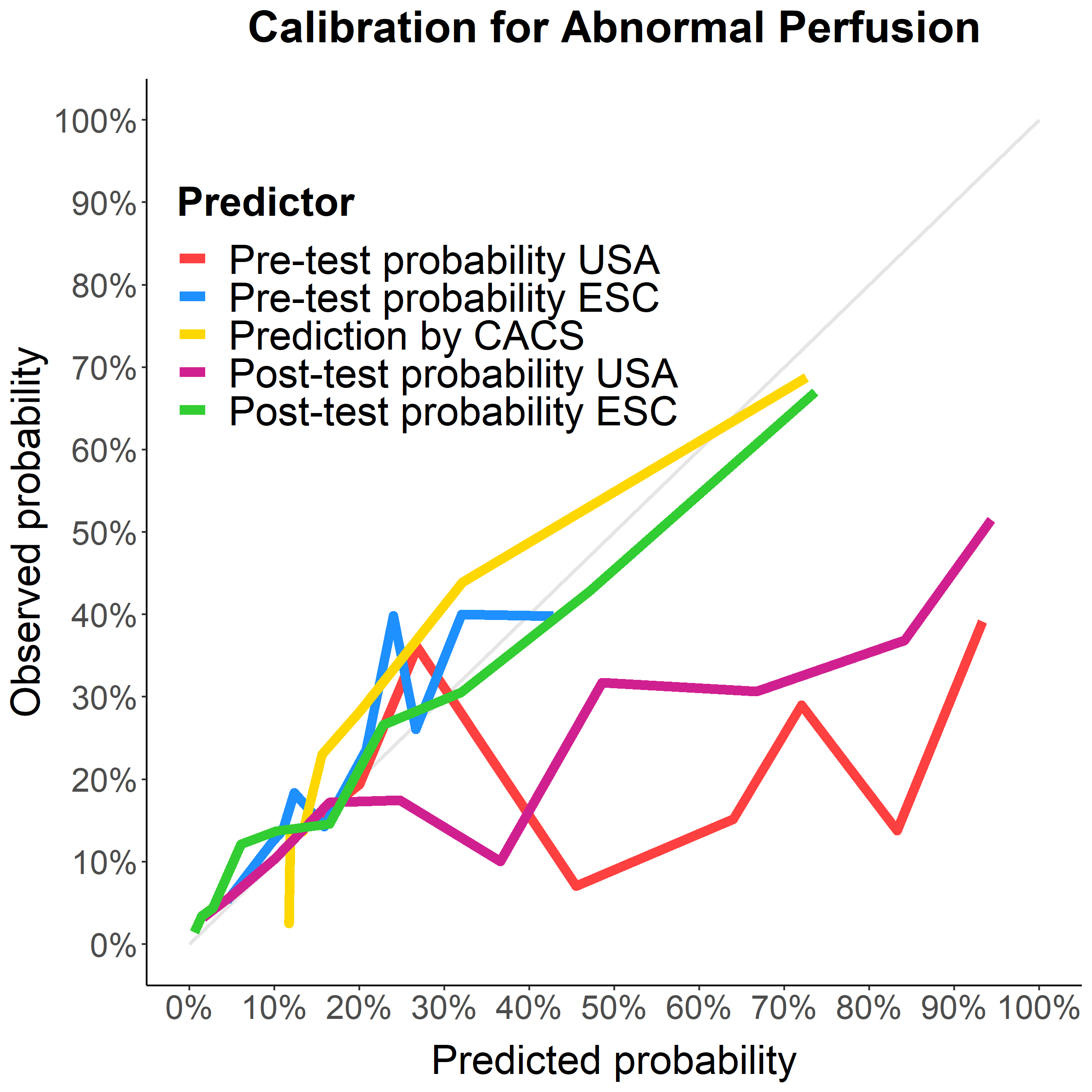

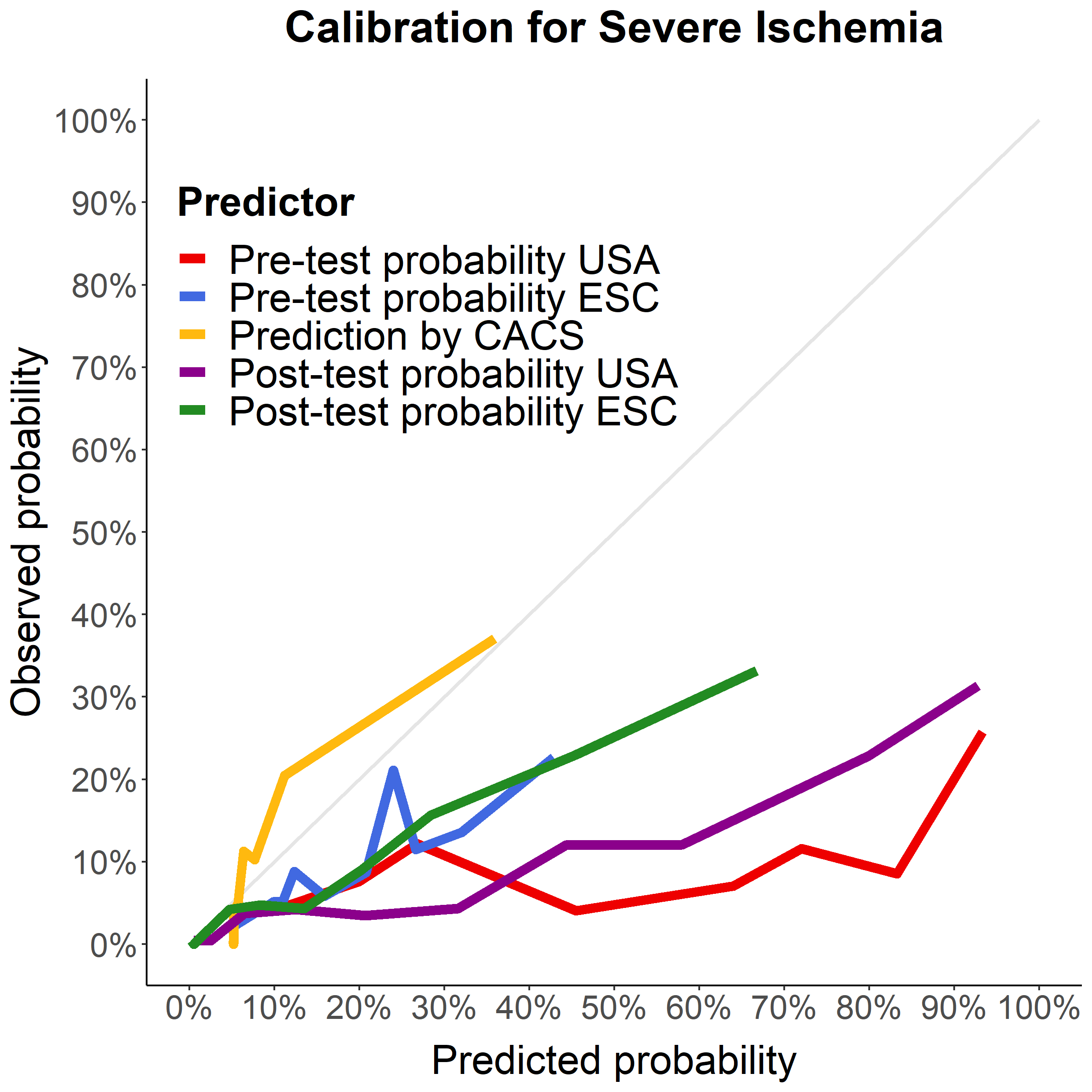
**

Calibration plots compare predicted versus observed probabilities of abnormal perfusion or severe ischemia, with the diagonal line as a reference for equal predicted and observed probabilities. USA probabilities (old version) overestimate abnormal perfusion and severe ischemia in medium-to-high probability categories, while ESC probabilities are better calibrated. Because severe ischemia is a subgroup of patients with abnormal perfusion, it was generally overestimated, more by USA than ESC probabilities. CACS needed transformation to probabilities using logistic regression.

AHA/ACC: American Heart Association/American College of Cardiology. ESC: European Society of Cardiology.

**Figure B6 Distribution of Probabilities**

**
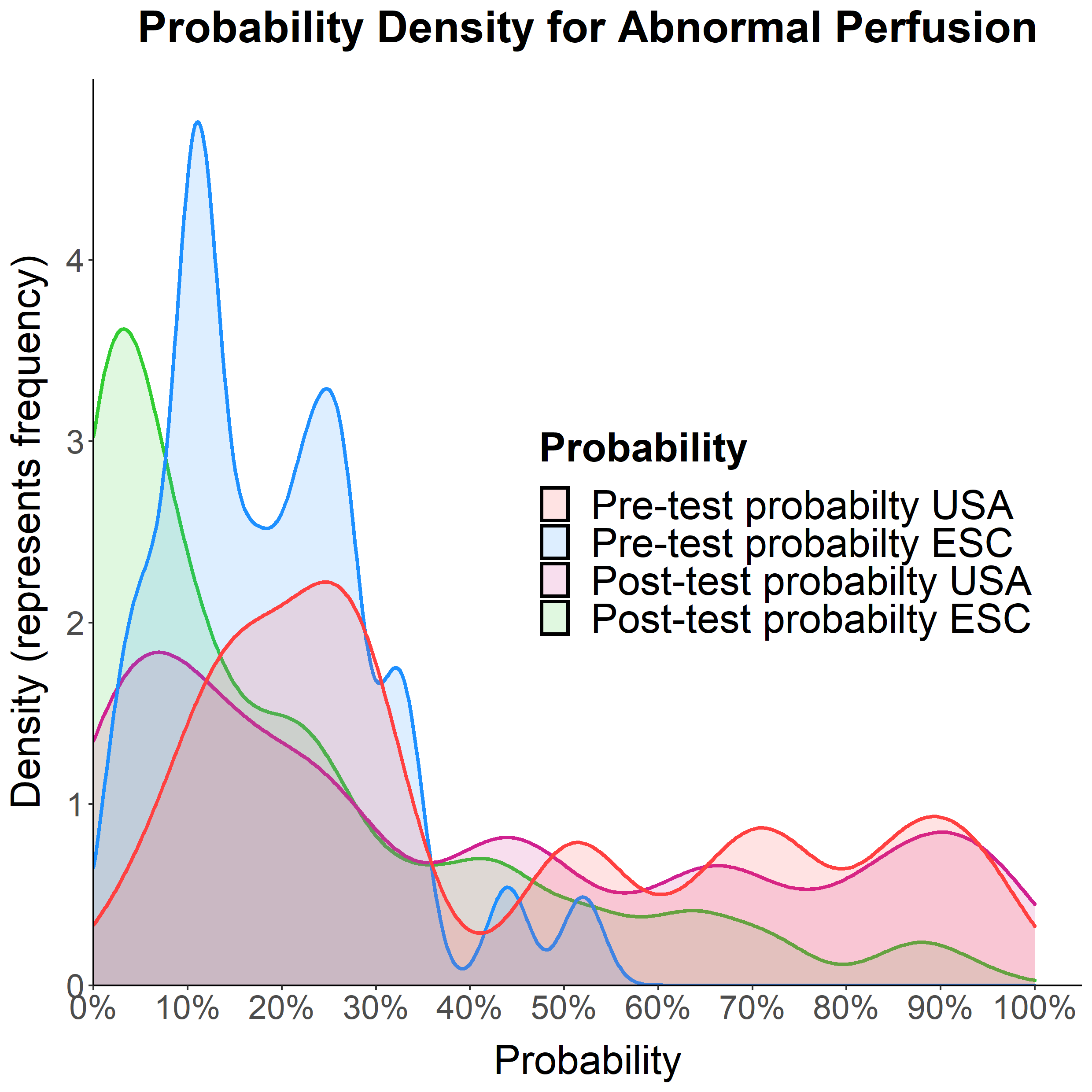

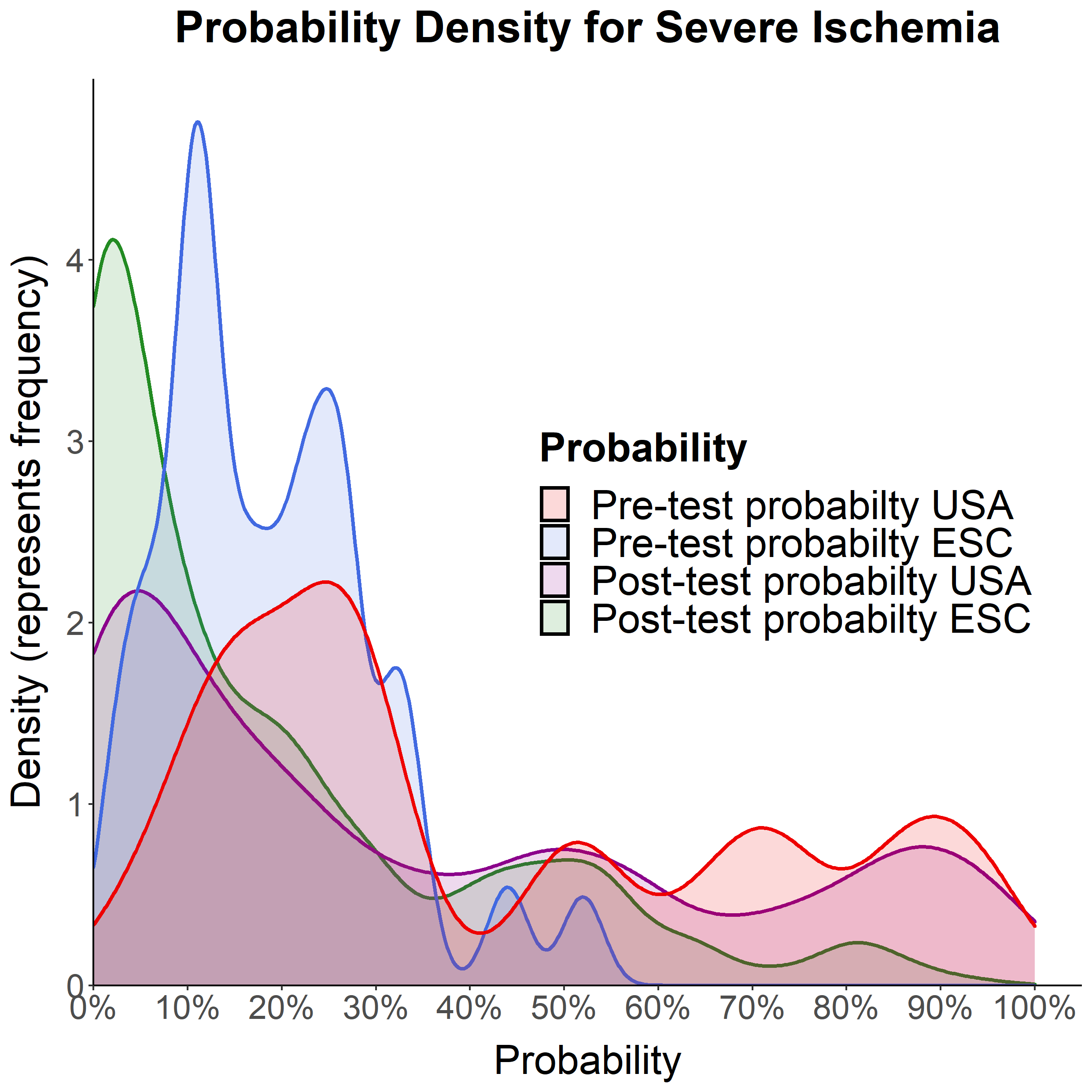
**

These graphs present the frequencies of each probability for abnormal perfusion and severe ischemia in density form (smooth equivalent to histogram). They show a clear shift towards lower predicted probabilities with post-test probabilities, more pronounced with ESC (high green spikes at low probabilities on each graph) than with USA (old version, purple spikes).

**Figure B7 Discrimination Capacity**


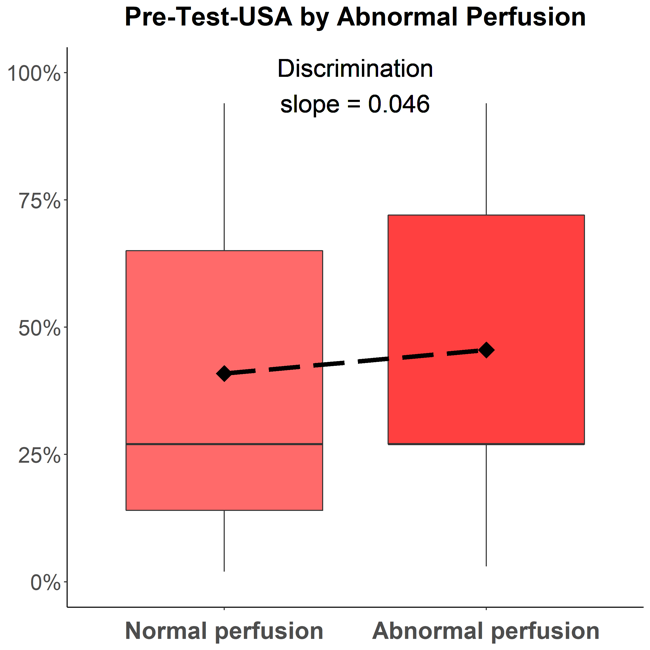

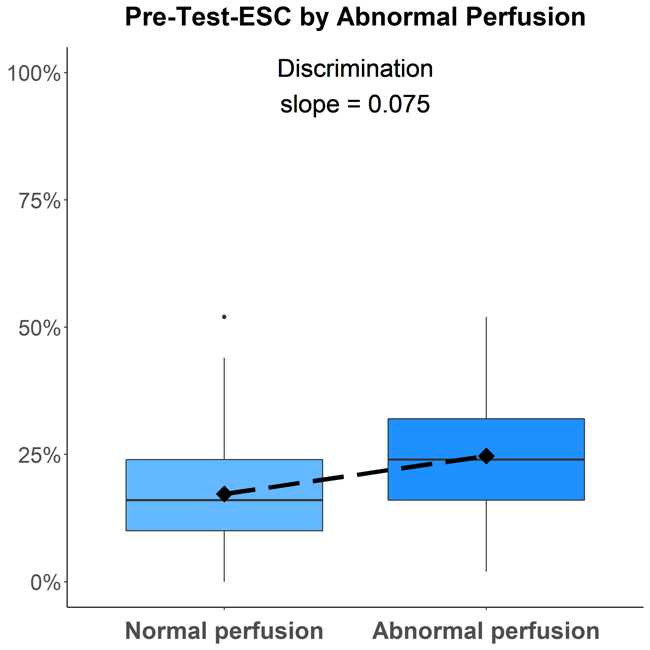


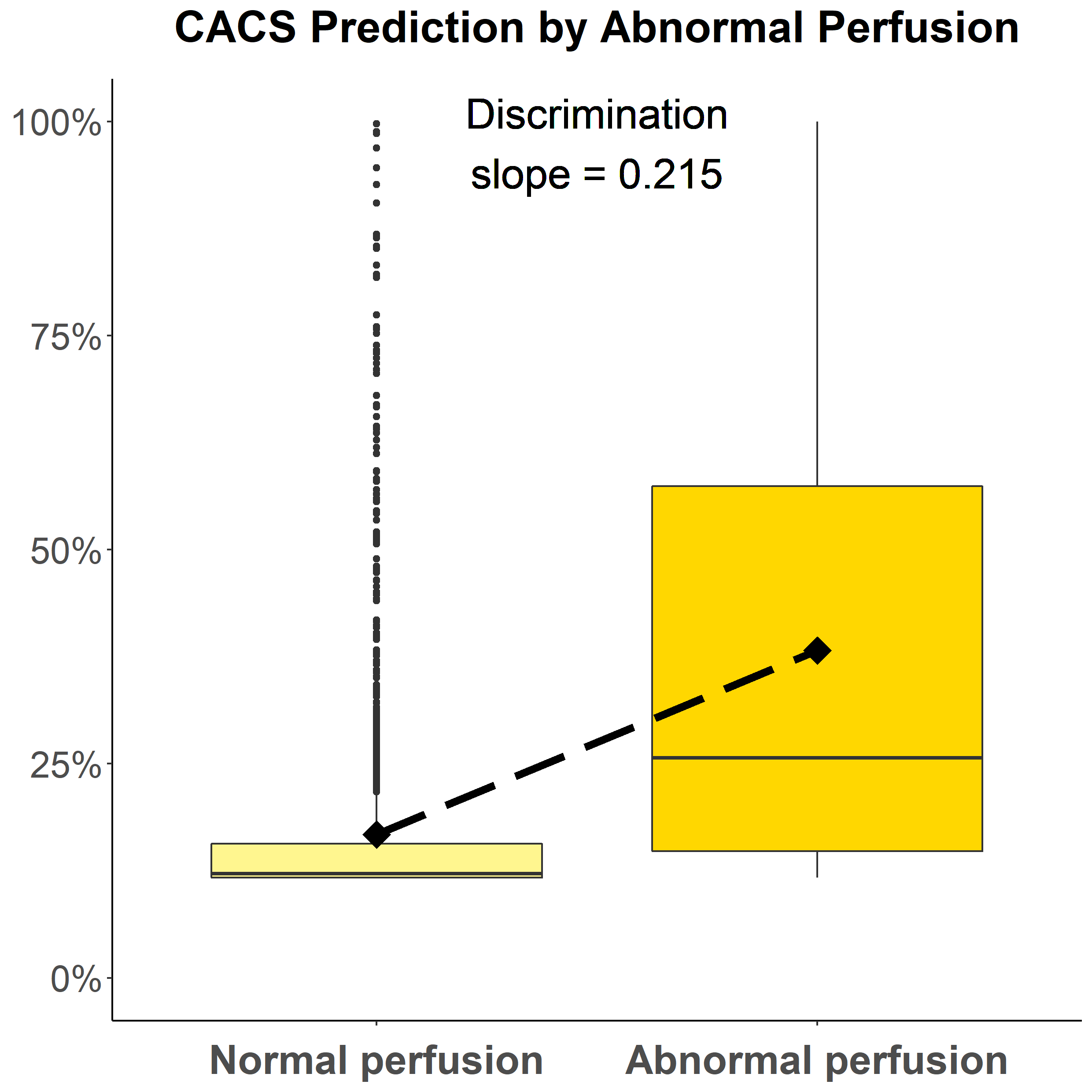


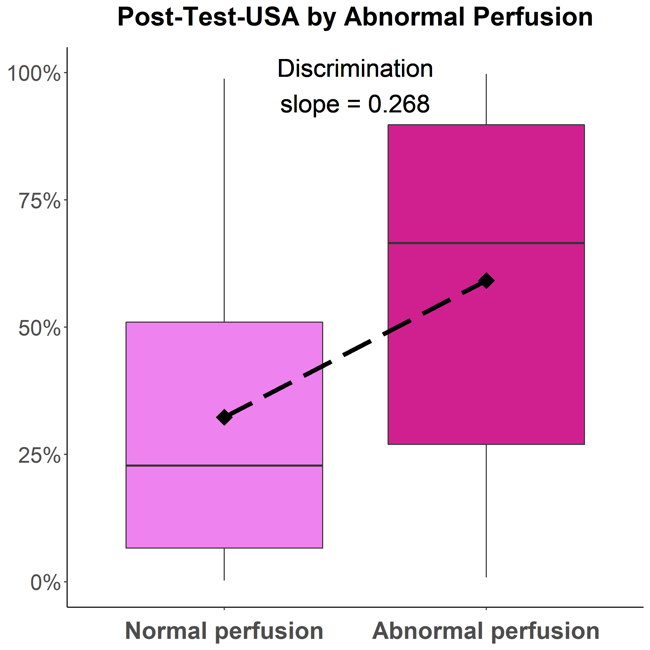

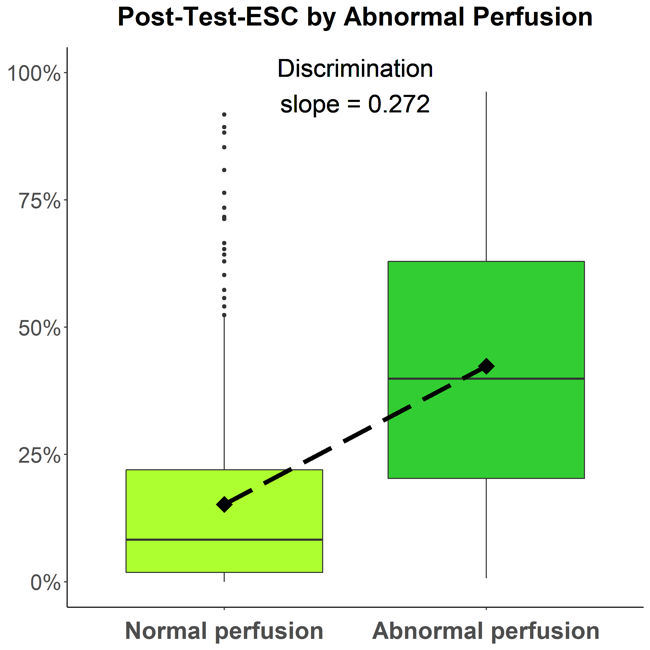


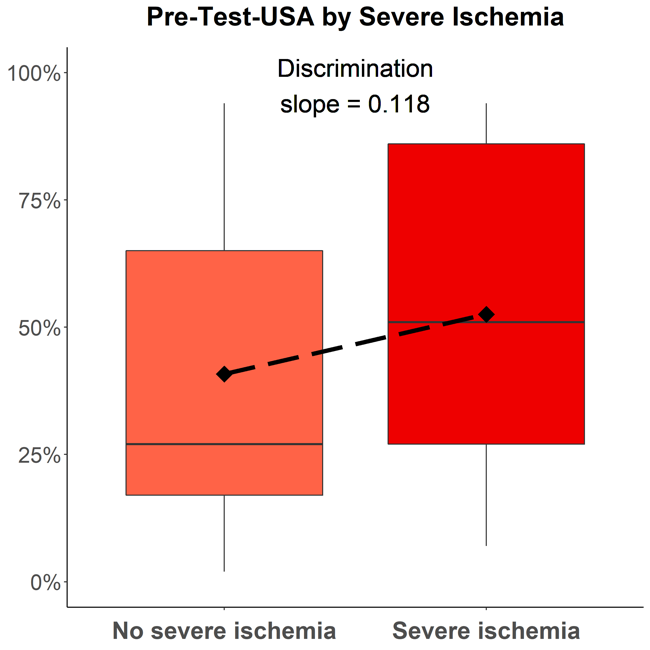

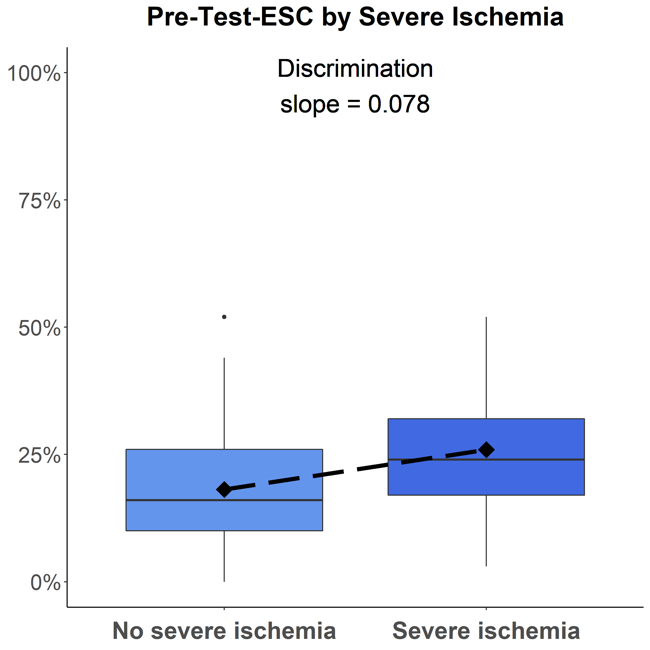

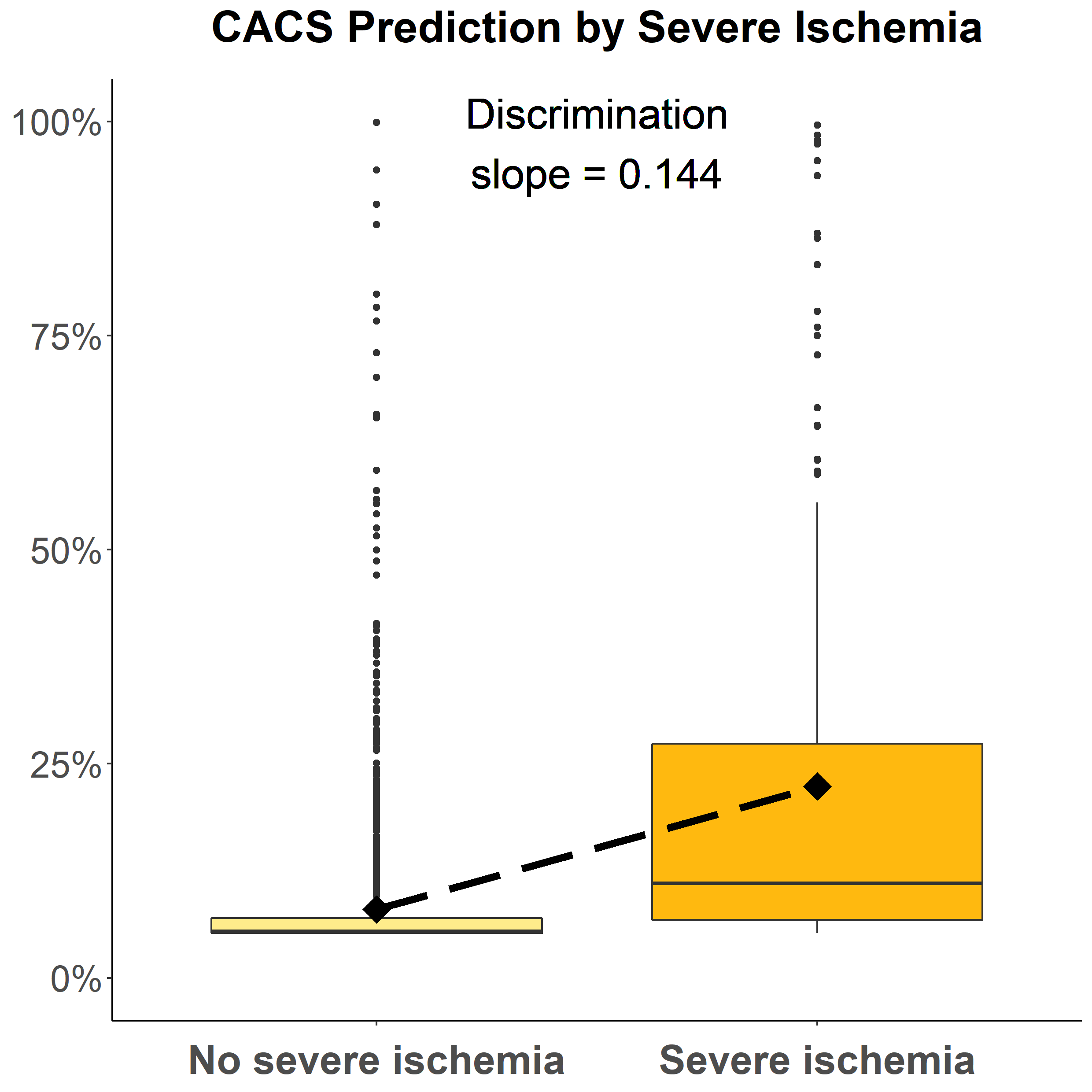


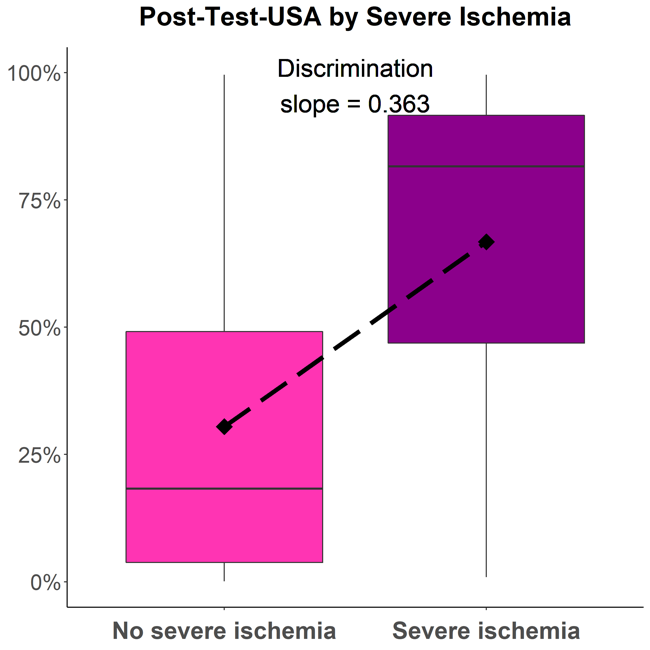

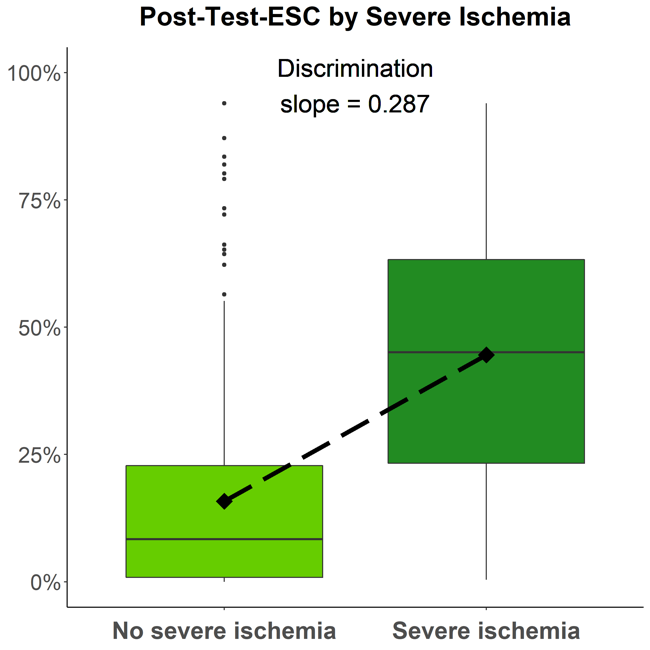


Each box plot presents the values of a predictor for patients without versus with the endpoint. CACS needed transformation to probabilities using logistic regression. Means are shown as black diamonds, and their differences are shown as dashed lines (discrimination slopes). Steeper slopes indicate better endpoint discrimination. The integrated discrimination improvement (IDI) is the difference between discrimination slopes. The pre-test-USA and post-test-USA presented here are based on the old version.

For abnormal perfusion, we found the following IDI: pre-test-USA versus pre-test-ESC 0.029 (0.004–0.053, p=0.024), pre-test-USA versus CACS 0.169 (0.127–0.210, p<0.001), pre-test-ESC versus CACS 0.140 (0.112–0.168, p<0.001), CACS versus post-test-USA 0.054 (0.024–0.083, p<0.001), CACS versus post-test-ESC 0.057 (0.041–0.073, p<0.001), pre-test-USA versus post-test-USA 0.222 (0.199–0.245, p<0.001), pre-test-ESC versus post-test-ESC 0.197 (0.175–0.219, p<0.001), and post-test-USA versus post-test-ESC 0.003 (-0.018–0.025, p=0.765).

For severe ischemia, we found the following IDI: pre-test-USA versus pre-test-ESC -0.039 (-0.075–-0.003, p=0.033), pre-test-USA versus CACS 0.026 (-0.035–0.087, p=0.398), pre-test-ESC versus CACS 0.065 (0.029–0.102, p<0.001), CACS versus post-test-USA 0.219 (0.174–0.265, p<0.001), CACS versus post-test-ESC 0.144 (0.118–0.170, p<0.001), pre-test-USA versus post-test-USA 0.245 (0.215–0.276, p<0.001), pre-test-ESC versus post-test-ESC 0.209 (0.178–0.240, p<0.001), and post-test-USA versus post-test-ESC -0.075 (-0.109–-0.042, p<0.001).

Overall, this analysis highlights the increased discrimination capacity from post-test probabilities. Comparison of USA (old version) versus ESC probabilities are limited by the different calibration. Indeed, USA probabilities overestimate endpoint prevalence at the medium-to-high probability categories, which increases USA discrimination slopes.

**Table B1 Threshold Table for Pre-Test and Post-Test Probability USA (Old Version)**

| **Threshold** | **Cumulative proportion** | **Sensitivity** | **Specificity** | **PPV** | **NPV** | **PLR** | **NLR** |
| --- | --- | --- | --- | --- | --- | --- | --- |
| **Abnormal perfusion** | | | | | | | |
| **Pre-test probability USA (Old Version)** | | | | | | | |
| **5%** | 2.3% | 99.8% | 2.9% | 21.8% | 97.9% | 1.03 | 0.08 |
| **15%** | 23.8% | 86.0% | 26.5% | 24.1% | 87.5% | 1.17 | 0.53 |
| **85%** | 83.6% | 21.1% | 84.9% | 27.4% | 79.9% | 1.39 | 0.93 |
| **Post-test probability USA: Combination of pre-test probability USA (Old) with CACS** | | | | | | | |
| **5%** | 18.9% | 96.8% | 23.2% | 25.5% | 96.4% | 1.26 | 0.14 |
| **15%** | 35.8% | 85.8% | 41.6% | 28.5% | 91.5% | 1.47 | 0.34 |
| **85%** | 85.2% | 34.8% | 90.6% | 50.0% | 83.7% | 3.69 | 0.72 |
| **Severe ischemia** | | | | | | | |
| **Pre-test probability USA (Old Version)** | | | | | | | |
| **5%** | 2.3% | 100% | 2.5% | 9.5% | 100% | 1.03 | 0.00 |
| **15%** | 23.8% | 88.0% | 25.0% | 10.8% | 95.3% | 1.17 | 0.48 |
| **85%** | 83.6% | 30.9% | 85.1% | 17.6% | 92.3% | 2.07 | 0.81 |
| **Post-test probability USA: Combination of pre-test probability USA (Old) with CACS** | | | | | | | |
| **5%** | 24.5% | 97.4% | 26.8% | 12.0% | 99.0% | 1.33 | 0.10 |
| **15%** | 39.2% | 91.1% | 42.3% | 14.0% | 97.9% | 1.58 | 0.21 |
| **85%** | 88.2% | 37.2% | 90.9% | 29.5% | 93.4% | 4.06 | 0.69 |

Each row presents the predictive performance of the given threshold.

NLR: negative likelihood ratio. NPV: negative predictive value. PLR: positive likelihood ratio. PPV: positive predictive value.

**Table B2 Multivariable Analysis with Pre-Test Probability USA (Old Version)**

| Predictors | Abnormal perfusion | Severe ischemia | Low MFR |
| --- | --- | --- | --- |
| Pre-test-USA (%) | 1.01 (1.00–1.01) p=0.007 | 1.02 (1.01–1.02) p<0.001 | 1.00 (1.00–1.00) p=0.889 |
| Body mass index | 0.98 (0.96–1.00) p=0.249 | 0.97 (0.94–1.00) p=0.073 | 0.97 (0.95–0.99) p=0.004 |
| Insulin-dependent diabetes mellitus | 1.08 (0.67–1.70) p=0.815 | 1.64 (0.91–2.83) p=0.117 | 2.29 (1.52–3.43) p<0.001 |
| Non-insulin-dependent diabetes mellitus | 1.18 (0.85–1.62) p=0.415 | 1.50 (0.98–2.27) p=0.094 | 1.39 (1.02–1.89) p=0.051 |
| Hypercholesterolemia | 1.13 (0.88–1.45) p=0.415 | 1.88 (1.33–2.68) p=0.001 | 0.99 (0.78–1.26) p=0.954 |
| Arterial hypertension | 1.24 (0.94–1.65) p=0.249 | 1.17 (0.78–1.76) p=0.556 | 1.55 (1.18–2.06) p=0.004 |
| Active smoking | 1.16 (0.85–1.57) p=0.415 | 1.82 (1.22–2.71) p=0.007 | 0.75 (0.55–1.01) p=0.073 |
| Previous smoking | 1.24 (0.93–1.64) p=0.249 | 1.07 (0.71–1.60) p=0.815 | 0.68 (0.51–0.90) p=0.012 |
| Family history of CAD | 1.01 (0.76–1.33) p=0.949 | 1.03 (0.69–1.51) p=0.896 | 0.53 (0.38–0.71) p<0.001 |
| CACS (by 100 units) | 1.17 (1.14–1.19) p<0.001 | 1.11 (1.09–1.13) p<0.001 | 1.08 (1.06–1.09) p<0.001 |

Results are presented as odds ratios with 95% confidence intervals from multivariable logistic regressions. P-values are adjusted for multiple comparisons using the Benjamini-Hochberg sequential procedure, which shows the false discovery rate.

**Table Group B3 Reclassification Analyses**

Each table presents the classification of patients according to a predictor in rows and to another predictor in columns. The upper part shows patients with the endpoint and the lower part patients without the endpoint. Green cells indicate “correct” reclassifications by the second predictor in columns (patients with endpoint to higher probability to the right, patients without endpoint to lower probability to the left). Red cells indicate “incorrect” reclassifications by the second predictor in columns (patients with endpoint to lower probability to the left, patients without endpoint to higher probability to the right). Yellow cells indicate patients with the same classification by both predictors. The net reclassification improvement (NRI) sums up the proportions of “correct” minus “incorrect” reclassifications.

**Table B3a Pre-Test Probability USA (Old Version) versus Pre-Test Probability ESC for Abnormal Perfusion**

| **Abnormal perfusion:** NRI 0.127 (0.077–0.177), p<0.001 | | | | | | |
| --- | --- | --- | --- | --- | --- | --- |
| **Abnormal**  **perfusion** | | **Pre-test-ESC** | | | | |
|  |  | **0–5%** | **6–15%** | **16–85%** | **>85%** | **Total** |
| **Pre-test-USA (old)** | **0–5%** | 1 | 0 | 0 | 0 | 1 |
|  | **6–15%** | 2 | 58 | 0 | 0 | 60 |
|  | **16–85%** | 0 | 42 | 242 | 0 | 284 |
|  | **>85%** | 0 | 0 | 92 | 0 | 92 |
|  | **Total** | 3 | 100 | 334 | 0 | 437 |
| **Normal**  **perfusion** | | **Pre-test-ESC** | | | | |
|  |  | **0–5%** | **6–15%** | **16–85%** | **>85%** | **Total** |
| **Pre-test-USA (old)** | **0–5%** | 46 | 0 | 0 | 0 | 46 |
|  | **6–15%** | 87 | 294 | 0 | 0 | 381 |
|  | **16–85%** | 10 | 366 | 566 | 0 | 942 |
|  | **>85%** | 0 | 0 | 244 | 0 | 244 |
|  | **Total** | 143 | 660 | 810 | 0 | 1613 |

With pre-test USA (old version) as a reference, using pre-test ESC in patients with abnormal perfusion (upper table) moved 0 patient correctly to the right to higher probabilities and 136 (31.1%) incorrectly to the left to lower probabilities. In patients with normal perfusion (lower table), it moved 707 patients (43.8%) correctly to the left to lower probabilities and 0 incorrectly to the right to higher probabilities. The NRI was 0.127 (0.077–0.177, p<0.001) in favor of pre-test-ESC, due to the numerous correct reclassifications to lower probability categories.

The different calibration of USA (old version) and ESC probabilities affects these results, as USA probabilities overestimate the probability of abnormal perfusion, while ESC probabilities are more accurate.

**Table B3b Pre-Test Probability USA (Old Version) versus Post-Test Probability USA for Abnormal Perfusion**

| **Abnormal perfusion:** NRI 0.450 (0.393–0.507), p<0.001 | | | | | | |
| --- | --- | --- | --- | --- | --- | --- |
| **Abnormal**  **perfusion** | | **Post-test-USA (pre-test-USA old version with CACS)** | | | | |
|  |  | **0–5%** | **6–15%** | **16–85%** | **>85%** | **Total** |
| **Pre-test-USA (old)** | **0–5%** | 1 | 0 | 0 | 0 | 1 |
|  | **6–15%** | 7 | 30 | 23 | 0 | 60 |
|  | **16–85%** | 6 | 18 | 192 | 68 | 284 |
|  | **>85%** | 0 | 0 | 8 | 84 | 92 |
|  | **Total** | 14 | 48 | 223 | 152 | 437 |
| **Normal**  **perfusion** | | **Post-test-USA (pre-test-USA old version with CACS)** | | | | |
|  |  | **0–5%** | **6–15%** | **16–85%** | **>85%** | **Total** |
| **Pre-test-USA (old)** | **0–5%** | 45 | 1 | 0 | 0 | 46 |
|  | **6–15%** | 199 | 134 | 48 | 0 | 381 |
|  | **16–85%** | 130 | 162 | 607 | 43 | 942 |
|  | **>85%** | 0 | 0 | 135 | 109 | 244 |
|  | **Total** | 374 | 297 | 790 | 152 | 1613 |

With pre-test-USA (old version) as a reference, using post-test-USA in patients with abnormal perfusion (upper table) moved 91 patients (20.8%) correctly to the right to higher probabilities and 39 (8.9%) incorrectly to the left to lower probabilities. In patients with normal perfusion (lower table), it moved 628 patients (38.8%) correctly to the left to lower probabilities and 92 (5.7%) incorrectly to the right to higher probabilities. The NRI was 0.450 (0.393–0.507, p<0.001) in favor of post-test-USA, due to the numerous correct reclassifications to lower probability categories.

**Table B3c Post-Test Probability USA (Old Version) versus Post-Test Probability ESC for Abnormal Perfusion**

| **Abnormal perfusion:** NRI 0.105 (0.055–0.155), p<0.001 | | | | | | |
| --- | --- | --- | --- | --- | --- | --- |
| **Abnormal**  **perfusion** | | **Post-test-ESC** | | | | |
|  |  | **0–5%** | **6–15%** | **16–85%** | **>85%** | **Total** |
| **Post-test -USA (Old)** | **0–5%** | 13 | 1 | 0 | 0 | 14 |
|  | **6–15%** | 9 | 38 | 1 | 0 | 48 |
|  | **16–85%** | 5 | 18 | 200 | 0 | 223 |
|  | **>85%** | 0 | 0 | 98 | 54 | 152 |
|  | **Total** | 27 | 57 | 299 | 54 | 437 |
| **Normal**  **perfusion** | | **Post-test-ESC** | | | | |
|  |  | **0–5%** | **6–15%** | **16–85%** | **>85%** | **Total** |
| **Post-test-USA (Old)** | **0–5%** | 371 | 3 | 0 | 0 | 374 |
|  | **6–15%** | 137 | 160 | 0 | 0 | 297 |
|  | **16–85%** | 152 | 213 | 425 | 0 | 790 |
|  | **>85%** | 0 | 0 | 143 | 9 | 152 |
|  | **Total** | 660 | 376 | 568 | 9 | 1613 |

With post-test-USA (old version) as a reference, using post-test-ESC in patients with abnormal perfusion (upper table) moved 2 patients (0.5%) correctly to the right to higher probabilities and 130 (29.7%) incorrectly to the left to lower probabilities. In patients with normal perfusion (lower table), it moved 645 patients (40.0%) correctly to the left to lower probabilities and 3 (0.2%) incorrectly to the right to higher probabilities. The NRI was 0.105 (0.055–0.155, p<0.001) in favor of post-test-ESC, mostly due to the numerous correct reclassifications to lower probability categories.

The different calibration of USA (old version) and ESC probabilities affects these results, as USA probabilities overestimate the probability of abnormal perfusion, while ESC probabilities are more accurate.

**Table B3d Pre-Test Probability USA (Old Version) versus Pre-Test Probability ESC for Severe Ischemia**

| **Severe ischemia:** NRI 0.003 (-0.070–0.076), p=0.933 | | | | | | |
| --- | --- | --- | --- | --- | --- | --- |
| **Severe**  **ischemia** | | **Pre-test-ESC** | | | | |
|  |  | **0–5%** | **6–15%** | **16–85%** | **>85%** | **Total** |
| **Pre-test-USA (old)** | **0–5%** | 0 | 0 | 0 | 0 | 0 |
|  | **6–15%** | 1 | 22 | 0 | 0 | 23 |
|  | **16–85%** | 0 | 18 | 91 | 0 | 109 |
|  | **>85%** | 0 | 0 | 59 | 0 | 59 |
|  | **Total** | 1 | 40 | 150 | 0 | 191 |
| **No severe**  **ischemia** | | **Pre-test-ESC** | | | | |
|  |  | **0–5%** | **6–15%** | **16–85%** | **>85%** | **Total** |
| **Pre-test-USA (old)** | **0–5%** | 47 | 0 | 0 | 0 | 47 |
|  | **6–15%** | 88 | 330 | 0 | 0 | 418 |
|  | **16–85%** | 10 | 390 | 717 | 0 | 1117 |
|  | **>85%** | 0 | 0 | 277 | 0 | 277 |
|  | **Total** | 145 | 720 | 994 | 0 | 1859 |

With pre-test-USA (old version) as a reference, using pre-test-ESC in patients with severe ischemia (upper table) moved 0 patient correctly to the right to higher probabilities and 78 (40.8%) incorrectly to the left to lower probabilities. In patients without severe ischemia (lower table), it moved 765 patients (41.2%) correctly to the left to lower probabilities and 0 incorrectly to the right to higher probabilities. The NRI was 0.003 (-0.070–0.076, p=0.933), due to the balance between percentages of correct and incorrect reclassifications.

The different calibration of USA (old version) and ESC probabilities affects these results, as USA probabilities overestimate the probability of severe ischemia more than ESC probabilities.

**Table B3e Pre-Test Probability USA (Old Version) versus Post-Test Probability USA for Severe Ischemia**

| **Severe Ischemia:** NRI 0.430 (0.357–0.502), p<0.001 | | | | | | |
| --- | --- | --- | --- | --- | --- | --- |
| **Severe**  **ischemia** | | **Post-test-USA** (pre-test-USA old version with CACS) | | | | |
|  |  | **0–5%** | **6–15%** | **16–85%** | **>85%** | **Total** |
| **Pre-test-USA (old)** | **0–5%** | 0 | 0 | 0 | 0 | 0 |
|  | **6–15%** | 1 | 10 | 12 | 0 | 23 |
|  | **16–85%** | 4 | 2 | 84 | 19 | 109 |
|  | **>85%** | 0 | 0 | 7 | 52 | 59 |
|  | **Total** | 5 | 12 | 103 | 71 | 191 |
| **No severe**  **Ischemia** | | **Post-test-USA** (pre-test-USA old version with CACS) | | | | |
|  |  | **0–5%** | **6–15%** | **16–85%** | **>85%** | **Total** |
| **Pre-test-USA (old)** | **0–5%** | 46 | 1 | 0 | 0 | 47 |
|  | **6–15%** | 205 | 149 | 64 | 0 | 418 |
|  | **16–85%** | 247 | 139 | 673 | 58 | 1117 |
|  | **>85%** | 0 | 0 | 165 | 112 | 277 |
|  | **Total** | 498 | 289 | 902 | 170 | 1859 |

With pre-test-USA (old version) as a reference, using post-test-USA in patients with severe ischemia (upper table) moved 31 patients (16.2%) correctly to the right to higher probabilities and 14 (7.3%) incorrectly to the left to lower probabilities. In patients without severe ischemia (lower table), it moved 756 patients (40.7%) correctly to the left to lower probabilities and 123 (6.6%) incorrectly to the right to higher probabilities. The NRI was 0.430 (0.357–0.502, p<0.001) in favor of post-test-USA, due to the numerous correct reclassifications to lower probability categories.

**Table B3f Post-Test Probability USA (Old Version) versus Post-Test Probability ESC for Severe Ischemia**

| **Severe ischemia:** NRI -0.038 (-0.112–0.040), p=0.313 | | | | | | |
| --- | --- | --- | --- | --- | --- | --- |
| **Severe ischemia** | | **Post-test-ESC** | | | | |
|  |  | **0–5%** | **6–15%** | **16–85%** | **>85%** | **Total** |
| **Post-test-USA (Old)** | **0–5%** | 5 | 0 | 0 | 0 | 5 |
|  | **6–15%** | 2 | 10 | 0 | 0 | 12 |
|  | **16–85%** | 1 | 11 | 90 | 1 | 103 |
|  | **>85%** | 0 | 0 | 62 | 9 | 71 |
|  | **Total** | 8 | 21 | 152 | 10 | 191 |
| **No severe ischemia** | | **Post-test-ESC** | | | | |
|  |  | **0–5%** | **6–15%** | **16–85%** | **>85%** | **Total** |
| **Post-test-USA (Old)** | **0–5%** | 498 | 0 | 0 | 0 | 498 |
|  | **6–15%** | 120 | 169 | 0 | 0 | 289 |
|  | **16–85%** | 126 | 249 | 522 | 5 | 902 |
|  | **>85%** | 0 | 0 | 169 | 1 | 170 |
|  | **Total** | 744 | 418 | 691 | 6 | 1859 |

With post-test-USA (old version) as a reference, using post-test-ESC in patients with severe ischemia (upper table) moved 1 patient (0.5%) correctly to the right to higher probabilities and 76 (39.8%) incorrectly to the left to lower probabilities. In patients without severe ischemia (lower table), it moved 664 patients (35.7%) correctly to the left to lower probabilities and 5 (0.3%) incorrectly to the right to higher probabilities. The NRI was -0.038 (-0.112–0.040, p=0.313), due to the balance between percentages of correct and incorrect reclassifications.

The different calibration of USA (old version) and ESC probabilities affects these results, as USA probabilities overestimate the probability of severe ischemia more than ESC probabilities.
