# Supplementary material for: Coronary artery calcium score and pre-test probabilities as gatekeepers to predict and rule out perfusion defects in positron emission tomography
Source: J Nucl Cardiol. 2023 Jul 6;30(6):2559–73. doi: 10.1007/s12350-023-03322-3 (PMC10682222; doi:10.1007/s12350-023-03322-3)
Supplement: Supplementary file 2 — Supplementary file2 (PPTX 581 KB) [file 12350_2023_3322_MOESM2_ESM.pptx]

## Slide 1
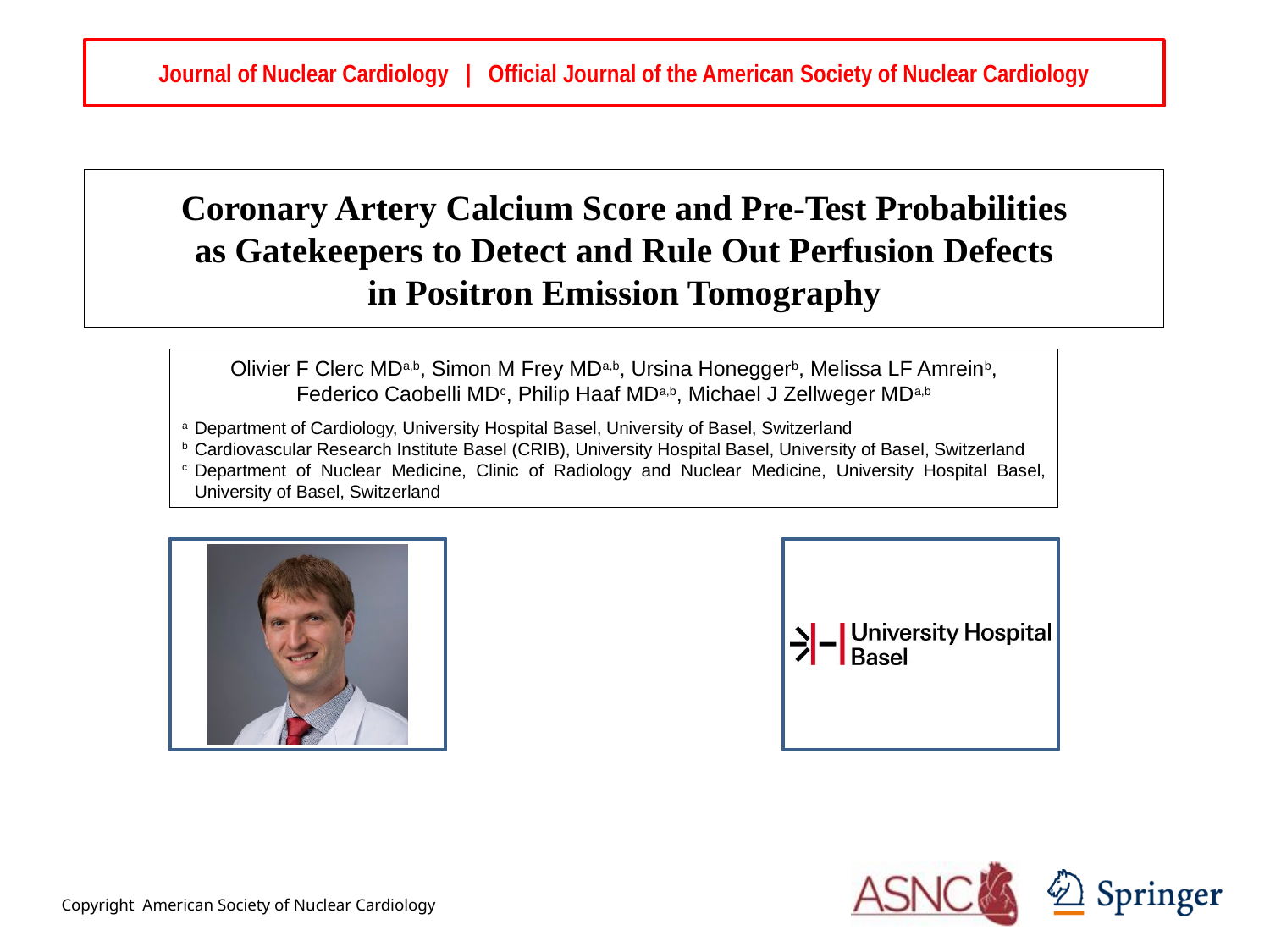

Journal of Nuclear Cardiology | Official Journal of the American Society of Nuclear Cardiology
# Coronary Artery Calcium Score and Pre-Test Probabilities as Gatekeepers to Detect and Rule Out Perfusion Defects in Positron Emission Tomography
Olivier F Clerc MDa,b, Simon M Frey MDa,b, Ursina Honeggerb, Melissa LF Amreinb,
Federico Caobelli MDc, Philip Haaf MDa,b, Michael J Zellweger MDa,b
a	Department of Cardiology, University Hospital Basel, University of Basel, Switzerland
b	Cardiovascular Research Institute Basel (CRIB), University Hospital Basel, University of Basel, Switzerland
c	Department of Nuclear Medicine, Clinic of Radiology and Nuclear Medicine, University Hospital Basel, University of Basel, Switzerland
Copyright American Society of Nuclear Cardiology

## Slide 2
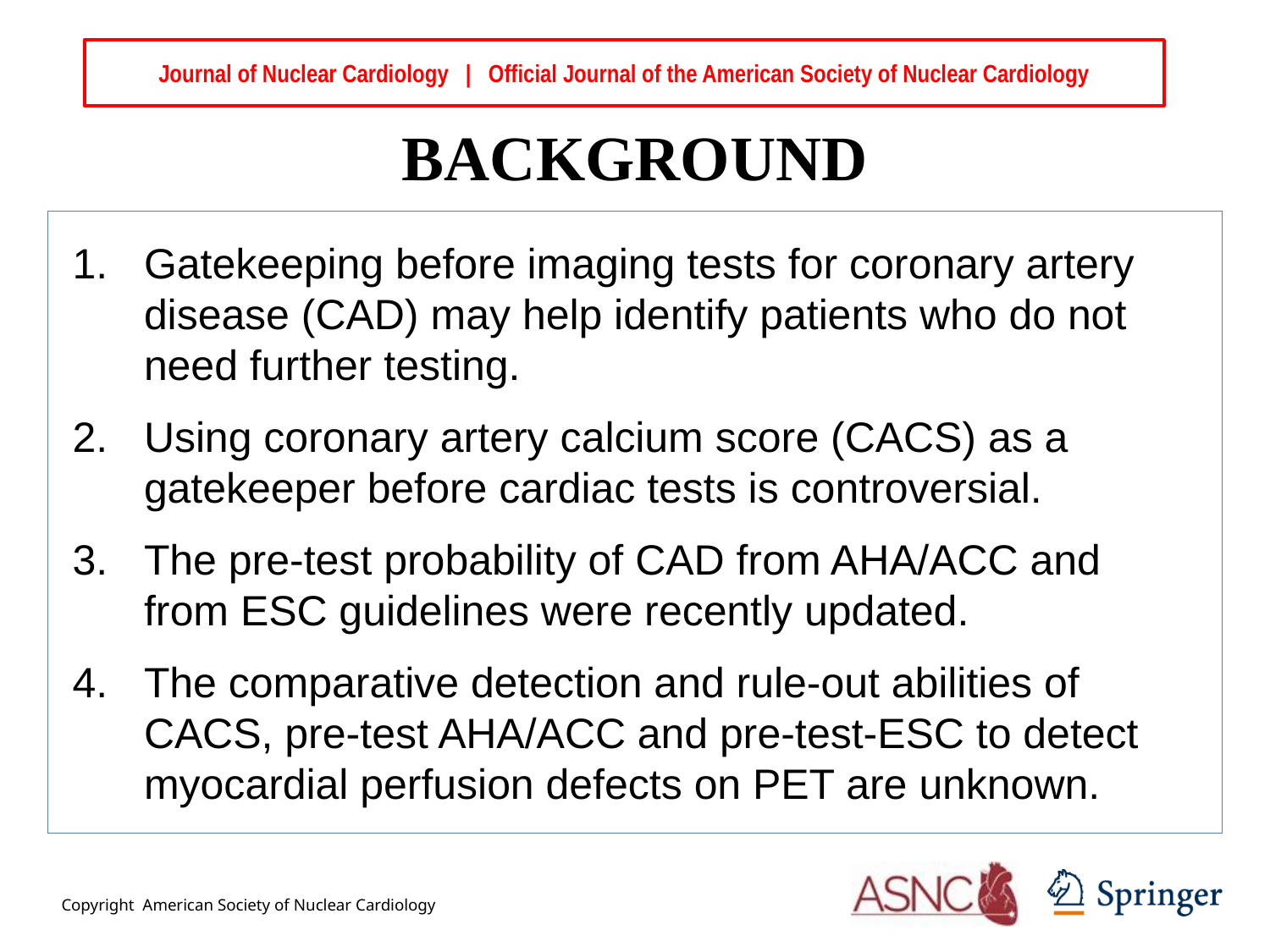

Journal of Nuclear Cardiology | Official Journal of the American Society of Nuclear Cardiology
# BACKGROUND
Gatekeeping before imaging tests for coronary artery disease (CAD) may help identify patients who do not need further testing.
Using coronary artery calcium score (CACS) as a gatekeeper before cardiac tests is controversial.
The pre-test probability of CAD from AHA/ACC and from ESC guidelines were recently updated.
The comparative detection and rule-out abilities of CACS, pre-test AHA/ACC and pre-test-ESC to detect myocardial perfusion defects on PET are unknown.
Copyright American Society of Nuclear Cardiology

## Slide 3
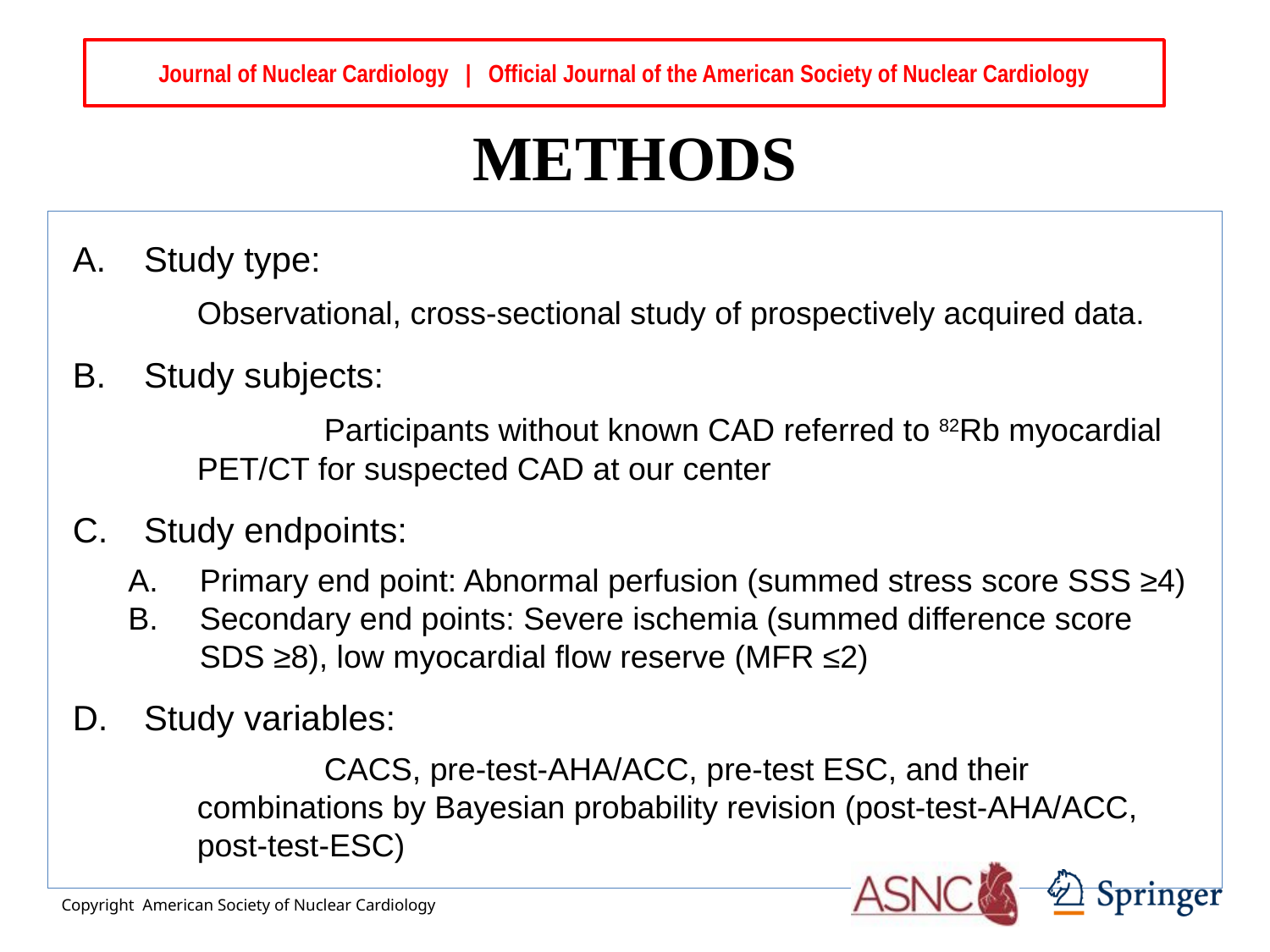

Journal of Nuclear Cardiology | Official Journal of the American Society of Nuclear Cardiology
# METHODS
Study type:
	Observational, cross-sectional study of prospectively acquired data.
Study subjects:
	Participants without known CAD referred to 82Rb myocardial PET/CT for suspected CAD at our center
Study endpoints:
Primary end point: Abnormal perfusion (summed stress score SSS ≥4)
Secondary end points: Severe ischemia (summed difference score SDS ≥8), low myocardial flow reserve (MFR ≤2)
Study variables:
	CACS, pre-test-AHA/ACC, pre-test ESC, and their combinations by Bayesian probability revision (post-test-AHA/ACC, post-test-ESC)
Copyright American Society of Nuclear Cardiology

## Slide 4
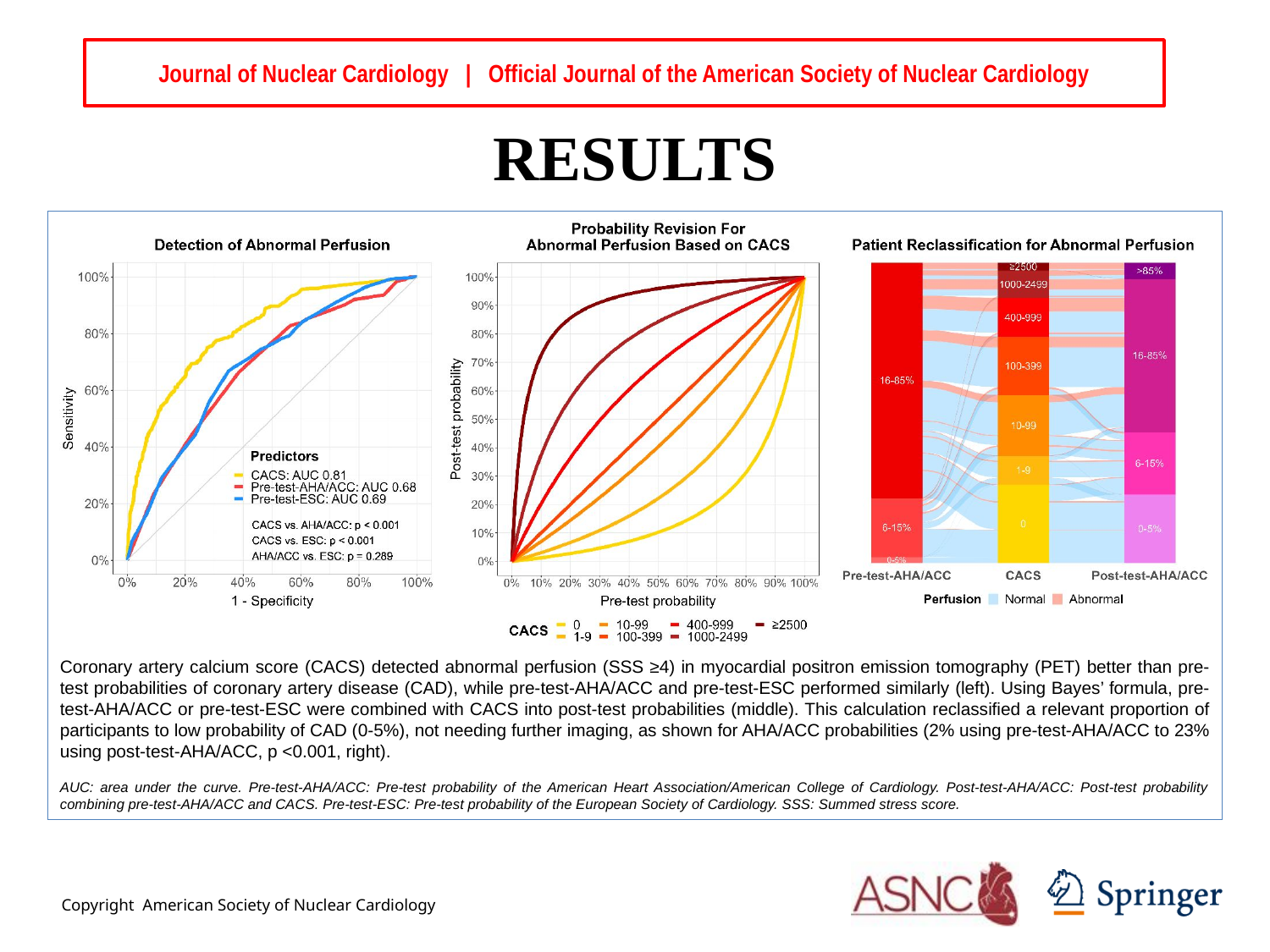

Journal of Nuclear Cardiology | Official Journal of the American Society of Nuclear Cardiology
# RESULTS
Coronary artery calcium score (CACS) detected abnormal perfusion (SSS ≥4) in myocardial positron emission tomography (PET) better than pre-test probabilities of coronary artery disease (CAD), while pre-test-AHA/ACC and pre-test-ESC performed similarly (left). Using Bayes’ formula, pre-test-AHA/ACC or pre-test-ESC were combined with CACS into post-test probabilities (middle). This calculation reclassified a relevant proportion of participants to low probability of CAD (0-5%), not needing further imaging, as shown for AHA/ACC probabilities (2% using pre-test-AHA/ACC to 23% using post-test-AHA/ACC, p <0.001, right).
AUC: area under the curve. Pre-test-AHA/ACC: Pre-test probability of the American Heart Association/American College of Cardiology. Post-test-AHA/ACC: Post-test probability combining pre-test-AHA/ACC and CACS. Pre-test-ESC: Pre-test probability of the European Society of Cardiology. SSS: Summed stress score.
Copyright American Society of Nuclear Cardiology

## Slide 5
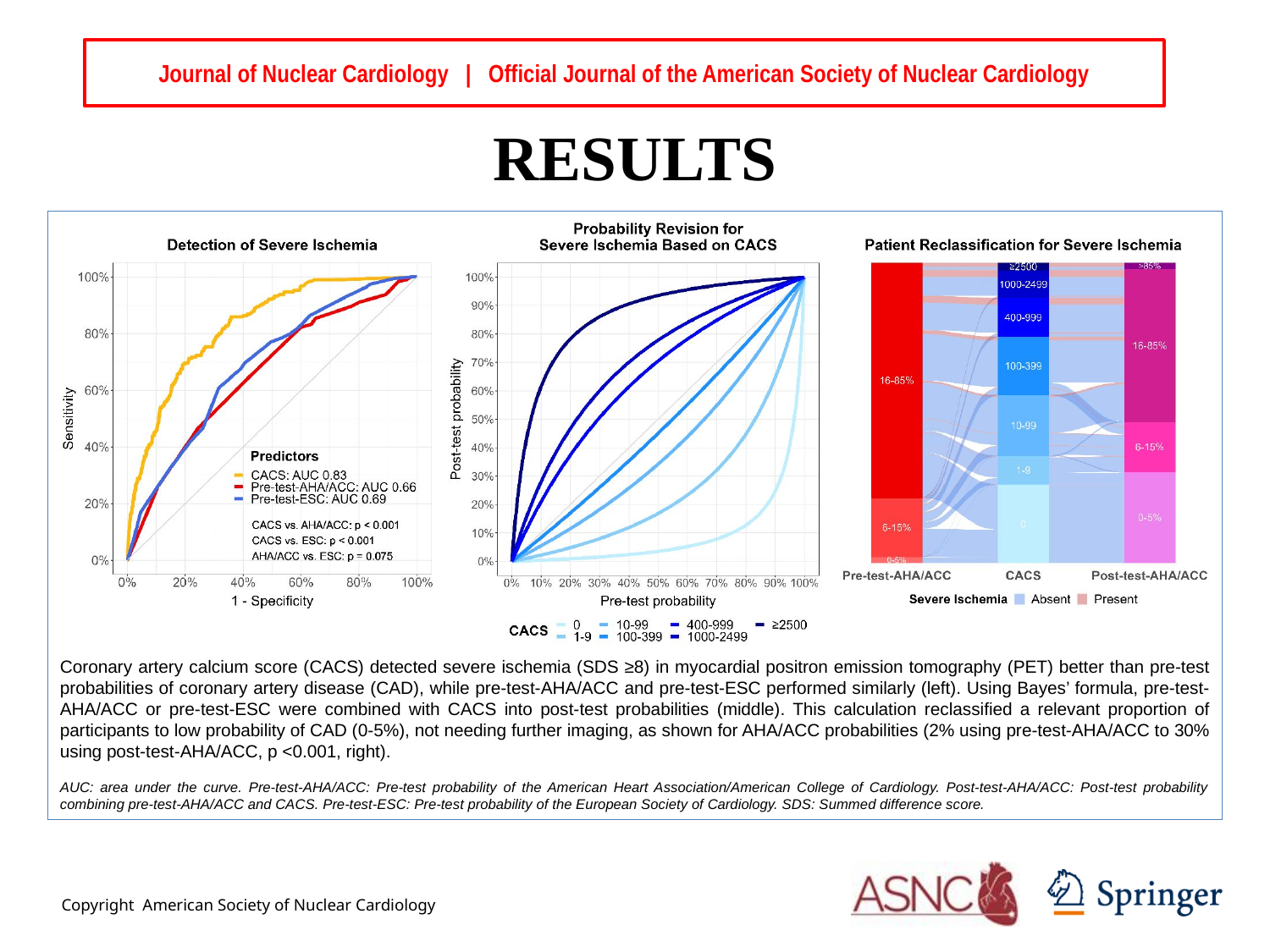

Journal of Nuclear Cardiology | Official Journal of the American Society of Nuclear Cardiology
# RESULTS
Coronary artery calcium score (CACS) detected severe ischemia (SDS ≥8) in myocardial positron emission tomography (PET) better than pre-test probabilities of coronary artery disease (CAD), while pre-test-AHA/ACC and pre-test-ESC performed similarly (left). Using Bayes’ formula, pre-test-AHA/ACC or pre-test-ESC were combined with CACS into post-test probabilities (middle). This calculation reclassified a relevant proportion of participants to low probability of CAD (0-5%), not needing further imaging, as shown for AHA/ACC probabilities (2% using pre-test-AHA/ACC to 30% using post-test-AHA/ACC, p <0.001, right).
AUC: area under the curve. Pre-test-AHA/ACC: Pre-test probability of the American Heart Association/American College of Cardiology. Post-test-AHA/ACC: Post-test probability combining pre-test-AHA/ACC and CACS. Pre-test-ESC: Pre-test probability of the European Society of Cardiology. SDS: Summed difference score.
Copyright American Society of Nuclear Cardiology

## Slide 6
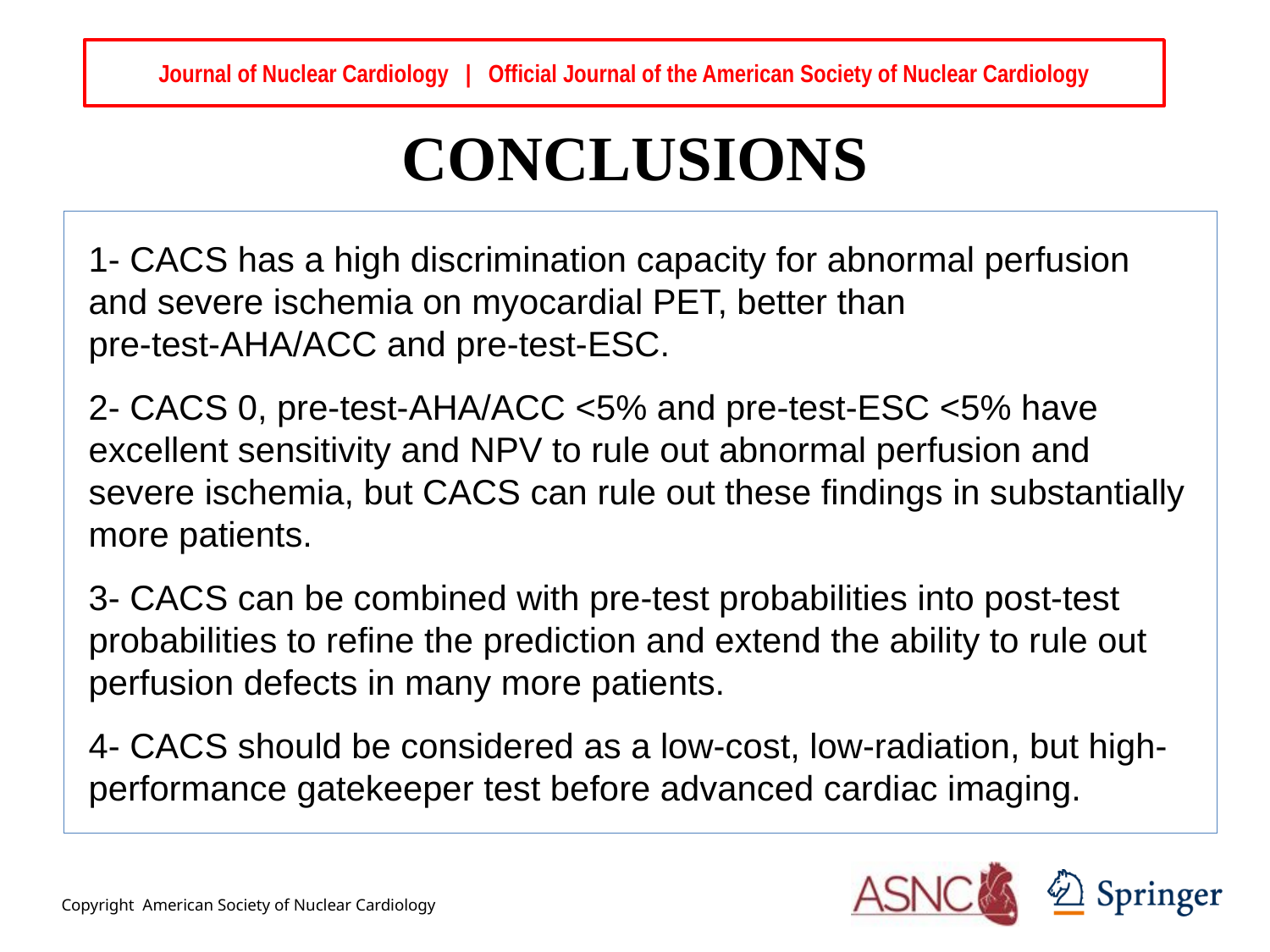

Journal of Nuclear Cardiology | Official Journal of the American Society of Nuclear Cardiology
# CONCLUSIONS
1- CACS has a high discrimination capacity for abnormal perfusion and severe ischemia on myocardial PET, better than pre-test-AHA/ACC and pre-test-ESC.
2- CACS 0, pre-test-AHA/ACC <5% and pre-test-ESC <5% have excellent sensitivity and NPV to rule out abnormal perfusion and severe ischemia, but CACS can rule out these findings in substantially more patients.
3- CACS can be combined with pre-test probabilities into post-test probabilities to refine the prediction and extend the ability to rule out perfusion defects in many more patients.
4- CACS should be considered as a low-cost, low-radiation, but high-performance gatekeeper test before advanced cardiac imaging.
Copyright American Society of Nuclear Cardiology
